# Novel Pyridinium Based Ionic Liquid Promoter for Aqueous Knoevenagel Condensation: Green and Efficient Synthesis of New Derivatives with Their Anticancer Evaluation

**DOI:** 10.3390/molecules27092940

**Published:** 2022-05-04

**Authors:** AbdElAziz A. Nayl, Wael A. A. Arafa, Ismail M. Ahmed, Ahmed I. Abd-Elhamid, Esmail M. El-Fakharany, Mohamed A. Abdelgawad, Sobhi M. Gomha, Hamada M. Ibrahim, Ashraf A. Aly, Stefan Bräse, Asmaa K. Mourad

**Affiliations:** 1 Department of Chemistry, College of Science, Jouf University, Sakaka 72341, Al Jouf, Saudi Arabia; waarafa@ju.edu.sa (W.A.A.A.); imibrahim@ju.edu.sa (I.M.A.); 2 Composites and Nanostructured Materials Research Department, Advanced Technology and New Materials Research Institute, City of Scientific Research and Technological Applications (SRTA-City), New Borg Al-Arab, Alexandria 21934, Egypt; ahm_ch_ibr@yahoo.com; 3 Protein Research Department, Genetic Engineering and Biotechnology Research Institute GEBRI, City of Scientific Research and Technological Applications (SRTA City), New Borg El-Arab, Alexandria 21934, Egypt; eelfakharany@srtacity.sci.eg; 4 Department of Pharmaceutical Chemistry, College of Pharmacy, Jouf University, Sakaka 72341, Al Jouf, Saudi Arabia; mhmdgwd@ju.edu.sa; 5 Chemistry Department, Faculty of Science, Cairo University, Giza 12613, Egypt; smgomha@iu.edu.sa; 6 Chemistry Department, Faculty of Science, Islamic University of Madinah, Madinah 42351, Al Jamiah, Saudi Arabia; 7 Chemistry Department, Faculty of Science, Fayoum University, Fayoum 63514, Egypt; hmi00@fayoum.edu.eg (H.M.I.); akk00@fayoum.edu.eg (A.K.M.); 8 Chemistry Department, Faculty of Science, Organic Division, Minia University, El-Minia 61519, Egypt; ashrafaly63@yahoo.com; 9 Institute of Organic Chemistry (IOC), Karlsruhe Institute of Technology (KIT), Fritz-Haber-Weg 6, 76133 Karlsruhe, Germany; 10 Institute of Biological and Chemical Systems—Functional Molecular Systems (IBCS-FMS), Director Hermann-von-Helmholtz-Platz 1, 76344 Eggenstein-Leopoldshafen, Germany

**Keywords:** Knoevenagel reaction, active methylene, ninhydrin, molecules, green chemistry, anticancer, docking study

## Abstract

Herein, a distinctive dihydroxy ionic liquid ([Py-2OH]OAc) was straightforwardly assembled from the sonication of pyridine with 2-chloropropane-1,3-diol by employing sodium acetate as an ion exchanger. The efficiency of the ([Py-2OH]OAc as a promoter for the sono-synthesis of a novel library of condensed products through DABCO-catalyzed Knoevenagel condensation process of adequate active cyclic methylenes and ninhydrin was next investigated using ultimate greener conditions. All of the reactions studied went cleanly and smoothly, and the resulting Knoevenagel condensation compounds were recovered in high yields without detecting the aldol intermediates in the end products. Compared to traditional strategies, the suggested approach has numerous advantages including mild reaction conditions with no by-products, eco-friendly solvent, outstanding performance in many green metrics, and usability in gram-scale synthesis. The reusability of the ionic liquid was also studied, with an overall retrieved yield of around 97% for seven consecutive runs without any substantial reduction in the performance. The novel obtained compounds were further assessed for their in vitro antitumor potential toward three human tumor cell lines: Colo-205 (colon cancer), MCF-7 (breast cancer), and A549 (lung cancer) by employing the MTT assay, and the findings were evaluated with the reference Doxorubicin. The results demonstrated that the majority of the developed products had potent activities at very low doses. Compounds comprising rhodanine (**5**) or chromane (**12**) moieties exhibited the most promising cytotoxic effects toward three cell lines, particularly rhodanine carboxylic acid derivative (**5c**), showing superior cytotoxic effects against the investigated cell lines compared to the reference drug. Furthermore, automated docking simulation studies were also performed to support the results obtained.

## 1. Introduction

**Figure 1:**
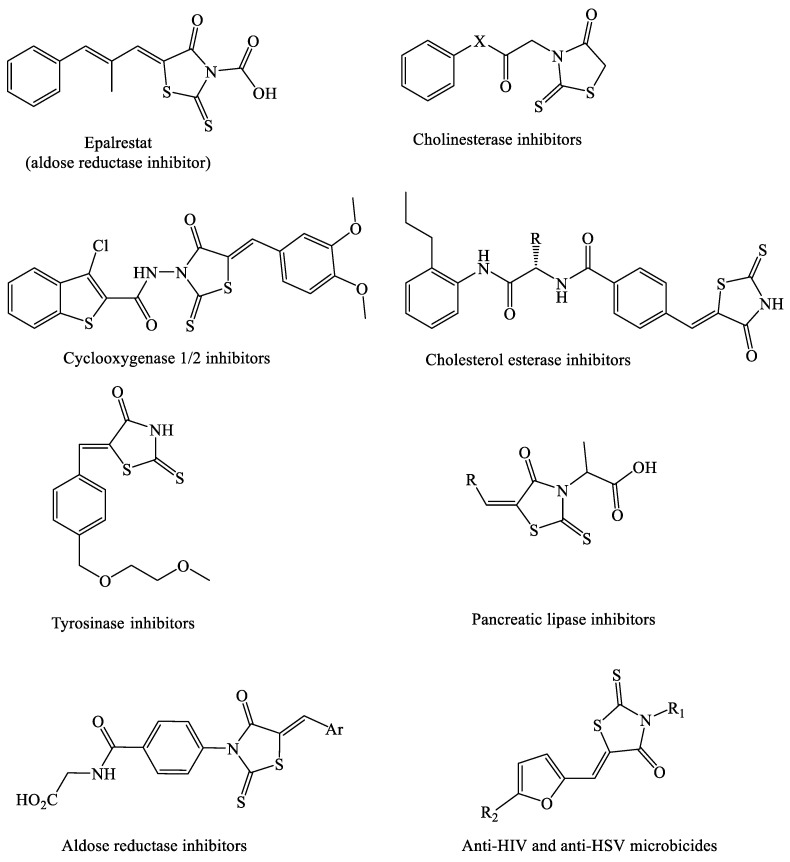
Biological activities of some selected examples of reported rhodanines [2,4,7,9,10,12,14,15].

Rhodanine and related compounds are known as scaffolds or templates to discover and develop drugs [1]. In the drug market, Epalrestat is known as a rhodanine acetic acid derivative to treat diabetic peripheral neuropathy. Epalrestat was reported to be generally used for a few adverse effects such as nausea, vomiting, and elevation of liver enzyme levels [2]. Rhodanine compounds have shown antidiabetic, anti-HIV, anti-infective, anti-Alzheimer, anticancer, antibacterial, anti-tubercular, and antifungal activities [3]. Compounds with a rhodanine structure have shown a broad range of biological activities such as carbonic anhydrase and acetylcholinesterase inhibitors [4], 15-lipoxygenase inhibitors [5], cyclooxygenase 1/2 inhibitors [6], anti-anxiety and anti-depressant [7], IKKβ-inhibitors [8], cholesterol esterase inhibitors [9], tyrosinase inhibitors [10], antiangiogenic [11], pancreatic lipase inhibitors [12], α-amylase inhibitors [13], aldose reductase inhibitors [14], anti-HIV and anti-HSV microbicides [15], and Dengue virus protease inhibitors [16], respectively (Figure 1).

The carcinogenic activities of rhodanines have yet to be thoroughly addressed. Aldose reductase (AR) has lately been reported to be an essential regulator of signaling pathways comprising oxidative stress-induced inflammatory diseases, notably cardiovascular diseases, cancers, sepsis, and asthma [17]. AR accumulation in inflammatory-mediated breast, liver, and colon tumors is increasing its potential as a targeted therapy [18]. Moreover, several reports have demonstrated that AR is essential in the drugs’ resistance to numerous carcinogenic medicines such as cisplatin, daunorubicin, and others through increasing metabolism, increasing efflux, or diminished penetration of medications [19]. As a consequence, inhibiting AR with medical therapy or genetic modification might be a unique strategy for treating drug-resistant tumors.

On binding to synthetic or natural ligands, peroxisome proliferator-activated receptors (PPARs) have displayed transcription mediators that control gene production and inhibition [20]. PPARs are involved in differential tissue distribution and mediate specific functions in early development, cell proliferation, differentiation, apoptosis, and metabolic homeostasis [20]. PPARγ and their ligands have vital roles in regulating both apoptotic pathways. PPARγ agonists (e.g., TZDs) have been shown to induce apoptosis in various cancer cells including lymphoma, multiple myeloma, bladder, gastric, esophageal, pancreatic, hepatoma, colon, breast, brain, and lung cancer cells [21]. Various works have shown that combining PPARγ agonists with anticancer agents can further sensitize tumor cells to apoptosis. PPARγ plays inhibitory roles in cell growth and proliferation by favoring cell differentiation [22]. Moreover, PPARγ, activated by natural and synthetic ligands, is a promising option for preventing and treating cancer. Knoevenagel condensation is a substantial reaction for generating carbon–carbon bonds via the interaction between active methylene and carbonyl derivatives [23,24]. The α,β-unsaturated carbonyl molecules created by the Knoevenagel reaction could be utilized to design essential precursors for a variety of high-value substances including pharmaceuticals, cosmetics, natural products, fine chemicals, and multifunction polymers [25,26]. Consequently, study on such reactions seems to have been a hot issue in organic syntheses, and sustainable protocols are necessitated. Recently, diverse catalytic approaches have been established for the Knoevenagel reaction employing some designing catalysts such as zeolites [27], polymers [28], functionalized fibers [29], graphene oxide [30], complexes [31], enzymes [32], nanoparticles [33], MOFs [34], and ionic liquids [35,36] have indicated wide applications. Catalysts have shown productivity of the reactions and potentially influence their selectivity [37,38,39]. Ionic liquids (ILs) have had a wide and cutting-edge influence during the last decade, developing potential research and technology [40]. They have essential roles in synthesis, catalysis, extraction, electrochemistry, analytics, biotechnology, material science, and other domains due to their highly tunable nature and extraordinary properties. Moreover, depending on the application, these properties might be adjusted by varying the cation and anion combinations [41]. Interestingly, ionic liquids are separated in various phases that reduce the use of combustible, volatile, and poisonous solvents and facilitate the work-up processes and avoid time-consuming purification procedures [40,42,43,44,45]. The base-catalyzed addition to a carbonyl motif in the Knoevenagel reaction is initiated by a nucleophilic attack accompanied by a protonation process [46]. On the other site, acid-catalyzed by the Knoevenagel reaction would cause the protonation of a carbonyl group that precedes via the attack of a weak nucleophile [26]. Therefore, introducing an ionic liquid with hydroxyl groups might cause an acceleration of the reaction pathway [47,48]. In pursuit of our works on the synthesis of novel ILs and their utilization as catalysts and/or solvents in diverse chemical conversions [43,48,49], we designed and prepared a new ionic liquid ([Py-2OH]OAc) with two hydroxyl groups. Under ultrasonication, the performance of novel IL was established for DABCO-catalyzed Knoevenagel reactions. The automated docking simulation studies were investigated to support the results obtained. The newly synthesized compounds were also screened for antiproliferative activity.

## 2. Experimental

The melting points were determined employing the Electrothermal IA9100 melting point instrument (UK), and the findings were given uncorrected. The IR spectra (KBr) from a Perkin-Elmer Spectrum One spectrometer were recorded. The NMR spectra were obtained by employing a Bruker AV instrument with 400 MHz. A Bruker Daltonics microTOF spectrometer was used to obtain high-resolution mass spectra (HRMS). The purity of substances was determined using analytical thin-layer chromatography (TLC) on an EM silica gel F254 sheet (0.2 mm). The sonication reactions were carried out in an SY5200DH–T ultrasound cleaner.

### 2.1. Synthesis of 1-(1,3-dihydroxypropan-2-yl)pyridin-1-ium Acetate [Py-2OH]OAc

In a closed vial, a mixture of pyridine (0.85 mL, 1.0 mmol) and 2-chloropropane-1,3-diol (0.11 g, 1.0 mmol) was sonicated at 70 °C for 3 h. The obtained crude product was purified by recrystallization from acetonitrile to produce the pure product (99%) as white crystals (**1**). Then, to the residue of **1** in EtOH (15.0 mL), NaOAc (0.082 g, 1.0 mmol) was introduced. The resulting solution was sonicated at 70 °C for 30 min, following which the solvent was removed under reduced pressure to yield the corresponding IL (**2**) with an almost quantitative yield.

White crystals; Yield 99%; mp 88–90 °C. IR (KBr): 3412-3108 (OH), 1683, (C=O), 1598 cm^−1^ (C=C). ^1^H NMR (400 MHz, DMSO-*d6*): *δ* = 9.17 (d, *J* = 5.2 Hz, 2H, Ar-*H*), 8.49 (t, *J* = 7.7 Hz, 1H, Ar-*H*), 8.20–8.17 (m, 2H, Ar-*H*), 5.59 (s, 2H, 2O*H*), 4.81–4.80 (m, 1H, C*H*), 3.74–3.69 (m, 4H, 2C*H*_2_), 2.23 ppm (s, 3H, C*H*_3_). ^13^C NMR (100 MHz, DMSO-*d6*): *δ* = 173.0 (*C*=O), 148.8, 147.3, 132.4 (Ar-*C*), 68.2 (*C*H), 60.0 (*C*H_2_), 26.3 ppm (*C*H_3_). HRMS, *m/z*: 236.0886 (M + Na). Anal. Calcd for C_10_H_15_NO_4_: C, 56.33; H, 7.09; N, 6.57%. Found: C, 56.39; H, 6.97; N, 6.51%.

### 2.2. Synthesis of Knoevenagel Condensation Products (***5a–d*, *8a–d*, *10a,b*, *12a,b*, *14a–d***, and ***16a,b***)

To a mixture of appropriate active methylene (1.0 mmol) in IL-H_2_O-DABCO (60–40%-1.0 mmol), 1.0 mmol of carbonyl compounds (**3**, **13**, and **15**) was added. The resulting solution was sonicated at 30 °C for 5 min (TLC). After cooling to room temperature, the obtained crystals were filtered off, dried, and crystallized from a proper solvent.

**2-(3-Methyl-4-oxo-2-thioxothiazolidin-5-ylidene)-1*H*-indene-1,3(2*H*)-dione (5a)**

Yellow crystals (EtOH); Yield 99%; mp 187–188 °C. IR (KBr): 1739, 1678 (C=O), 1593, (C=C), 1427 cm^−1^ (C=S). ^1^H NMR (400 MHz, CDCl_3_): *δ* = 7.92–7.90 (m, 4H, Ar-*H*), 3.35 ppm (s, 3H, C*H*_3_). ^13^C NMR (100 MHz, CDCl_3_): *δ* = 195.5 (*C*=S), 192.3, 167.5 (*C*=O), 148.2, 148.1, 139.8, 135.7, 128.2 (Ar-*C*), 34.1 ppm (*C*H_3_). HRMS, *m/z*: 311.9764 (M + Na). Anal. Calcd for C_13_H_7_NO_3_S_2_: C, 53.97; H, 2.44; N, 4.84%. Found: C, 53.92; H, 2.48; N, 4.78%.

**2-(3-Ethyl-4-oxo-2-thioxothiazolidin-5-ylidene)-1*H*-indene-1,3(2*H*)-dione (5b)**

Yellow crystals (dioxane); Yield 94%; mp 165–166 °C. IR (KBr): 1741, 1677 (C=O), 1601, (C=C), 1442 cm^−1^ (C=S). ^1^H NMR (400 MHz, DMSO-*d6*): *δ* = 8.07–7.93 (m, 4H, Ar-*H*), 4.21–4.16 (q, *J* = 7.2 Hz, 2H, C*H*_2_CH_3_), 1.22–1.18 ppm (t, *J* = 7.3 Hz, 3H, CH_2_C*H*_3_). HRMS, *m/z*: 325.9924 (M + Na). Anal. Calcd for C_14_H_9_NO_3_S_2_: C, 55.43; H, 2.99; N, 4.62%. Found: C, 55.46; H, 2.92; N, 4.57%.

**2-(5-(1,3-Dioxo-1,3-dihydro-2*H*-inden-2-ylidene)-4-oxo-2-thioxothiazolidin-3-yl)acetic acid (5c)**

Yellow crystals (dioxane); Yield 97%; mp 251–252 °C. IR (KBr): 3356-3012 (OH), 1739, 1708, 1685, (C=O), 1593 (C=C), 1440 cm^−1^ (C=S). ^1^H NMR (400 MHz, CDCl_3_): *δ* = 13.61 (br, 1H, CO_2_*H*), 7.67–7.58 (m, 4H, Ar-*H*), 4.60 ppm (s, 2H, C*H*_2_). HRMS, *m/z*: 355.9663 (M + Na). Anal. Calcd for C_14_H_7_NO_5_S_2_: C, 50.45; H, 2.12; N, 4.20%. Found: C, 50.49; H, 2.10; N, 4.17%.

**2-(3-Allyl-4-oxo-2-thioxothiazolidin-5-ylidene)-1*H*-indene-1,3(2*H*)-dione (5d)**

Yellow crystals (EtOH); Yield 99%; mp 145–146 °C. IR (KBr): 1739, 1678 (C=O), 1593, (C=C), 1427 cm^−1^ (C=S). ^1^H NMR (400 MHz, CDCl_3_): *δ* = 7.72-7.61 (m, 4H, Ar-*H*), 5.88–5.73 (m, 1H, CH_2_-C*H*=CH_2_), 5.19–5.10 (m, 2H, CH_2_-CH=C*H*_2_), 3.79–3.75 ppm (m, 2H, C*H*_2_-CH=CH_2_). HRMS, *m/z*: 337.9924 (M + Na). Anal. Calcd for C_15_H_9_NO_3_S_2_: C, 57.13; H, 2.88; N, 4.44%. Found: C, 57.17; H, 2.80; N, 4.38%.

**5-(1,3-Dioxo-1,3-dihydro-2*H*-inden-2-ylidene)imidazolidine-2,4-dione (8a)**

Brown crystals (dioxane); Yield 96%; mp 202–204 °C. IR (KBr): 3311, 3245 (NH), 1741, 1688 cm^−1^ (C=O). ^1^H NMR (400 MHz, DMSO-*d6*): *δ* = 11.01 (s, 1H, N*H*), 8.70 (s, 1H, N*H*), 7.92–7.90 ppm (m, 4H, Ar-*H*). ^13^C NMR (100 MHz, DMSO-*d6*): *δ* = 191.0 (*C*=S), 167.6, 160.1 (*C*=O), 134.5, 131.5, 127.8, 127.7, 123.2 ppm (Ar-*C*). HRMS, *m/z*: 242.0325 (M). Anal. Calcd for C_12_H_6_N_2_O_4_: C, 59.51; H, 2.50; N, 11.57%. Found: C, 59.54; H, 2.46; N, 11.50%.

**5-(1,3-Dioxo-1,3-dihydro-2*H*-inden-2-ylidene)-1-methylimidazolidine-2,4-dione (8b)**

Brown crystals (dioxane); Yield 96%; mp 192–193 °C. IR (KBr): 3197 (NH), 1732, 1672 cm^−1^ (C=O). ^1^H NMR (400 MHz, CDCl_3_): *δ* = 10.18 (s, 1H, N*H*), 7.73–7.71 (m, 4H, Ar-*H*), 2.90 ppm (s, 3H, C*H*_3_). HRMS, *m/z*: 279.0384 (M + Na). Anal. Calcd for C_13_H_8_N_2_O_4_: C, 60.94; H, 3.15; N, 10.93%. Found: C, 60.91; H, 3.19; N, 10.87%.

**2-(5-oxo-2-Thioxoimidazolidin-4-ylidene)-1*H*-indene-1,3(2*H*)-dione (8c)**

Yellow crystals (dioxane); Yield 95%; mp 222–224 °C. IR (KBr): 3354–3112 (NH), 1738, 1673 (C=O), 1449 cm^−1^ (C=S). ^1^H NMR (400 MHz, CDCl_3_): *δ* = 11.59 (s, 1H, N*H*), 11.40 (s, 1H, N*H*), 7.86–7.75 ppm (m, 4H, Ar-*H*). HRMS, *m/z*: 280.9992 (M). Anal. Calcd for C_12_H_6_N_2_O_3_S: C, 55.81; H, 2.34; N, 10.85%. Found: C, 55.77; H, 2.39; N, 10.81%.

**2-(2-Imino-3-methyl-5-oxoimidazolidin-4-ylidene)-1*H*-indene-1,3(2*H*)-dione (8d)**

Brown crystals (EtOH); Yield 95%; mp 169–170 °C. IR (KBr): 3318, 3109 (NH), 1741, 1679 cm^−1^ (C=O). ^1^H NMR (400 MHz, DMSO-*d6*): *δ* = 10.00 (br, 1H, N*H*), 8.40 (br, 1H, N*H*), 7.86–7.84 (d, *J* = 8.1 Hz, 4H, Ar-*H*), 2.79 ppm (s, 3H, C*H*_3_). HRMS, *m/z*: 279.0541 (M + Na). Anal. Calcd for C_13_H_9_N_3_O_3_: C, 61.18; H, 3.55; N, 16.46%. Found: C, 61.10; H, 3.58; N, 16.41%.

**2-(3-Methyl-5-oxo-1,5-dihydro-4*H*-pyrazol-4-ylidene)-1*H*-indene-1,3(2*H*)-dione (10a)**

Red crystals (dioxane); Yield 95%; mp 195–196 °C. IR (KBr): 3389 (NH), 1738, 1671 cm^−1^ (C=O). ^1^H NMR (400 MHz, CDCl_3_): *δ* = 11.30 (br, 1H, N*H*), 7.67–7.58 (m, 4H, Ar-*H*), 2.20 ppm (s, 3H, C*H*_3_). HRMS, *m/z*: 240.0536 (M). Anal. Calcd for C_13_H_8_N_2_O_3_: C, 65.00; H, 3.36; N, 11.66%. Found: C, 64.92; H, 3.39; N, 11.60%.

**2-(5-Oxo-3-phenylisoxazol-4(5*H*)-ylidene)-1*H*-indene-1,3(2*H*)-dione (10b)**

Brown crystals (DMF); Yield 98%; mp 240–242 °C. IR (KBr): 1745, 1730 cm^−1^ (C=O). ^1^H NMR (400 MHz, DMSO-*d6*): *δ* = 7.98 (d, *J* = 8.1 Hz, 2H, Ar-*H*), 7.88 (d, *J* = 7.8 Hz, 2H, Ar-*H*), 7.71 (d, *J* = 5.8 Hz, 2H, Ar-*H*), 7.39 ppm (t, *J* = 7.7 Hz, 3H, Ar-*H*). HRMS, *m/z*: 303.0534 (M). Anal. Calcd for C_18_H_9_NO_4_: C, 71.29; H, 2.99; N, 4.62%. Found: C, 71.22; H, 3.04; N, 4.56%.

**2-(4-Oxochroman-3-ylidene)-1*H*-indene-1,3(2*H*)-dione (12a)**

Brown crystals (dioxane); Yield 94%; mp 311–313 °C. IR (KBr): 1738, 1698 cm^−1^ (C=O). ^1^H NMR (400 MHz, DMSO-*d6*): *δ* = 8.07–7.96 (m, 4H, Ar-*H*), 7.79 (d, *J* = 7.7 Hz, 2H, Ar-*H*), 7.47 (t, *J* = 8.1 Hz, 1H, Ar-*H*), 7.30 (d, *J* = 7.9 Hz, 1H, Ar-*H*), 4.69 ppm (s, 2H, C*H*_2_). HRMS, *m/z*: 290.0582 (M). Anal. Calcd for C_18_H_10_O_4_: C, 74.48; H, 3.47%. Found: C, 74.51; H, 3.43%.

**2-(6-Fluoro-4-oxochroman-3-ylidene)-1*H*-indene-1,3(2*H*)-dione (12b)**

Brown crystals (dioxane); Yield 98%; mp 277–279 °C. IR (KBr): 1741, 1726 cm^−1^ (C=O). ^1^H NMR (400 MHz, DMSO-*d6*): *δ* = 7.89–7.79 (m, 4H, Ar-*H*), 7.34–7.30 (m, 2H, Ar-*H*), 7.08 (d, *J* = 7.7 Hz, 1H, Ar-*H*), 4.77 ppm (s, 2H, C*H*_2_). ^13^C NMR (100 MHz, DMSO-*d6*): *δ* = 194.2, 180.0 (*C*=O), 149.7 (d, *J* = 247 Hz), 147.2, 143.9, 139.6, 137.8, 125.3 (d, *J* = 26 Hz), 122.2, 121.6, 115.4, 113.8, 108 (d, *J* = 14 Hz) (Ar-*C*), 64.7 ppm (*C*H_2_). ^19^F NMR (376 MHz, DMSO-*d6*): *δ* = −125.91 ppm. HRMS, *m/z*: 307.0409 (M-H). Anal. Calcd for C_18_H_9_FO_4_: C, 70.13; H, 2.94%. Found: C, 70.17; H, 2.89%.

### 2.3. MTT Assay for Cell Viability/Proliferation

Cell lines: ATCC provided the investigated cells; Colo-205 (colon cancer), MCF-7 (breast cancer), and A549 (lung cancer) via Holding firm for bio-supplies and vaccinations (VACSERA) in Cairo, Egypt. The MTT (3-(4,5-dimethylthiazol-2-yl)-2,5-diphenyl tetrazolium bromide) analyses were employed to evaluate the compounds’ cytotoxic effects. In 96-well plates, cells (1 × 104/well) were plated in 100.0 mL of medium/well. After overnight incubation, the cells were handled with various drug doses in RPMI 1640 with 10% FBS for 24 h. MTT was then introduced, and the cells were maintained for four hours. MTT-formazan was prepared in 150.0 mL DMSO and stirred for 10 min. At a test wavelength of 490 nm, the absorbance was determined using an ELISA reader. Every experiment was carried out three times in total. The IC_50_ is the concentration of the substance that delivers a 50% growth inhibitory value.

### 2.4. Protocol of Docking Studies

Automated docking simulation studies were performed using Molecular Operating Environment (MOE^®^) version 2014.09. The X-ray crystallographic structures of the target AKR1B10 and PPAR*γ* from the protein data bank (PDB: 4JIH, 7E0A) were obtained from the protein data bank. The target compounds were constructed into a 3D model using the builder interface of the MOE^®^ program after checking their structures and the formal charges on atoms by 2D depiction.

## 3. Results and Discussion

**Scheme 1:**
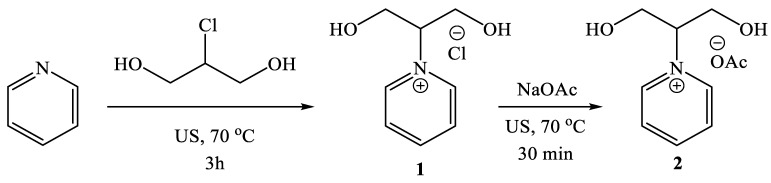
Sono-synthesis of ionic liquid [Py-2OH]OAc (**2**).

**Scheme 2:**
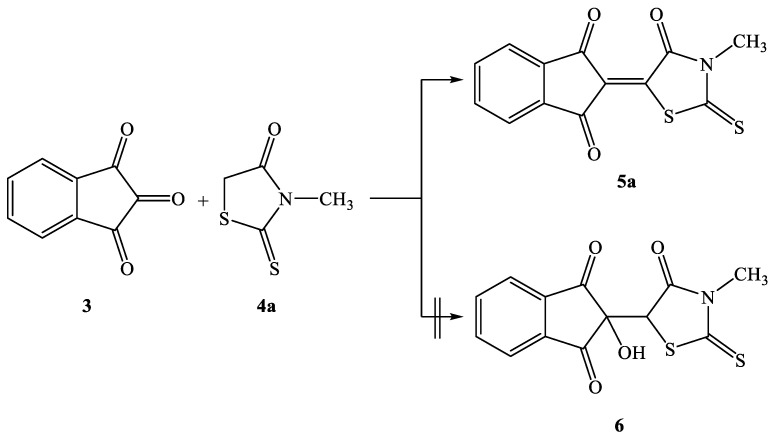
Synthesis of 2-(3-methyl-4-oxo-2-thioxothiazolidin-5-ylidene)-1*H*-indene-1,3(2*H*)-dione (**5a**).

**Scheme 3:**
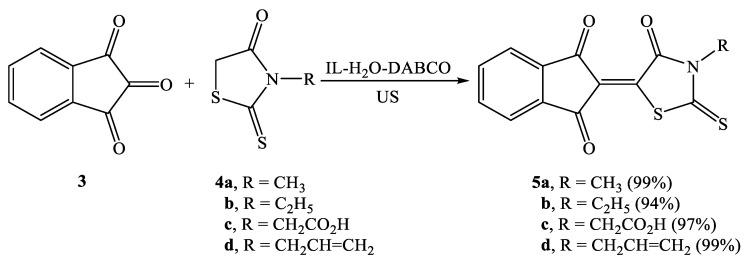
Ultrasound-assisted protocol for the preparation of **5a**–**d**.

**Scheme 4:**
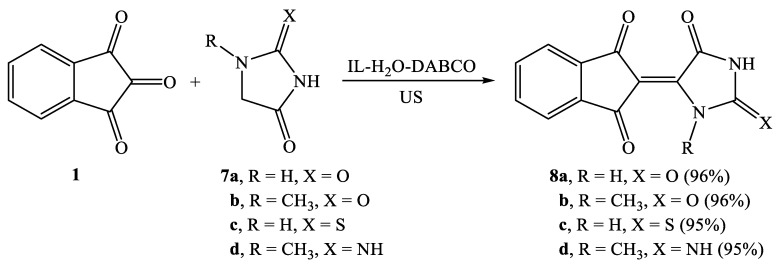
Ultrasound-assisted protocol for the preparation of **8a**–**d**.

**Scheme 5:**
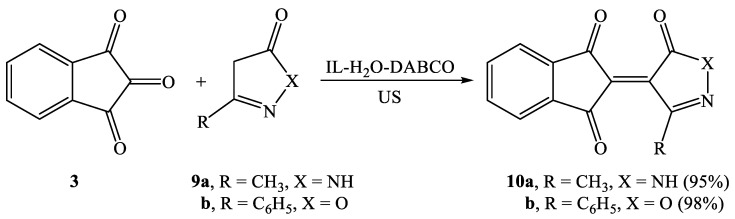
Ultrasound-assisted protocol for the preparation of **10a,b**.

**Scheme 6:**
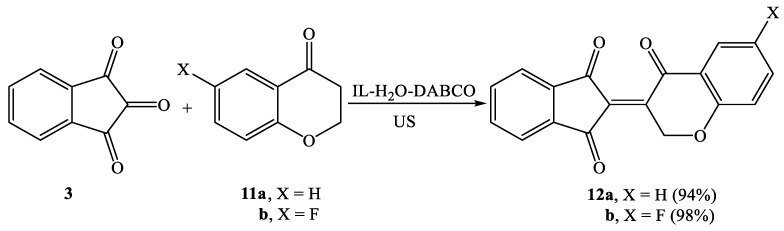
Ultrasound-assisted protocol for the preparation of **12a,b**.

Lately, water has been utilized to promote organic reactions as an eco-sustainable solvent [35,50]. The homogeneous solution comprised of water and ionic liquid could exhibit superior characteristics to pure water or ionic ILs, encouraging their utilization in organic synthesis. Accordingly, the IL comprising two hydroxyl groups, 1-(1,3-dihydroxypropan-2-yl)pyridin-1-ium chloride ([Py-2OH]Cl), was effectively delivered via the reaction displayed in Scheme 1. The quaternization of 2-chloropropane-1,3-diol with pyridine (Scheme 1) occurred under ultrasonic irradiation conditions (70 °C). Notwithstanding, the reaction afforded a poor yield on performing the reaction in an opened vessel. In contrast, the reaction yield was improved by employing the reaction in a closed vial. To study the temperature impact, this reaction (Scheme 1) was repeated at three different temperatures viz 50, 70, and 90 °C, and the optimal conversion (99% in 3 h) was observed at 70 °C (at 90 °C, neither the reaction yield nor the rate was enhanced). In other respects, water-soluble ILs comprising chloride ions are potentially liberated HCl in water due to hydrolysis [51,52], presenting a significant hazard of water pollution. In the perspective of environmental issues and ILs solubility, the halogen-free IL ([Py-2OH]OAc, **2**) was assembled through the reaction of IL (**1**) with sodium acetate as the anion exchanger (Scheme 1). The molecular structure of the synthesized IL (**2**) was achieved by HRMS and NMR spectroscopies (See Supplementary Materials). The designed ionic liquid ([Py-2OH]OAc, **2**) was employed as a promoter in the DABCO-catalyzed Knoevenagel reactions between various cyclic active methylene derivatives and ninhydrin (Scheme 2, Scheme 3, Scheme 4, Scheme 5 and Scheme 6).

To commence, we selected the widely available ninhydrin (**3**, 1.0 mmol) and 3-methylrhodanine (**4a**, 1.0 mmol) as model reactants to evaluate the efficiency of the constructed ionic liquid [Py-2OH]OAc (Scheme 2).

To evaluate the model reaction (Scheme 2) from the green chemistry standpoint, the ultrasound conditions were coupled under regimes of ionic liquid. After that, diversified parameters were extensively studied to achieve the optimal conditions including energy source, reaction duration, temperature, bases, IL loading, and solvents. Through the sonication (30 °C) of the template reactants in IL-H_2_O (1:1 equiv), a moderate yield of the hitherto unreported 2-(3-methyl-4-oxo-2-thioxothiazolidin-5-ylidene)-1*H*-indene-1,3(2*H*)-dione (**5a**) was obtained (Table 1, entry 1), along with traces of the aldol derivative (**6**, HRMS; *m/z*: 306.9972, corresponding to C_13_H_9_NO_4_S_2_) while the targeted product (**5a**) was not detected in the reaction profile (TLC) when performing the reaction in an aqueous medium (Table 1, entry 2). Interestingly, once the model reaction was carried out utilizing IL-H_2_O-DABCO (3 g:3 mL:1.0 mmol), the reaction profile was quite clear, and the Knoevenagel product (**5a**) was generated as the sole product (Scheme 2) without detecting the aldol product (**6**) (Table 1, entry 3). When organic bases including piperidine, hexamethylenetetramine (HMTA), or triethylamine (TEA) were employed rather than DABCO, moderate (75%) to good (83%) yields were achieved (Table 1, entries 4–6). Besides, a moderate yield was detected by employing the inorganic weak base K_2_CO_3_ (Table 1, entry 7). Unfortunately, when KOH was introduced, only 56% yield was achieved (Table 1, entry 8) with an unclear reaction profile. Furthermore, reducing the DABCO amount to 0.5 equiv diminished the productivity while increasing its amount to 2.0 equiv, so both the reaction yield and rate were unaffected. To further emphasize that the improvement resulted from the IL–H_2_O–DABCO system, IL–H_2_O and H_2_O–DABCO were also employed (Table 1, entries 1 and 9). The results displayed that, in both cases, the yields were diminished. Consequently, DABCO will be performed as the best basic candidate in the subsequent studies. Various solvents (Table 1, entries 10–12) were also screened, and water scored a superior yield within a short reaction time. This finding suggests that the current reaction (Scheme 2) belongs to the “on water” category and is therefore environmentally-friendly [53]. The reaction productivity was reduced when the model reaction (Scheme 2) was carried out at room temperature (23 °C) (Table 1, entry 13). In addition, increasing the temperature from 30 to 40 °C yielded the derivative **5a** in almost similar yield (88%) albeit with a prolonged time (Table 1, entry 14). Both reaction productivity and rate were not enhanced by gradually increasing the reaction temperature from 60 to 80 °C (Table 1, entries 15 and 16), thus establishing 30 °C is the optimal temperature. Furthermore, the proportion of IL (**2**) utilized in the process (Scheme 2) was investigated concerning the water content. An excellent yield (99%) was obtained in 5 min when the used proportion of IL:H_2_O was 60:40% (Table 1, entry 17). Decreasing the percentage of IL slightly diminished the yield, and the time became considerably longer (Table 1, entry 18). This might be because using a high percentage of water reduces the reactants’ dissolution, a fact confirmed by observing a considerable amount of insoluble reactants in the reaction medium whereas increasing the percentage of IL by more than 60% did not improve the reaction rate or yield (Table 1, entry 19). Besides, employing the model reaction in less than 5 min diminished the yield. Furthermore, performing the model reaction under conventional conditions resulted in diminishing the reaction yield (Table 1, entry 20). According to the above studies (Table 1), sonication (80%) of **4a** (1.0 mmol), ninhydrin (1.0 mmol), and DABCO (1.0 mmol) in H_2_O-IL solvent (40–60%) at 30 °C for 5 min was identified as the optimal condition for the model reaction (Scheme 2, Table 1, entry 17). The noticeable efficiency of the IL might be attributed to the presence of two hydroxyl groups that acted as a protic additive in promoting the reaction.

After effectively developing an optimal approach for the template reaction, we moved on to investigate the reaction’s scope and the variety of the substrates. Thus, in the next study, a varied array of functionalized cyclic active methylenes (**4**, **7**, **9**, and **11**) underwent rapid Knoevenagel reactions with ninhydrin (**3**) to produce hitherto unknown compounds (**5**, **8**, **10**, and **12**) in nearly quantitative yields (up to 99%, Scheme 3, Scheme 4, Scheme 5 and Scheme 6). Generally, rhodanines such as **4a**–**d** provided marginally higher yields of the respective products than the remaining active cyclic methylenes.

Within the rhodanine series, it was found that the electronic structure of the substituents has a minimal influence on the reaction performance. Both electron-releasing motifs (*N*-methyl and *N*-ethyl) and electron-attracting motifs (*N*-allyl and *N*-acetic acid) were amenable to the reaction (Scheme 3).

In addition, the reaction smoothly proceeded with imidazolidinone derivatives (**7a**–**d**) and afforded Knoevenagel condensation products (**8a**–**d**) in almost quantitative yields (Scheme 4).

Switching to pyrazolone and oxazolone derivatives (**9a,b**), the reactions also proceeded effectively and provided the targeted products with excellent yields (Scheme 5).

Interestingly, chroman-4-ones (**11a**,**b**) performed effective and potent reactions with ninhydrin, yielding the condensed compounds **12a** and **12b** in 94 and 98% yields, respectively (Scheme 6). The molecular structures of all the newly assembled compounds (**5**, **8**, **10**, and **12**) were evaluated utilizing IR, NMR spectroscopy as well as HRMS (see Supplementary Materials).

Having synthesizing targeted Knoevenagel products efficiently, we next evaluated the scalability of the conventional approach by substituting ninhydrin with other synthons such as isatin (Table 2) [54,55,56] and acenaphthenequinone (Table 3) [55,57].

Interestingly, the present method proved to be precisely as efficacious as in the instance of ninhydrin, and the related products were efficiently synthesized in high yields (Table 2 and Table 3). Table 2 and Table 3 demonstrate a comparison of the present approach with the reported procedures for the preparation of **14a**–**d** and **16a,b** to show the merits and efficiency of the present work. In the perspective of reaction time, yield, and yield economy, utilizing [Py-2OH]AcO under ultrasound conditions is undoubtedly the optimum alternative for such a Knoevenagel reaction.

**Scheme 7:**
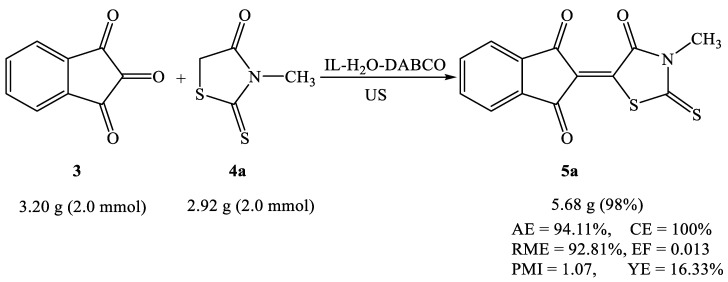
Scaled up the preparation of derivative **5a**.

In an attempt to assess the feasibility of scaling up the aforesaid procedure, the template reaction (Scheme 7) was carried out on a gram scale employing the optimal conditions that in turn yielded the required product (**5a**) in superior yield (98%; Scheme 7). Moreover, to assess the sustainability of the established procedure, various environmental metrics [26,58] such as atom economy (AE), yield economy (YE), environmental factor (EF), carbon efficiency (CE), reaction mass efficiency (RME), and process mass intensity (PMI) were also addressed (Scheme 7). As displayed in Scheme 7, the reaction scored the required criteria (lower values of EF and PMI, and higher values of RME, AE, CE, and YE). Therefore, it might be considered as a sustainable and eco-friendly procedure.

**Figure 2:**
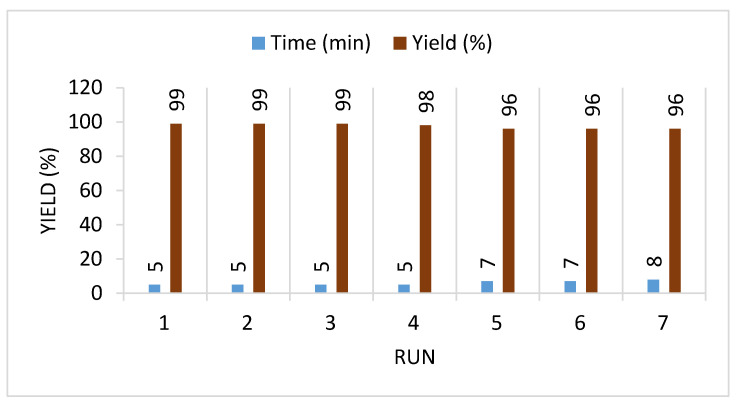
The evaluation of the reusability of the IL (**2**) for the preparation of **5a**.

Furthermore, to evaluate the feasibility of IL (**2**) reuse, the synthesis of **5a** was selected for this assignment. The reusability of the IL (2) was studied by repeating the model reaction under optimal conditions (Scheme 2). The obtained product was separated through simple filtration following every run, and the IL was retrieved by removing water under vacuum and reused in the next run. The IL was evidenced to be highly stable (^1^H NMR after the seventh run was the same as the original one) at optimum conditions and could be recycled up to seven cycles without a considerable loss of potency (Figure 2).

**Scheme 8:**
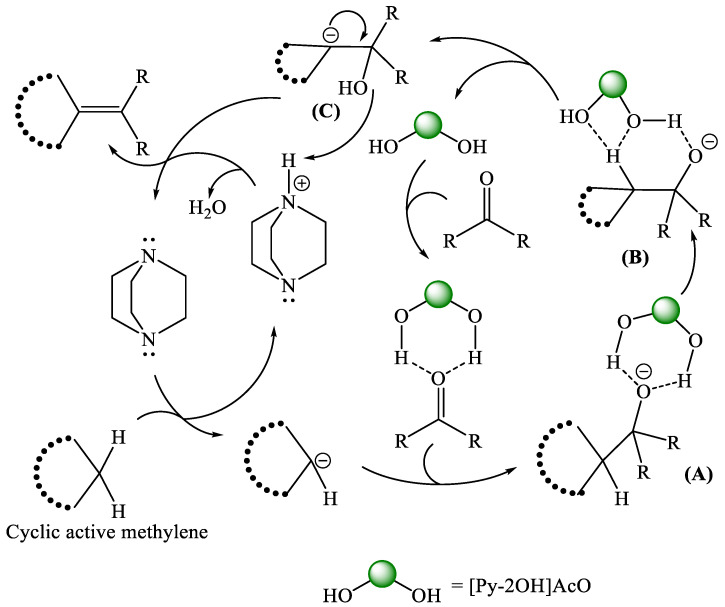
A possible reaction mechanism.

According to the obtained results, a suggested mechanism for this condensation reaction has been suggested (Scheme 8). At first, a carbanion was formed due to abstracting the acidic proton from the active methylene motif by DABCO. Simultaneously, the polarity of the carbonyl group was enhanced via hydrogen bond formation with the two-hydroxyl groups of the IL. Second, the generated carbanion underwent a nucleophilic attack at the protonated carbonyl motif, resulting in the adduct **A**. Furthermore, the IL accelerated the reaction through the proton transfer from the active methylene to the alkoxide (**B**), resulting in intermediate **C**. Finally, DABCO-quaternary ammonium proton facilitated the removal of a water molecule and the generation of the olefinic double bond (Scheme 8).

### 3.1. Cytotoxic Activity

**Figure 3:**
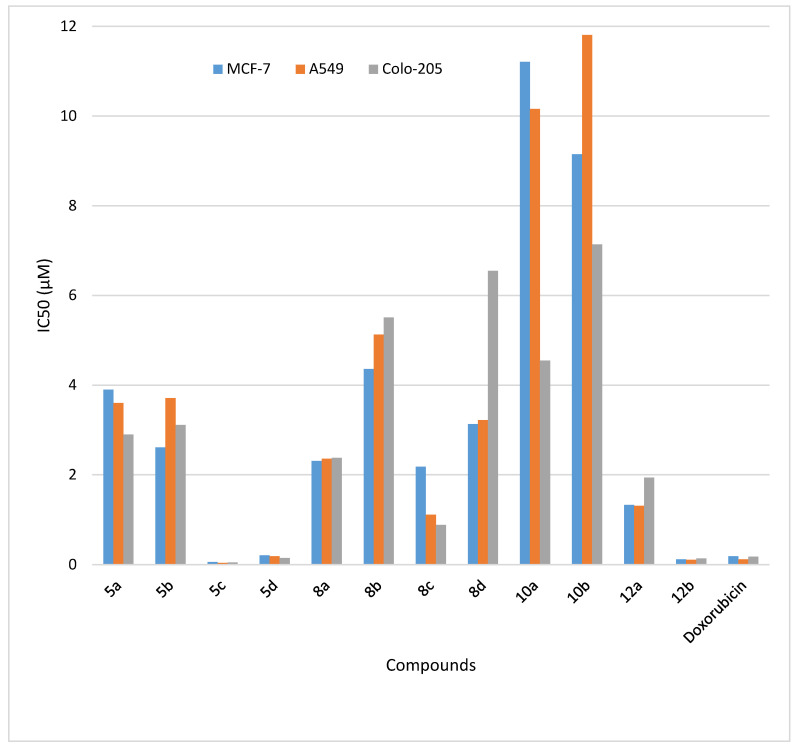
The in vitro activity of the newly synthesized derivatives against MCF-7, A549, and Colo-205 human cancer cell lines.

Utilizing the MTT test [59], the newly assembled Knoevenagel compounds (**5a**–**d**, **8a**–**d**, **10a**,**b**, and **12a**,**b**) were evaluated for their in vitro antitumor effects against an array of human cancer cell lines, including Colo-205 (colon cancer), MCF-7 (breast cancer), and A549 (lung cancer). Table 4 and Figure 3 display the IC_50_ (compound concentration that causes a 50% reduction in cell proliferation) measurements for the produced compounds employing Doxorubicin as a reference drug. The antitumor activity results suggested that the majority of the investigated derivatives demonstrated moderate to excellent potency, with IC_50_ values ranging from 0.04 to 11.81 μM. Derivatives **5**, **8**, and **12** demonstrated the most antiproliferative potential toward the three cell lines (IC_50_: 0.04–5.51 M). Of these, the efficiency of compound **5c** was approximately 3-fold more than that of Doxorubicin. Further, derivatives **10a** and **10b** were especially effective at inhibiting Colo-205 cell proliferation.

### 3.2. Structure–Activity Relationship (SARs)

According to the molecular structures of the newly assembled compounds, derivatives **5**–**12** could be categorized into four classes: **A** (**5a**–**d**), **B** (**8a**–**d**), **C** (**10a**,**b**), and **D** (**12a**,**b**). Regarding series **A**, derivative **5c** with an acetic acid motif was observed to be the most potent molecule of all series against the three investigated cells (IC_50_ varied between 0.04 and 0.06 μM) and demonstrated superior in vitro inhibition effects than Doxorubicin. Mechanistically, such carboxylic acids suppressed tube-forming activity induced by vascular endothelial growth factor (VEGF) and thus reduced the tumor growth [60]. Furthermore, compound **5d** exhibited similar antitumor activity to the reference drug (IC_50_ varied between 0.15 and 0.21 μM) against the three examined cell lines, confirming the beneficial effect of the allyl group in the treatment of cancer [61]. The introduction of alkyl motifs (methyl, **5a** or ethyl, **5b**) to the rhodanine nucleus reduced the in vitro inhibitory effects against three cell lines marginally. When the rhodanine motif was replaced with an imidazole nucleus, the resultant series (class B) effectively contributed to the anticancer activity of the assessed strains. In this series, introducing an alkyl substituent such as methyl (**8b** or **8d**) instead of hydrogen atom (**8a** or **8c**) resulted in a slight diminishing in the inhibitory activities while the introduction of the thione motif (**8c**) resulted in a remarkable improvement in the cytotoxicity, particularly toward Colo-205 with IC_50_ of 0.89 μM. This might be due to the existence of a polar thiocarbonyl moiety that may enhance their interaction with the hydrophilic binding sites [61]. According to the obtained values of IC_50_ (Table 4), it could be inferred that rhodanine derivatives (class A) exhibit superior pharmacodynamic impacts than the imidazoles (class B). The pyrazole derivative **10a** (class C) showed significantly high activity (for A549 and Colo-205), which was slightly decreased upon being replaced by an isoxazole ring (**10b**). This might be attributed to the introduction of a more bulky moiety such as the benzene ring (in derivative **10b**), leading to an enhancement in the steric hindrance and therefore reducing the interaction to the target [62]. Compounds **12a** and **12b** (class D) represented a noteworthy exception, demonstrating comparable antiproliferative potency to the reference drug, indicating that the presence of a chromone ring might considerably enhance potency toward the examined strains. In this series, replacing the hydrogen atom (**12a**) with a substituent such as a fluoro (F) atom (**12b**) resulted in a significant increase in activities (Table 4 and Figure 3). This result supported the previous study that the presence of a lipophilic fluoro substituent improved the antiproliferative potential [61].

### 3.3. Docking Study

**Figure 4:**
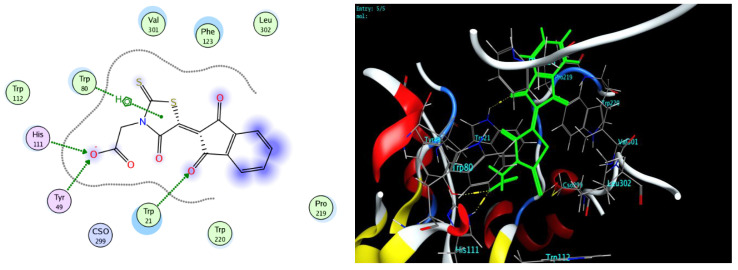
2D and 3D docking of compound **5c** with AKR1B10.

Enzyme-ligand docking was investigated for three prepared derivatives **5c,d, 12b** to predict the binding interactions between the novel prepared derivatives and AKR1B10 and PPAR*γ* protein that resulted from the protein data bank (PDB: 4JIH, 7E0A), respectively, by Molecular Operating Environment (MOE^®^) version 2014.09 (Table 5). The investigated derivatives were successfully docked into the active sites of AKR1B10 and PPAR*γ* protein compared with Epalrestat as a reference compound (Figure 4).

**Figure 5:**
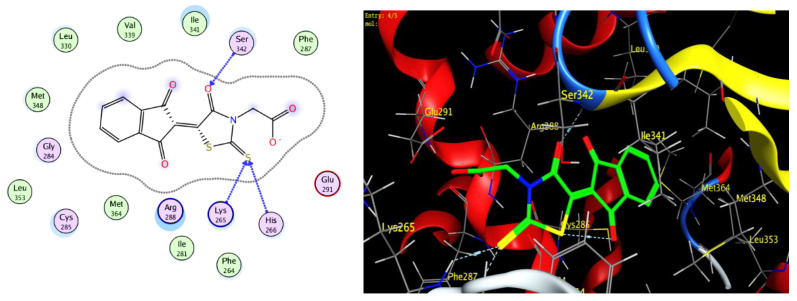
2D and 3D docking of compound **5c** with PPAR*γ*.

On the other hand, compound **5c** binding with PPAR*γ* shows three hydrogen bonds with SER 342, LYS 265, and HIS 266 (Figure 5).

**Figure 6:**
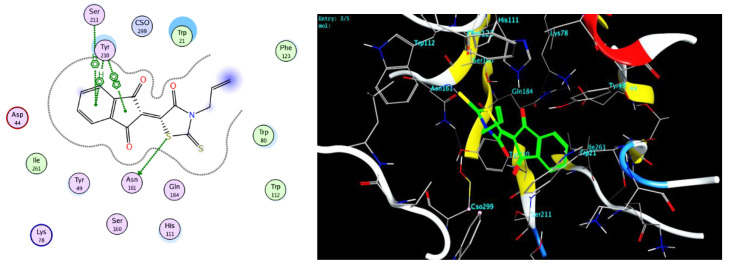
2D and 3D docking of compound **5d** with AKR1B10.

Compound **5d** modeling configuration with AKR1B1 shows hydrogen bonding interaction with ASN 161 and hydrophobic interactions with SER 211 and TYR 210 (Figure 6).

**Figure 7:**
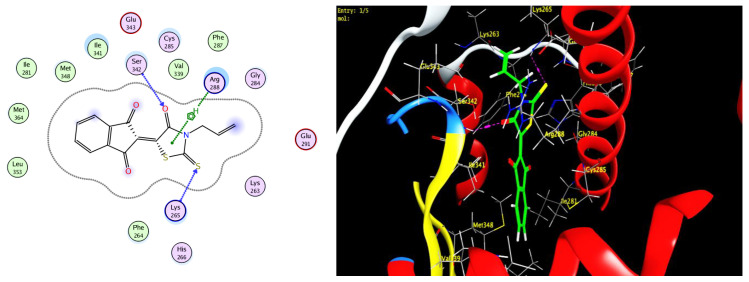
2D and 3D docking of compound **5d** with PPAR*γ*.

Docking data for compound **5d** with PPAR*γ* showed that both the carbonyl-oxygen and the sulfur were hydrogen-bonded with SER 342 and LYS 265, respectively, and hydrophobic interaction with ARG 288 (Figure 7).

**Figure 8:**
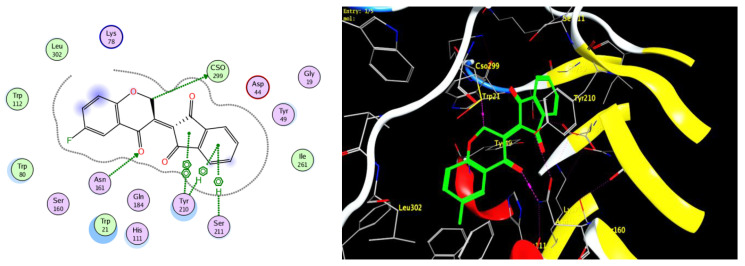
2D and 3D docking of compound **12b** with AKR1B10.

On the other hand, **12b** modeling with AKR1B10 displayed a hydrogen bond to ASN 161 and hydrophobic interaction with TYR 219 and SER 211 (Figure 8).

**Figure 9:**
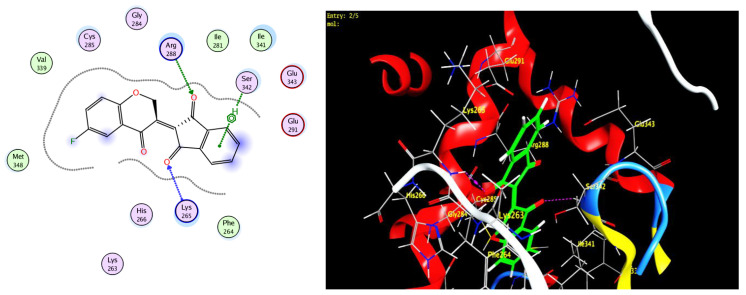
2D and 3D docking of compound **12b** with PPAR*γ*.

In addition, **12b** binds to PPAR*γ* with hydrogen bonds with LYS 265 and ARG 288 and hydrophobic interaction with SER 342 (Figure 9).

**Figure 10:**
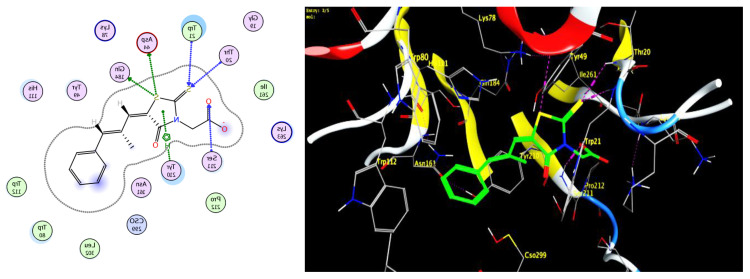
2D and 3D docking of compound Epalrestat with AKR1B10.

The modeling for Epalrestat in AKR1B10 illustrates the carbonyl oxygen and two sulfur groups form hydrogen bonds with SER 211, GLN 184, ASP 44, THR 20, and TRP 21 and hydrophobic interaction with TYR 210 (Figure 10).

**Figure 11:**
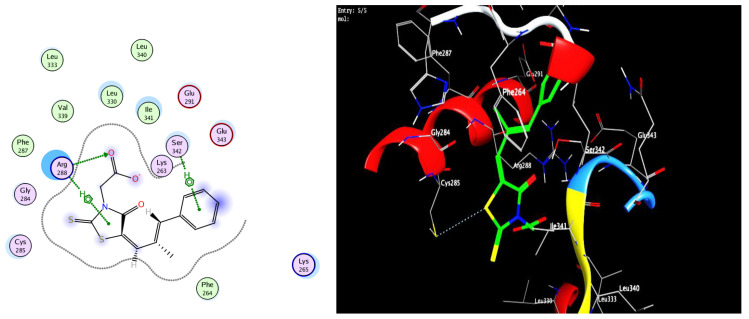
2D and 3D docking of compound Epalrestat with PPAR*γ*.

Moreover, Epalrestat modeling in PPAR*γ* showed that the carbonyl oxygen is hydrogen-bonded to ARG 288 and the hydrophobic interaction with SER 342 and ARG 288 (Figure 11).

## 4. Conclusions

An eco-friendly, promising, and recyclable ionic liquid was synthesized for the first time from pyridine and 2-chloro-1,3-propanediol using sodium acetate as an ion exchanger. Under ultrasonic irradiation, the designed ionic liquid was employed as a promoter for DABCO-catalyzed Knoevenagel reactions in water. Under these conditions, a new series of Knoevenagel products were obtained in excellent yields. This approach offers significant features including gram-scale practicality, superior environmental criteria, simplicity of work-up, and facile recycling of ionic liquid, making it a sustainable and green approach for synthesizing multifunctional Knoevenagel products. The antiproliferative potencies of the newly obtained compounds were evaluated against three tumor cells viz Colo-205, MCF-7, and A549. The results demonstrate that all of the evaluated compounds displayed various activities for the cancer cell lines studied. Furthermore, SAR assessment showed that compounds containing rhodanine (**5**) or chromane (**12**) motifs are necessary for improving the cytotoxic activities. Of them, compound **5c** was three times more potent than Doxorubicin, indicating that it could serve as a promising and new candidate for further research. The newly synthesized compounds are presently being studied in our lab as potential precursors to design interesting spiro compounds.

## Figures and Tables

**Table 1:** Optimization of the reaction conditions for the assembly of 5a.

Entry	Composite System	Equivalent	Conditions	Time (min)	Temp. (°C)	Yield (%)
1.	IL-H_2_O	50%:50%	US ^a^	60	50	62
2.	H_2_O	-	US	60	50	NR
3.	IL-H_2_O-DABCO	50%:50%:1.0 mmol	US	8	30	93
4.	IL-H_2_O-Piperidine	50%:50%:1.0 mmol	US	60	30	75
5.	IL-H_2_O-TEA	50%:50%:1.0 mmol	US	60	30	78
6.	IL-H_2_O-HMTA	50%:50%:1.0 mmol	US	60	30	83
7.	IL-H_2_O-K_2_CO_3_	50%:50%:1.0 mmol	US	60	30	68
8.	IL-H_2_O-KOH	50%:50%:1.0 mmol	US	60	30	56
9.	H_2_O-DABCO	3 mL:1.0 mmol	US	60	30	81
10.	IL-EtOH-DABCO	50%:50%:1.0 mmol	US	60	30	77
11.	IL-DCM-DABCO	50%:50%:1.0 mmol	US	60	30	35
12.	IL-MeCN-DABCO	50%:50%:1.0 mmol	US	60	30	38
13.	IL-H_2_O-DABCO	50%:50%:1.0 mmol	US	25	23	87
14.	IL-H_2_O-DABCO	50%:50%:1.0 mmol	US	15	40	88
15.	IL-H_2_O-DABCO	50%:50%:1.0 mmol	US	25	60	90
16.	IL-H_2_O-DABCO	50%:50%:1.0 mmol	US	25	80	90
17.	IL-H_2_O-DABCO	60%:40%:1.0 mmol	US	5	30	99
18.	IL-H_2_O-DABCO	40%:60%:1.0 mmol	US	10	30	89
19.	IL-H_2_O-DABCO	70%:30%:1.0 mmol	US	5	30	99
20.	IL-H_2_O-DABCO	60%:40%:1.0 mmol	Stirring	60	80	83

^a^ = Ultrasound (US).

**Table 2:** Knoevenagel condensation of isatins with rhodanines under different conditions.

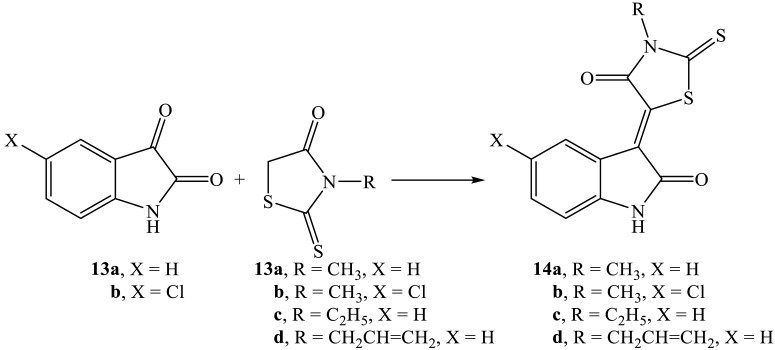						
Entry	Derivatives	Conditions	Time (min)	Yield (%)	Yield Economy (%)	Ref.
1	**14a**	MW/160 °C	34	65	0.52	[51]
2	**14b**	MW/160 °C	34	80	2.35	[51]
3	**14c**	EtOH/Ref.	300	75	0.25	[52]
4	**14d**	MeOH/KOH	20	91	4.55	[53]
5	**14a**	[Py-2OH]AcO	5	99	19.8	Current work
6	**14b**		5	99	19.8	
7	**14c**		6	98	16.3	
8	**14d**		7	99	14.1	

**Table 3:** Knoevenagel condensation of acenaphthylene-1,2-dione with rhodanines under different conditions.

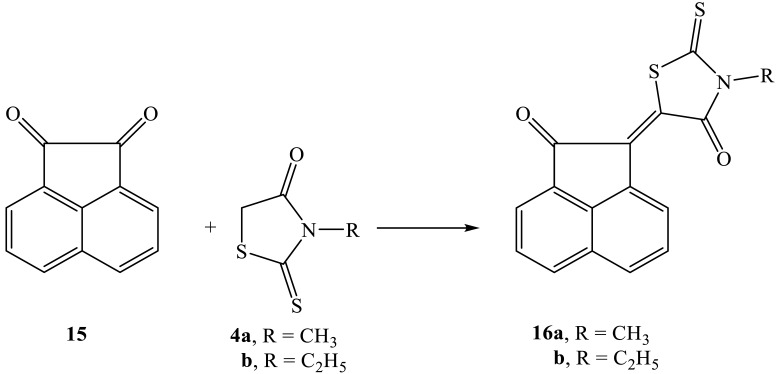						
Entry	Derivatives	Conditions	Time (min)	Yield (%)	Yield Economy (%)	Ref.
1	**16a**	EtOH/Ref.	300	80	0.26	[54]
2	**16b**	EtOH/Ref.	480	74	0.15	[52]
3	**16a**	[Py-2OH]AcO	5	98	19.60	Current work
4	**16b**		6	98	16.33	

**Table 4:** The in vitro activity of derivatives 5, 8, 10, and 12 (expressed as IC_50_ (μM)) against MCF-7, A549, and Colo-205 human cancer cell lines.

Entry	Compounds	IC_50_ (μM) ^a^		
		MCF-7	A549	Colo-205
1.	**5a**	3.9 ± 2.18	3.6 ± 3.54	2.9 ± 3.36
2.	**5b**	2.61 ± 1.22	3.71 ± 2.13	3.11 ± 1.35
3.	**5c**	0.06 ± 0.01	0.04 ± 0.01	0.05 ± 0.02
4.	**5d**	0.21 ± 0.02	0.19 ± 0.03	0.15 ± 0.01
5.	**8a**	2.31 ± 1.01	2.36 ± 0.69	2.38 ± 1.95
6.	**8b**	4.36 ± 1.59	5.13 ± 5.38	5.51 ± 3.11
7.	**8c**	2.18 ± 1.56	1.11 ± 1.27	0.89 ± 0.17
8.	**8d**	3.13 ± 0.97	3.22 ± 0.25	4.08 ± 1.67
9.	**10a**	11.21 ± 2.42	10.16 ± 1.62	6.55 ± 2.92
10.	**10b**	9.15 ± 4.24	11.81 ± 7.92	7.14 ± 5.32
11.	**12a**	1.33 ± 0.53	1.31 ± 1.75	1.94 ± 1.21
12.	**12b**	0.12 ± 0.01	0.11 ± 0.02	0.14 ± 0.04
13.	Doxorubicin	0.19 ± 0.02	0.12 ± 0.01	0.18 ± 0.11

^a^ IC_50_ = mean ± SD of three separate experiments.

**Table 5:** Binding score of compounds **5c,d, 12b** to AKR1B10 and PPAR*γ*.

Compound	Binding Score ΔK (KJ/mole)	
	AKR1B10	PPAR*γ*
**5c**	−7.11	−6.38
**5d**	−6.93	−6.62
**12b**	−6.40	−6.42
Epalrestat	−7.40	−6.57

## Data Availability

Data are contained within the article.

## Supplementary Materials

The following supporting information can be downloaded at: https://www.mdpi.com/article/10.3390/molecules27092940/s1, Copies of NMR and HRMS of the synthesized compounds.

## References

[1] Mendgen T., Steuer C., Klein C. (2012). Privileged Scaffolds or Promiscuous Binders: A Comparative Study on Rhodanines and Related Heterocycles in Medicinal Chemistry. J. Med. Chem..

[2] Ramirez M., Borja N. (2008). Epalrestat: An Aldose Reductase Inhibitor for the Treatment of Diabetic Neuropathy. Pharmacotherapy.

[3] Enchev V., Chorbadjiev S., Jordanov B. (2002). Comparative Study of the Structure of Rhodanine, Isorhodanine, Thiazolidine-2,4-dione, and Thiorhodanine. Chem. Heterocycl. Compd..

[4] Krátky’ M., Štěpánková S., Vorčáková K., Vinšová J. (2016). Synthesis and in vitro evaluation of novel rhodanine derivatives as potential cholinesterase inhibitors. Bioorg. Chem..

[5] Afifi O.S., Shaaban O.G., Abd El Razik H.A., Shams El-Dine A., Ashour F.A., El-Tombary A.A., Abu-Serie M.M. (2019). Synthesis and biological evaluation of purine-pyrazole hybrids incorporating thiazole, thiazolidinone or rhodanine moiety as 15-LOX inhibitors endowed with anticancer and antioxidant potential. Bioorg. Chem..

[6] El-Miligy M.M.M., Hazzaa A.A., El-Messmary H., Nassra R.A., El-Hawash S.A.M. (2017). New hybrid molecules combining benzothiophene or benzofuran with rhodanine as dual COX-1/2 and 5-LOX inhibitors: Synthesis, biological evaluation and docking study. Bioorg. Chem..

[7] Yang N., Ren Z., Zheng J., Feng L., Li D., Gao K., Zhang L., Liu Y., Zuo P. (2016). 5-(4-hydroxy-3-dimethoxybenzylidene)-rhodanine (RD-1)-improved mitochondrial function prevents anxiety- and depressive-like states induced by chronic corticosterone injections in mice. Neuropharmacology.

[8] Song H., Lee Y.S., Roh E.J., Seo J.H., Oh K.-S., Lee B.H., Han H., Shin K.J. (2012). Discovery of potent and selective rhodanine type IKKβ inhibitors by hit-to-lead strategy. Bioorg. Med. Chem. Lett..

[9] Heng S., Tieu W., Hautmann S., Kuan K., Pedersen D.S., Pietsch M., Gütschow M., Abell A.D. (2011). New cholesterol esterase inhibitors based on rhodanine and thiazolidinedione scaffolds. Bioorg. Med. Chem..

[10] Liu J., Wu F., Chen L., Hu J., Zhao L., Chen C., Peng L. (2011). Evaluation of dihydropyrimidin-(2H)-one analogues and rhodanine derivatives as tyrosinase inhibitors. Bioorg. Med. Chem. Lett..

[11] Chandrappa S., Chandru H., Sharada A.C., Vinaya K., Kumar C.A., Thimmegowda N.R., Nagegowda P., Kumar M.K., Rangappa K.S. (2010). Synthesis and in vivo anticancer and antiangiogenic effects of novel thioxothiazolidin-4-one derivatives against transplantable mouse tumor. Med. Chem. Res..

[12] Chauhan D., George G., Sridhar S.N.C., Bhatia R., Paul A.T., Monga V. (2019). Design, synthesis, biological evaluation, and molecular modeling studies of rhodanine derivatives as pancreatic lipase inhibitors. Arch. Pharm..

[13] Toumi A., Boudriga S., Hamden K., Sobeh M., Cheurfa M., Askri M., Knorr M., Strohmann C., Brieger L. (2021). Synthesis, antidiabetic activity and molecular docking study of rhodanine-substitued spirooxindole pyrrolidine derivatives as novel α-amylase inhibitors. Bioorg. Chem..

[14] Celestina S.K., Sundaram K., Ravi S. (2020). In vitro studies of potent aldose reductase inhibitors: Synthesis, characterization, biological evaluation and docking analysis of rhodanine-3-hippuric acid derivatives. Bioorg. Chem..

[15] Tintori C., Iovenitti G., Ceresola E.R., Ferrarese R., Zamperini C., Brai A., Poli G., Dreassi E., Cagno V., Lembo D. (2018). Rhodanine derivatives as potent anti-HIV and anti-HSV microbicides. PLoS ONE.

[16] Nitsche C., Schreier V.N., Behnam M.A., Kumar A., Bartenschlager R., Klein C.D. (2013). Thiazolidinone–Peptide Hybrids as Dengue Virus Protease Inhibitors with Antiviral Activity in Cell Culture. J. Med. Chem..

[17] Maccari R., Ottanà R. (2015). Targeting Aldose Reductase for the Treatment of Diabetes Complications and Inflammatory Diseases: New Insights and Future Directions. J. Med. Chem..

[18] Tammali R., Ramana K.V., Singhal S.S., Awasthi S., Srivastava S.K. (2006). Aldose Reductase Regulates Growth Factor-Induced Cyclooxygenase-2 Expression and Prostaglandin E2 Production in Human Colon Cancer Cells. Cancer Res..

[19] Balendiran G.K., Martin H.J., El-Hawari Y., Maser E. (2009). Cancer biomarker AKR1B10 and carbonyl metabolism. Chem.-Biol. Interact..

[20] Yki-Jarvinen H. (2004). Thiazolidinediones. N. Engl. J. Med..

[21] Eucker J., Bängeroth K., Zavrski I., Krebbel H., Zang C., Heider U., Jakob C., Elstner E., Possinger K., Sezer O. (2004). Ligands of peroxisome proliferator-activated receptor γ induce apoptosis in multiple myeloma. Anti-Cancer Drugs.

[22] Mueller E., Smith M., Sarraf P., Kroll T., Aiyer A., Kaufman D.S., Oh W., Demetri G., Figg W.D., Zhou X.P. (2000). Effects of ligand activation of peroxisome proliferator-activated receptor γ in human prostate cancer. PNAS.

[23] Priede E., Brica S., Bakis E., Udris N., Zicmanis A. (2015). Ionic liquids as solvents for the Knoevenagel condensation: Understanding the role of solvent–solute interactions. New J. Chem..

[24] Yamazaki S., Katayama K., Wang Z., Mikata Y., Morimoto T., Ogawa A. (2021). Sequential Knoevenagel Condensation/Cyclization for the Synthesis of Indene and Benzofulvene Derivatives. ACS Omega.

[25] Appaturi J.N., Ratti R., Phoon B.L., Batagarawa S.M., Din I.U., Selvaraj M., Ramalingam R.J. (2021). A review of the recent progress on heterogeneous catalysts for Knoevenagel condensation. Dalton Trans..

[26] van Beurdena K., de Koning S., Molendijk D., van Schijndel J. (2020). The Knoevenagel reaction: A review of the unfinished treasure map to forming carbon–carbon bonds. Green Chem. Lett. Rev..

[27] Keller T.C., Isabettini S., Verboekend D., Rodrigues E.G., P´erez-Ram´ırez J. (2014). Hierarchical high-silica zeolites as superior base catalysts. Chem. Sci..

[28] Karmakar A., Paul A., Mahmudov K.T., da Silva M.F.C.G., Pombeiro A.J.L. (2016). pH dependent synthesis of Zn(ii) and Cd(ii) coordination polymers with dicarboxyl-functionalized arylhydrazone of barbituric acid: Photoluminescence properties and catalysts for Knoevenagel condensation. New J. Chem..

[29] Li G., Xiao J., Zhang W. (2012). Efficient and reusable amine-functionalized polyacrylonitrile fiber catalysts for Knoevenagel condensation in water. Green Chem..

[30] Zhang F., Jiang H., Li X., Wu X., Li H. (2014). Amine-Functionalized GO as an Active and Reusable Acid–Base Bifunctional Catalyst for One-Pot Cascade Reactions. ACS Catal..

[31] Panja S.K., Dwivedi N., Saha S. (2015). First report of the application of simple molecular complexes as organo-catalysts for Knoevenagel condensation. RSC Adv..

[32] Garrabou X., Wicky B.I.M., Hilvert D. (2016). Fast Knoevenagel Condensations Catalyzed by an Artificial Schiff-Base-Forming Enzyme. J. Am. Chem. Soc..

[33] Ying A., Qiu F., Wu C., Hu H., Yang J. (2014). Ionic tagged amine supported on magnetic nanoparticles: Synthesis and application for versatile catalytic Knoevenagel condensation in water. RSC Adv..

[34] Xi F.-G., Liu H., Yang N.-N., Gao E.-O. (2016). Aldehyde-Tagged Zirconium Metal–Organic Frameworks: A Versatile Platform for Postsynthetic Modification. Inorg. Chem..

[35] Meng D., Qiao Y., Wang X., Wen W., Zhao S. (2018). DABCO-catalyzed Knoevenagel condensation of aldehydes with ethyl cyanoacetate using hydroxy ionic liquid as a promoter. RSC Adv..

[36] Pandolfi F., Feroci M., Chiarotto I. (2018). Role of Anion and Cation in the 1-Methyl-3-butyl Imidazolium Ionic Liquids BMImX: The Knoevenagel Condensation. ChemistrySelect.

[37] Goodman E.D., Zhou C., Cargnello M. (2020). Design of Organic/Inorganic Hybrid Catalysts for Energy and Environmental Applications. ACS Cent. Sci..

[38] Sadjadi S. (2021). Magnetic (poly) ionic liquids: A promising platform for green chemistry. J. Mol. Liq..

[39] Polshettiwar V., Varma R.S.S. (2010). Green chemistry by nano-catalysis. Green Chem..

[40] Adam J.G., Johan J., Christopher H. (2020). Industrial Applications of Ionic Liquids. Molecules.

[41] Sandip K.S., Anthony W.S. (2020). Ionic liquids synthesis and applications: An overview. J. Mol. Liq..

[42] Wang B., Qin L., Mu T., Xue Z., Gao G. (2017). Are Ionic Liquids Chemically Stable?. Chem. Rev..

[43] Arafa W.A.A. (2018). An eco-compatible pathway to the synthesis of mono and bis-multisubstituted imidazoles over novel reusable ionic liquids: An efficient and green sonochemical process. RSC Adv..

[44] Buettner C.S., Cognigni A., Schröder C., Bica-Schröder K. (2022). Surface-active ionic liquids: A review. J. Mol. Liq..

[45] Khaligh N.G., Mihankhah T., Johan M.R. (2019). Synthesis of new low-viscous sulfonic acid-functionalized ionic liquid and its application as a Brönsted liquid acid catalyst for the one-pot mechanosynthesis of 4H-pyrans through the ball milling process. J. Mol. Liq..

[46] Dalessandro E.V., Collin H.P., Guimarães L.G.L., Valle M.S., Pliego J.R. (2017). Mechanism of the Piperidine-Catalyzed Knoevenagel Condensation Reaction in Methanol: The Role of Iminium and Enolate Ions. J. Phys. Chem. B.

[47] Zhao S., He M., Guo Z., Zhou N., Wang D., Li J., Zhang L. (2015). [HyEtPy]Cl–H2O: An efficient and versatile solvent system for the DABCO-catalyzed Morita–Baylis–Hillman reaction. RSC Adv..

[48] Arafa W.A.A. (2019). Deep eutectic solvent for an expeditious sono-synthesis of novel series of bis-quinazolin-4-one derivatives as potential anti-cancer agents. R. Soc. Open Sci..

[49] Arafa W.A.A., Mourad A.K. (2019). New dicationic DABCO-based ionic liquids: A scalable metal-free one-pot synthesis of bis-2-amino-5-arylidenethiazol-4-ones. R. Soc. Open Sci..

[50] Pagadala R., Kasi V., Shabalala N.G., Jonnalagadda S.B. (2022). Ultrasound-assisted multicomponent synthesis of heterocycles in water—A review. Arabian J. Chem..

[51] Swatloski R.P., Holbrey J.D., Rogers R.D. (2003). Ionic liquids are not always green: Hydrolysis of 1-butyl-3-methylimidazolium hexafluorophosphate. Green Chem..

[52] Freire M.G., Neves C.M.S.S., Marrucho I.M., Coutinho J.A.P., Fernandes A.M. (2010). Hydrolysis of Tetrafluoroborate and Hexafluorophosphate Counter Ions in Imidazolium-Based Ionic Liquids. J. Phys. Chem. A.

[53] Arafa W.A.A., Mohamed A.M., Abdel-Magied A.F. (2017). Ultrasound-Mediated Three-Component Reaction on-Water Protocol for the Synthesis of Novel Mono- and Bis-1,3-Thiazin-4-One Derivatives. Heterocycles.

[54] Safer A., Rahmouni M., Carreaux F., Paquin L., Lozach O., Meijer L., Bazureau J.P. (2011). An expeditious, environment-friendly, and microwave-assisted synthesis of 5-isatinylidenerhodanine derivatives. Chem. Pap..

[55] Pardasani R.T., Pardasani P., Jain A., Kohli S. (2004). Synthesis and Semiempirical Calculations of Thiazolidinone and Imidazolidinone Derivatives of A-Diones. Phosphorus Sulfur Silicon Relat. Elem..

[56] Shvets A.A., Kurbatov S.V. (2012). Synthesis of bis-β,β′-spiro-pyrrolidinyl-oxindoles, containing a rhodanine fragment. Chem. Heterocycl. Compd..

[57] Pardasani R.T., Pardasani P., Sharma I., Saxena A., Kohli S. (2003). Synthesis and semiempirical calculations of imidazolidine, isoxazolone and thiazoline thiol derivatives of acenaphthylene-1,2-dione. Indian J. Chem..

[58] Sheldon R.A. (2018). Metrics of Green Chemistry and Sustainability: Past, Present, and Future. ACS Sustain. Chem. Eng..

[59] Arora S., Joshi G., Kalra S., Wani A.A., Bharatam P.V., Kumar P., Kumar R. (2019). Knoevenagel/Tandem Knoevenagel and Michael Adducts of Cyclohexane-1,3-dione and Aryl Aldehydes: Synthesis, DFT Studies, Xanthine Oxidase Inhibitory Potential, and Molecular Modeling. ACS Omega.

[60] Tsuji M., Murota S., Morita I. (2003). Docosapentaenoic acid (22:5, n-3) suppressed tube forming activity in endothelial cells induced by vascular endothelial growth factor. Prostaglandins Leukot. Essent. Fatty Acids.

[61] Arafa W.A.A., Hussein M.F. (2020). Design, Sonosynthesis, Quantum-Chemical Calculations, and Evaluation of New Mono- and Bis-pyridine Dicarbonitriles as Antiproliferative Agents. Chin. J. Chem..

[62] Shao J.-W., Dai Y.-C., Xue J.-P., Wang J.-C., Lin F.-P., Guo Y.-G. (2011). In vitro and in vivo anticancer activity evaluation of ursolic acid derivatives. Eur. J. Med. Chem..

