# Supplementary material for: Novel Pyridinium Based Ionic Liquid Promoter for Aqueous Knoevenagel Condensation: Green and Efficient Synthesis of New Derivatives with Their Anticancer Evaluation
Source: Molecules. 2022 May 4;27(9):2940. doi: 10.3390/molecules27092940 (PMC9105511; doi:10.3390/molecules27092940)
Supplement: Supplementary file 1 [file molecules-27-02940-s001.zip › molecules-1690579-supplementary.pdf]

Supporting Information

Table of content

No.	Items	Page
**1.**	^{1}H NMR,^{13}C NMR, and HRMS of **2**	S2
**2.**	^{1}H NMR,^{13}C NMR, and HRMS of **5a**	S5
**3.**	^{1}H NMR, and HRMS of **5b**	S8
**4.**	^{1}H NMR, and HRMS of **5c**	S10
**5.**	^{1}H NMR, and HRMS of **5d**	S12
**6.**	HRMS of **6**	S14
**7.**	^{1}H NMR,^{13}C NMR, and HRMS of **8a**	S15
**8.**	^{1}H NMR, and HRMS of **8b**	S18
**9.**	^{1}H NMR, and HRMS of **8c**	S20
**10.**	^{1}H NMR, and HRMS of **2**	S22
**11.**	^{1}H NMR, and HRMS of **10a**	S24
**12.**	^{1}H NMR, and HRMS of **10b**	S26
**13.**	^{1}H NMR, and HRMS of **12a**	S28
**14.**	^{1}H NMR,^{13}C NMR, ^{19}FNMR, and HRMS of **8a**	S30

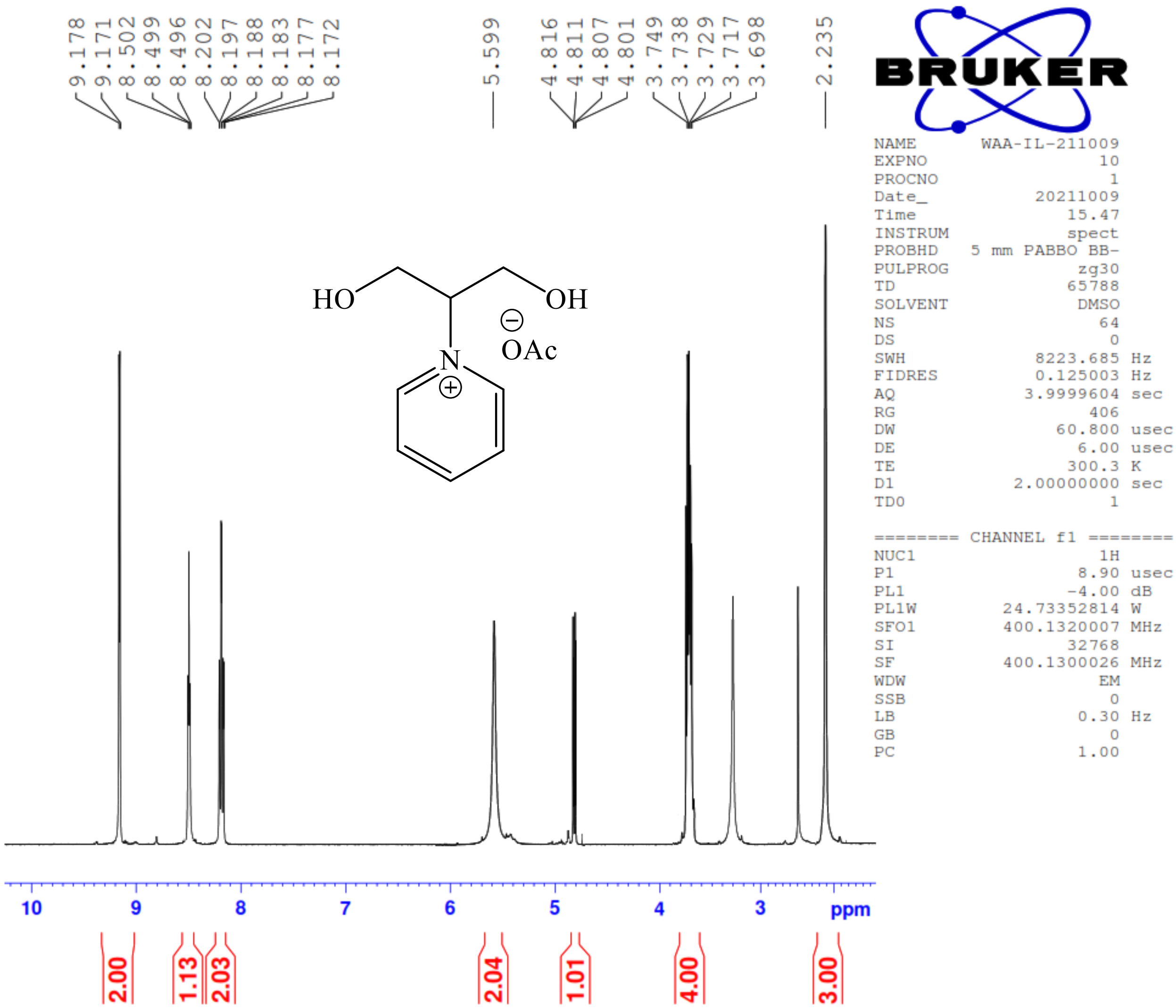
9.178
9.171
8.502
8.499
8.496
8.202
8.197
8.188
8.183
8.177
8.172
5.599
4.816
4.811
4.807
4.801
3.749
3.738
3.729
3.717
3.698
2.235
BRUKER
NAME WAA-IL-211009
EXPNO 10
PROCNO 1
Date_ 20211009
Time 15.47
INSTRUM spect
PROBHD 5 mm PABBO BB-
PULPROG zg30
TD 65788
SOLVENT DMSO
NS 64
DS 0
SWH 8223.685 Hz
FIDRES 0.125003 Hz
AQ 3.9999604 sec
RG 406
DW 60.800 usec
DE 6.00 usec
TE 300.3 K
D1 2.00000000 sec
TD0 1
======== CHANNEL f1 ========
NUC1 1H
P1 8.90 usec
PL1 -4.00 dB
PL1W 24.73352814 W
SFO1 400.1320007 MHz
SI 32768
SF 400.1300026 MHz
WDW EM
SSB 0
LB 0.30 Hz
GB 0
PC 1.00
HO
OH
N
OAc
10
9
8
7
6
5
4
3
ppm
2.00
1.13
2.03
2.04
1.01
4.00
3.00

^{1}H NMR of **2**

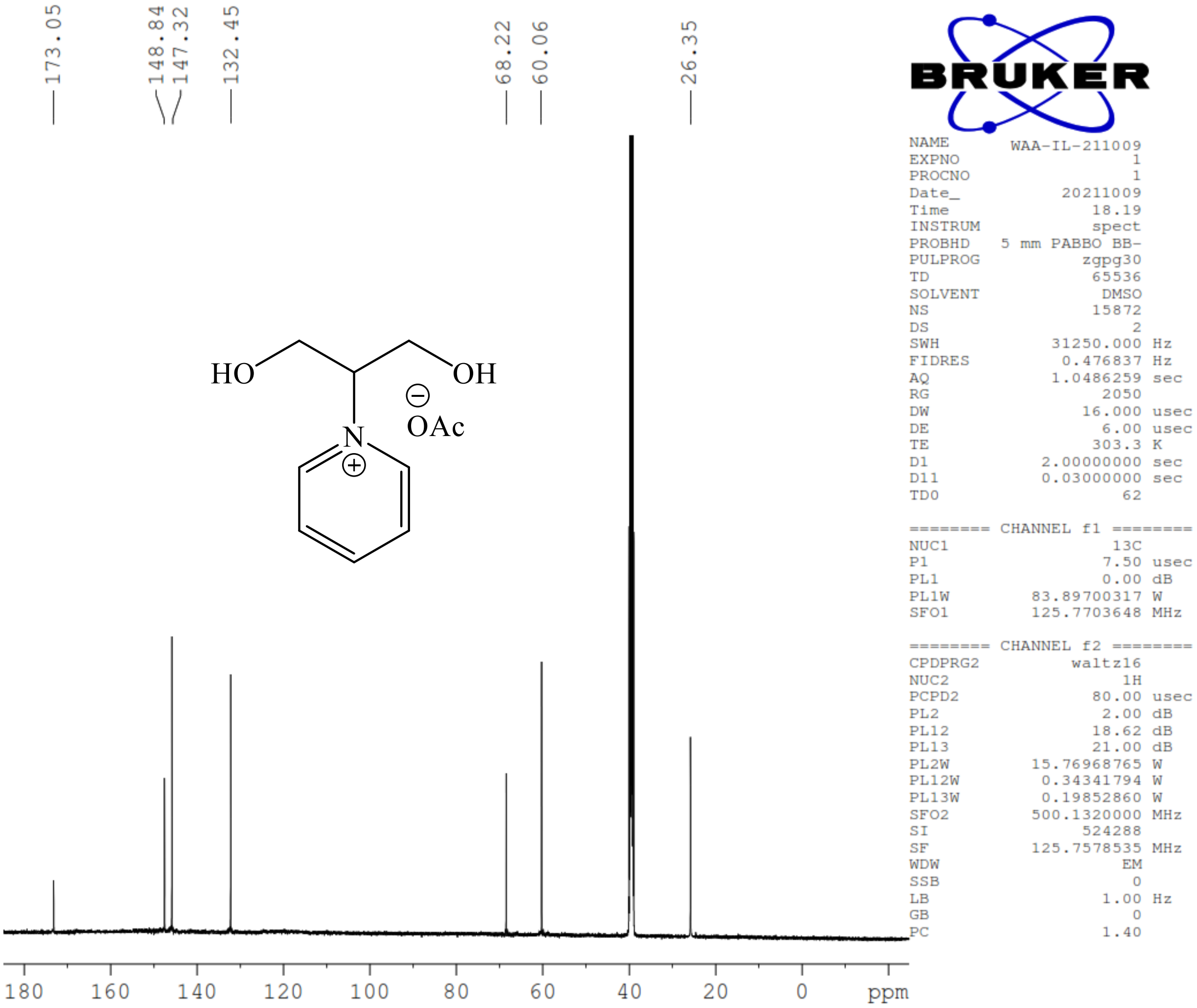
173.05
148.84
147.32
132.45
68.22
60.06
26.35
HO
OH
N
OAc
BRUKER
NAME WAA-IL-211009
EXPNO 1
PROCNO 1
Date_ 20211009
Time 18.19
INSTRUM spect
PROBHD 5 mm PABBO BB-
PULPROG zgpg30
TD 65536
SOLVENT DMSO
NS 15872
DS 2
SWH 31250.000 Hz
FIDRES 0.476837 Hz
AQ 1.0486259 sec
RG 2050
DW 16.000 usec
DE 6.00 usec
TE 303.3 K
D1 2.00000000 sec
D11 0.03000000 sec
TD0 62
======== CHANNEL f1 ========
NUC1 13C
P1 7.50 usec
PL1 0.00 dB
PL1W 83.89700317 W
SFO1 125.7703648 MHz
======== CHANNEL f2 ========
CPDPRG2 waltz16
NUC2 1H
PCPD2 80.00 usec
PL2 2.00 dB
PL12 18.62 dB
PL13 21.00 dB
PL2W 15.76968765 W
PL12W 0.34341794 W
PL13W 0.19852860 W
SFO2 500.1320000 MHz
SI 524288
SF 125.7578535 MHz
WDW EM
SSB 0
LB 1.00 Hz
GB 0
PC 1.40
180 160 140 120 100 80 60 40 20 0 ppm

^{13}C NMR of **2**

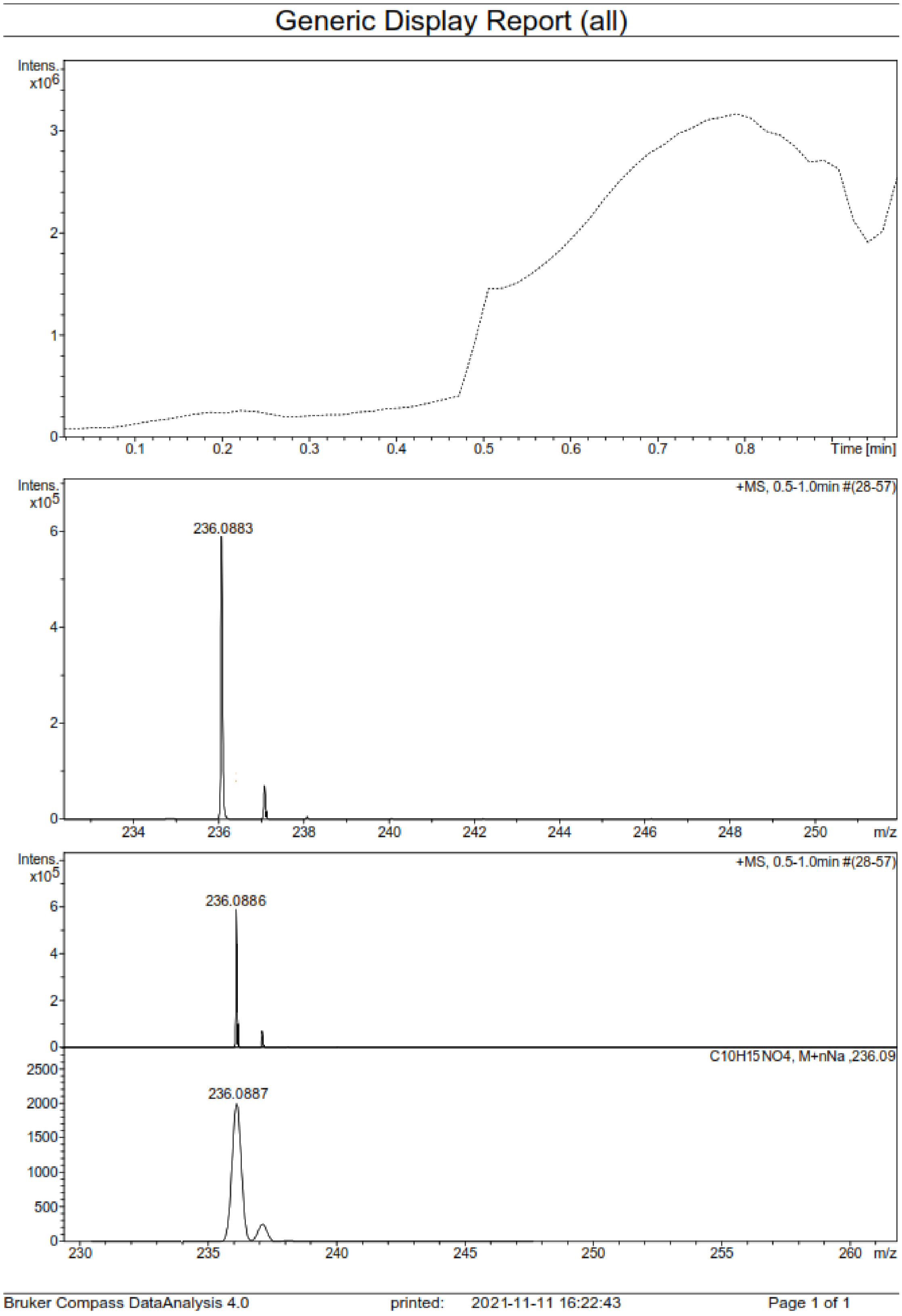

Generic Display Report (all)
Intens. x10^6
Time [min]
Intens. x10^5
+MS, 0.5-1.0min #(28-57)
236.0883
m/z
+MS, 0.5-1.0min #(28-57)
236.0886
C10H15NO4, M+nNa ,236.09
236.0887
Bruker Compass DataAnalysis 4.0
printed: 2021-11-11 16:22:43
Page 1 of 1

HRMS of **2**

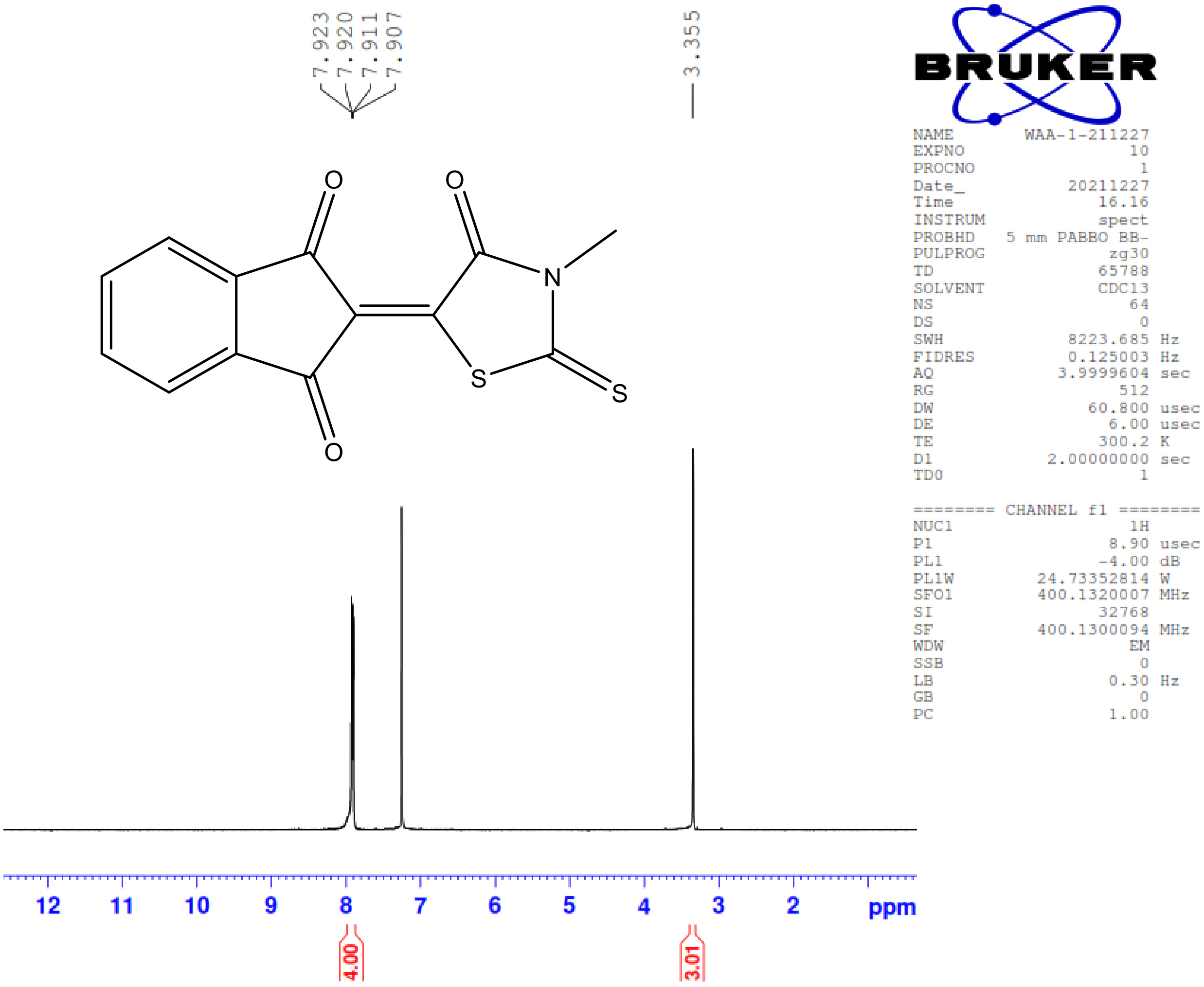
7.923
7.920
7.911
7.907
3.355
O
O
N
S
S
O
NAME WAA-1-211227
EXPNO 10
PROCNO 1
Date_ 20211227
Time 16.16
INSTRUM spect
PROBHD 5 mm PABBO BB-
PULPROG zg30
TD 65788
SOLVENT CDCl3
NS 64
DS 0
SWH 8223.685 Hz
FIDRES 0.125003 Hz
AQ 3.9999604 sec
RG 512
DW 60.800 usec
DE 6.00 usec
TE 300.2 K
D1 2.00000000 sec
TD0 1
======== CHANNEL f1 ========
NUC1 1H
P1 8.90 usec
PL1 -4.00 dB
PL1W 24.73352814 W
SFO1 400.1320007 MHz
SI 32768
SF 400.1300094 MHz
WDW EM
SSB 0
LB 0.30 Hz
GB 0
PC 1.00
12 11 10 9 8 7 6 5 4 3 2 ppm
4.00
3.01

^{1}H NMR of **5a**

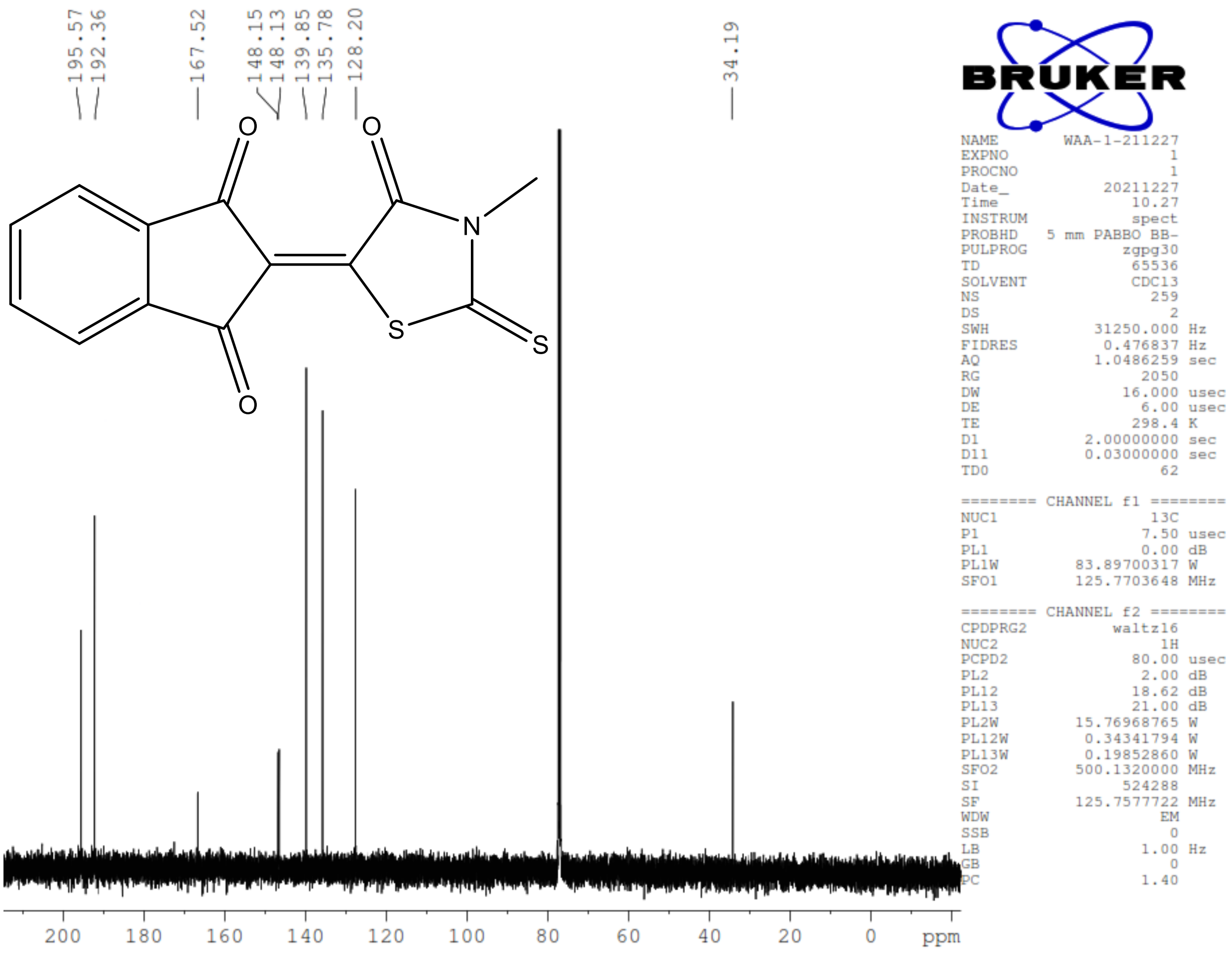
195.57
192.36
167.52
148.15
148.13
139.85
135.78
128.20
34.19
O
O
O
N
S
S
BRUKER
NAME WAA-1-211227
EXPNO 1
PROCNO 1
Date_ 20211227
Time 10.27
INSTRUM spect
PROBHD 5 mm PABBO BB-
PULPROG zgpg30
TD 65536
SOLVENT CDCl3
NS 259
DS 2
SWH 31250.000 Hz
FIDRES 0.476837 Hz
AQ 1.0486259 sec
RG 2050
DW 16.000 usec
DE 6.00 usec
TE 298.4 K
D1 2.00000000 sec
D11 0.03000000 sec
TD0 62
======== CHANNEL f1 ========
NUC1 13C
P1 7.50 usec
PL1 0.00 dB
PL1W 83.89700317 W
SFO1 125.7703648 MHz
======== CHANNEL f2 ========
CPDPRG2 waltz16
NUC2 1H
PCPD2 80.00 usec
PL2 2.00 dB
PL12 18.62 dB
PL13 21.00 dB
PL2W 15.76968765 W
PL12W 0.34341794 W
PL13W 0.19852860 W
SFO2 500.1320000 MHz
SI 524288
SF 125.7577722 MHz
WDW EM
SSB 0
LB 1.00 Hz
GB 0
PC 1.40
200 180 160 140 120 100 80 60 40 20 0 ppm

^{13}C NMR of **5a**

Display Report

Analysis Info

Analysis Name	E:\Data2\wael\1.d	Acquisition Date	2021-12-26 16:09:45
Method	Tune_low_neg.m	Operator	AD
Sample Name	1	Instrument / Ser#	micrOTOF 125
Comment			

Acquisition Parameter

Source Type	ESI	Ion Polarity	Negative	Set Nebulizer	0.3 Bar
Focus	Not active			Set Dry Heater	180 °C
Scan Begin	50 m/z	Set Capillary	4000 V	Set Dry Gas	4.0 l/min
Scan End	1500 m/z	Set End Plate Offset	-500 V	Set Divert Valve	Waste

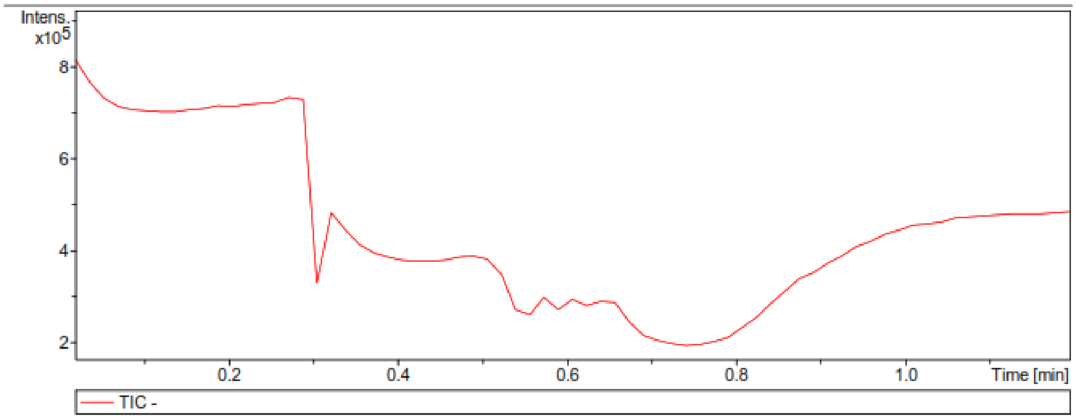

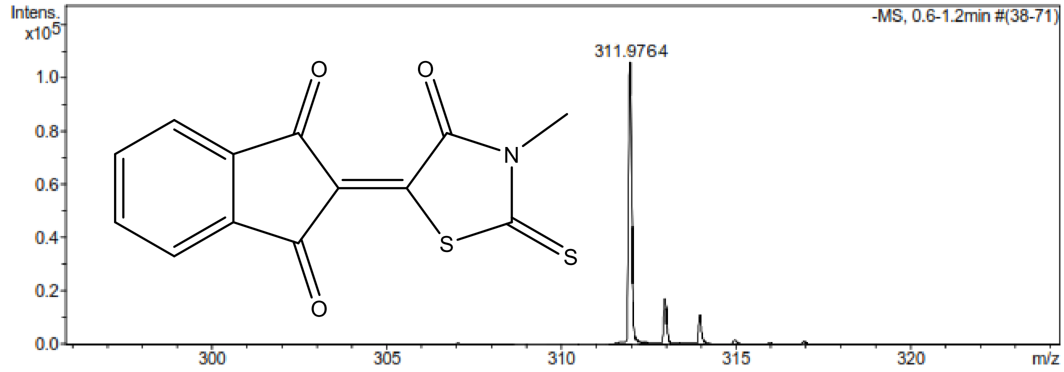

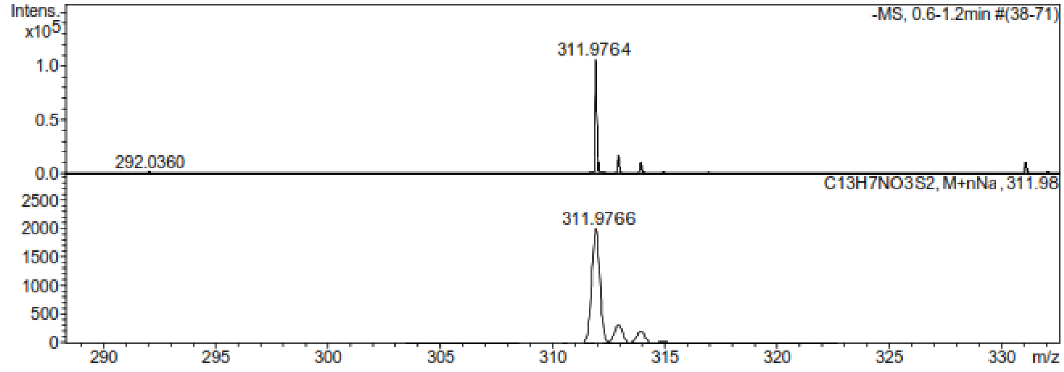

Bruker Compass DataAnalysis 4.0 printed: 2021-12-26 16:53:23 Page 1 of 1

HRMS of **5a**

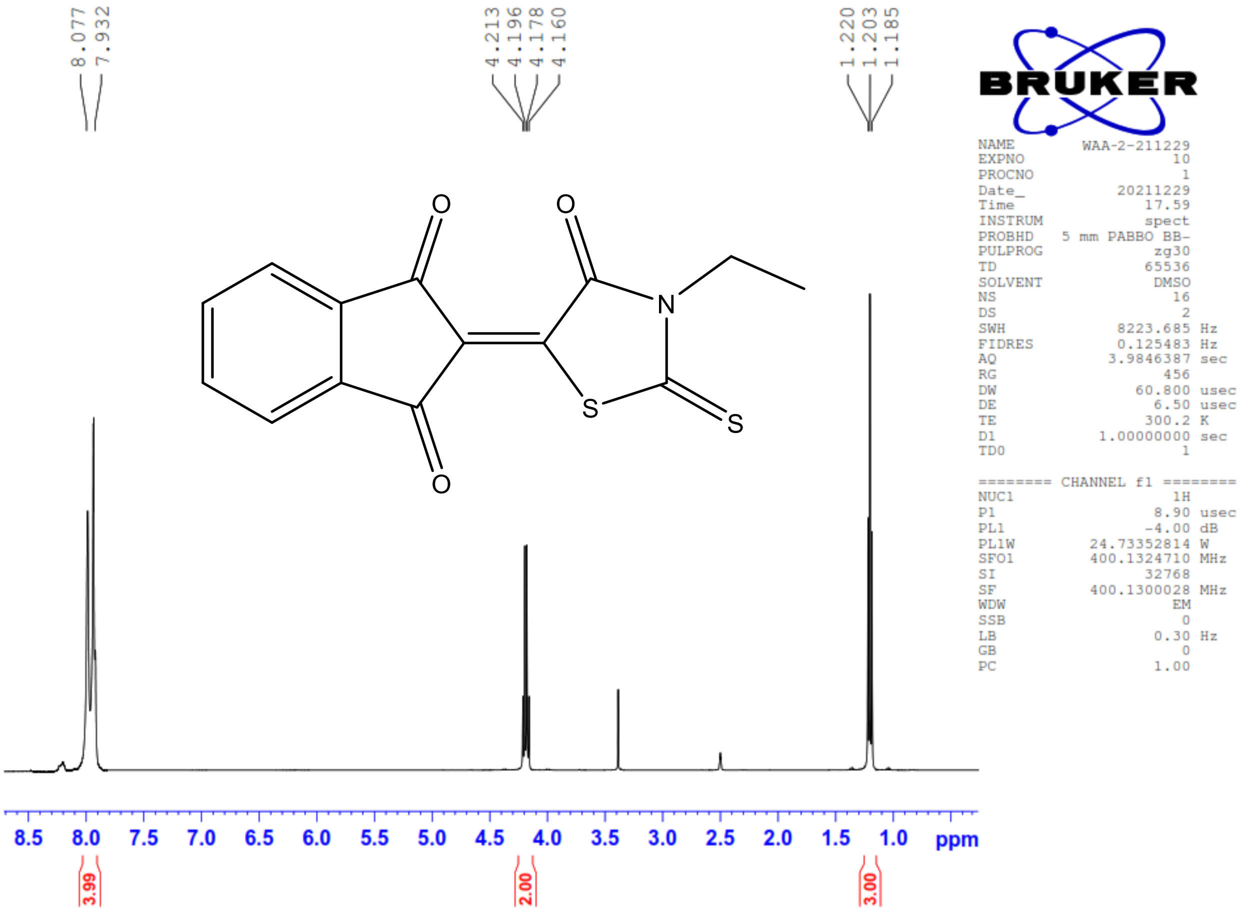

8.077
7.932
4.213
4.196
4.178
4.160
1.220
1.203
1.185
BRUKER
NAME WAA-2-211229
EXPNO 10
PROCNO 1
Date_ 20211229
Time 17.59
INSTRUM spect
PROBHD 5 mm PABBO BB-
PULPROG zg30
TD 65536
SOLVENT DMSO
NS 16
DS 2
SWH 8223.685 Hz
FIDRES 0.125483 Hz
AQ 3.9846387 sec
RG 456
DW 60.800 usec
DE 6.50 usec
TE 300.2 K
D1 1.00000000 sec
TD0 1
======== CHANNEL f1 ========
NUC1 1H
P1 8.90 usec
PL1 -4.00 dB
PL1W 24.73352814 W
SFO1 400.1324710 MHz
SI 32768
SF 400.1300028 MHz
WDW EM
SSB 0
LB 0.30 Hz
GB 0
PC 1.00
O
O
O
N
S
S
8.5 8.0 7.5 7.0 6.5 6.0 5.5 5.0 4.5 4.0 3.5 3.0 2.5 2.0 1.5 1.0 ppm
3.99
2.00
3.00

^{1}H NMR of **5b**

Generic Display Report

Analysis Info

Analysis Name	E:\Data2\arafa\114.d	Acquisition Date	2021-12-25 12:31:54
Method	tune_low_dirk.m	Operator	AD
Sample Name	114	Instrument	micrOTOF
Comment			

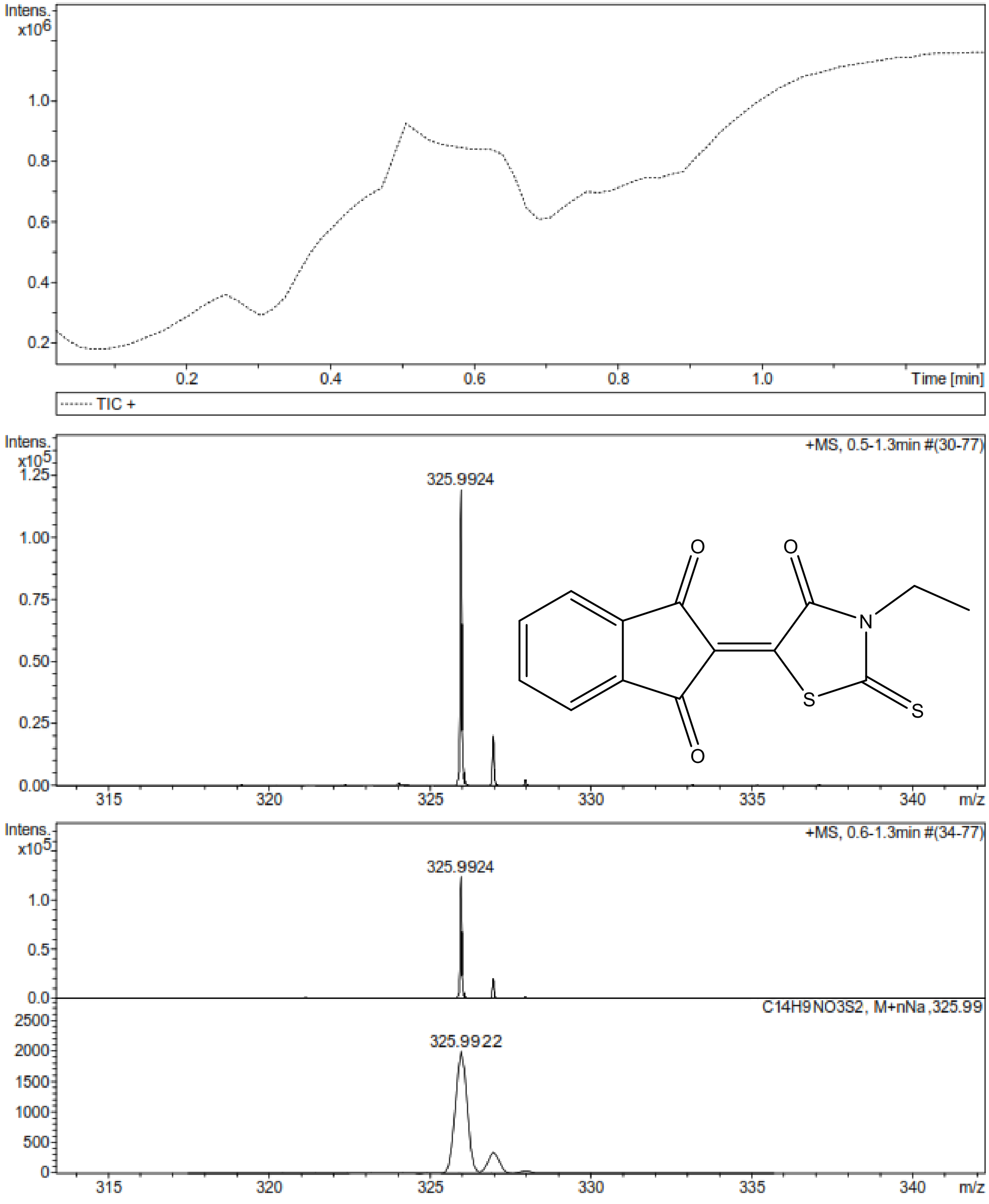

Bruker Compass DataAnalysis 4.0 printed: 2021-12-25 14:25:19 Page 1 of 1

HRMS of **5b**

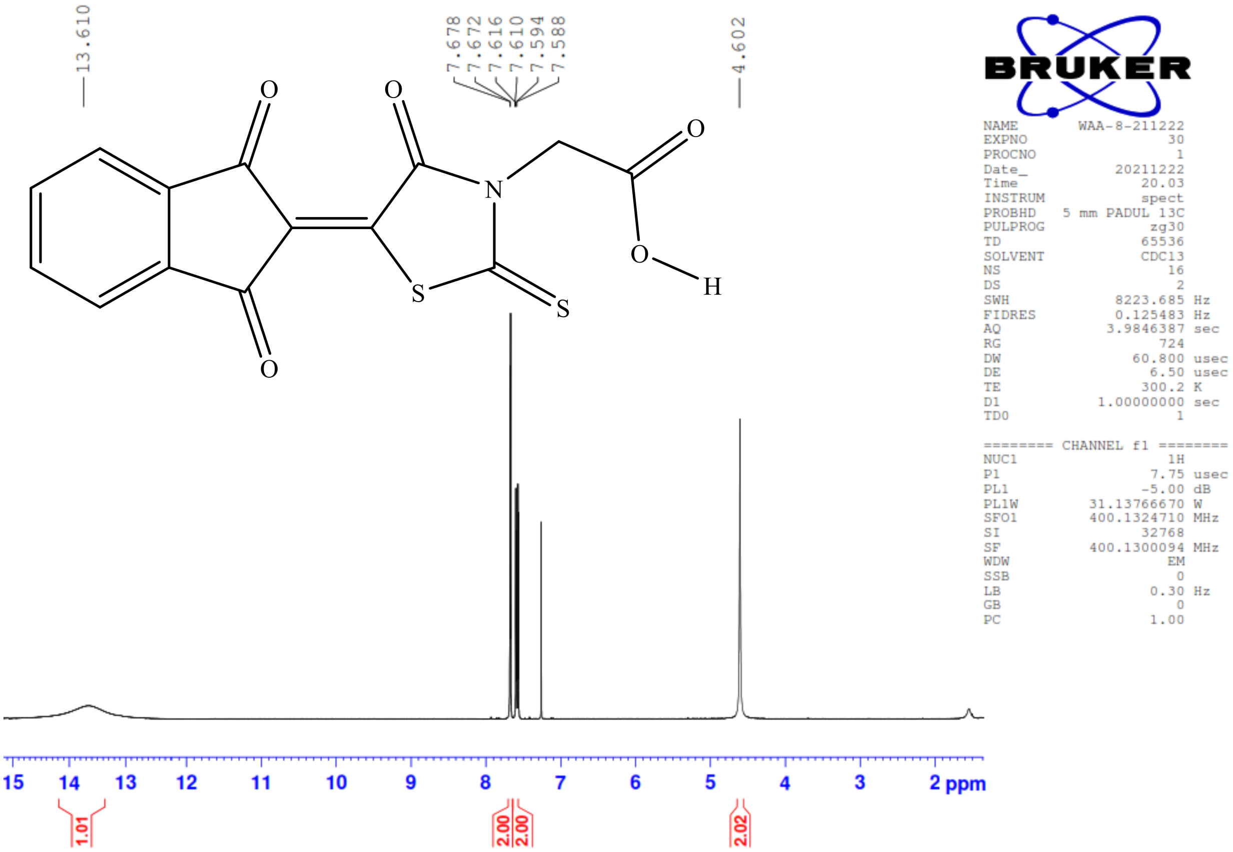
13.610
7.678
7.672
7.616
7.610
7.594
7.588
4.602
NAME WAA-8-211222
EXPNO 30
PROCNO 1
Date_ 20211222
Time 20.03
INSTRUM spect
PROBHD 5 mm PADUL 13C
PULPROG zg30
TD 65536
SOLVENT CDCl3
NS 16
DS 2
SWH 8223.685 Hz
FIDRES 0.125483 Hz
AQ 3.9846387 sec
RG 724
DW 60.800 usec
DE 6.50 usec
TE 300.2 K
D1 1.00000000 sec
TD0 1
======== CHANNEL f1 ========
NUC1 1H
P1 7.75 usec
PL1 -5.00 dB
PL1W 31.13766670 W
SFO1 400.1324710 MHz
SI 32768
SF 400.1300094 MHz
WDW EM
SSB 0
LB 0.30 Hz
GB 0
PC 1.00
15 14 13 12 11 10 9 8 7 6 5 4 3 2 ppm
1.01
2.00
2.00
2.02

Generic Display Report

Analysis Info

Analysis Name	E:\Data2\arafa\113.d	Acquisition Date	2021-12-29 11:13:46
Method	tune_low_dirk.m	Operator	AD
Sample Name	113	Instrument	micrOTOF
Comment			

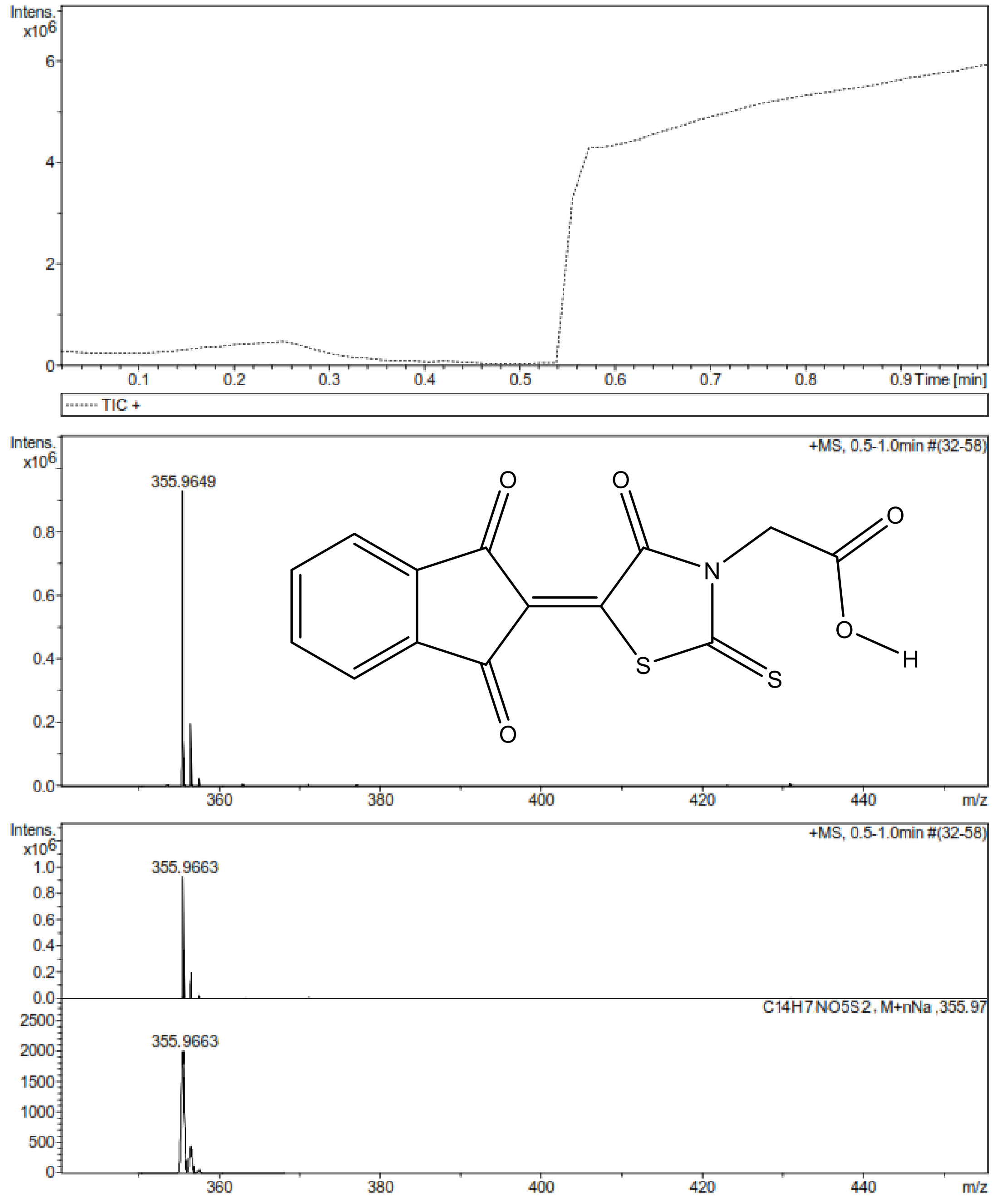

Bruker Compass DataAnalysis 4.0   printed: 2021-12-29 14:22:41   Page 1 of 1

HRMS of **5c**

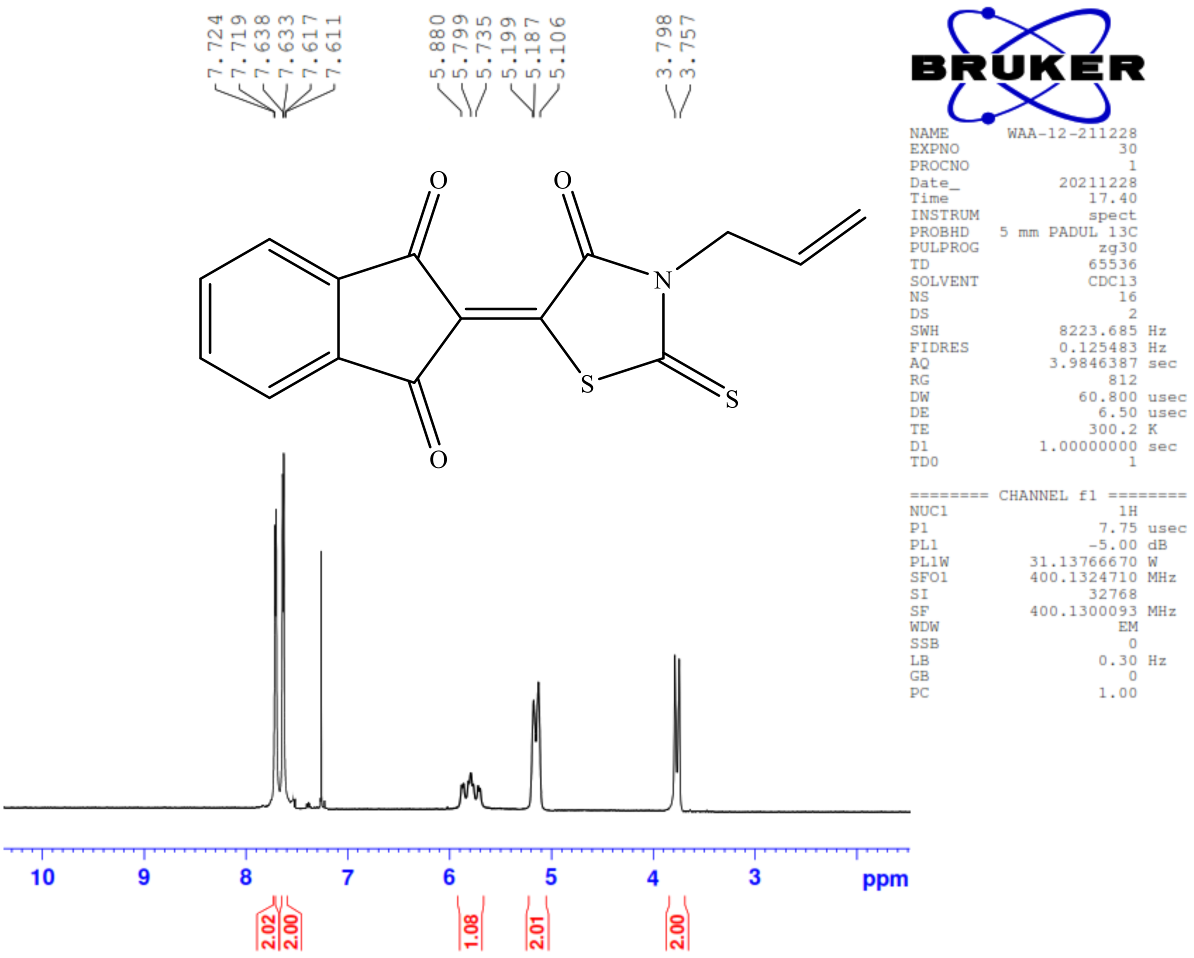
7.724
7.719
7.638
7.633
7.617
7.611
5.880
5.799
5.735
5.199
5.187
5.106
3.798
3.757
BRUKER
NAME WAA-12-211228
EXPNO 30
PROCNO 1
Date_ 20211228
Time 17.40
INSTRUM spect
PROBHD 5 mm PADUL 13C
PULPROG zg30
TD 65536
SOLVENT CDCl3
NS 16
DS 2
SWH 8223.685 Hz
FIDRES 0.125483 Hz
AQ 3.9846387 sec
RG 812
DW 60.800 usec
DE 6.50 usec
TE 300.2 K
D1 1.00000000 sec
TD0 1
======== CHANNEL f1 ========
NUC1 1H
P1 7.75 usec
PL1 -5.00 dB
PL1W 31.13766670 W
SFO1 400.1324710 MHz
SI 32768
SF 400.1300093 MHz
WDW EM
SSB 0
LB 0.30 Hz
GB 0
PC 1.00
ppm
2.02
2.00
1.08
2.01
2.00

^{1}H NMR of **5d**

Display Report

Analysis Info

Analysis Name	E:\Data2\wael\5.d	Acquisition Date	2021-12-26 16:06:48
Method	Tune_low_neg.m	Operator	AD
Sample Name	5	Instrument / Ser#	micrOTOF 125
Comment			

Acquisition Parameter

Source Type	ESI	Ion Polarity	Negative	Set Nebulizer	0.3 Bar
Focus	Not active			Set Dry Heater	180 °C
Scan Begin	50 m/z	Set Capillary	4000 V	Set Dry Gas	4.0 l/min
Scan End	1500 m/z	Set End Plate Offset	-500 V	Set Divert Valve	Waste

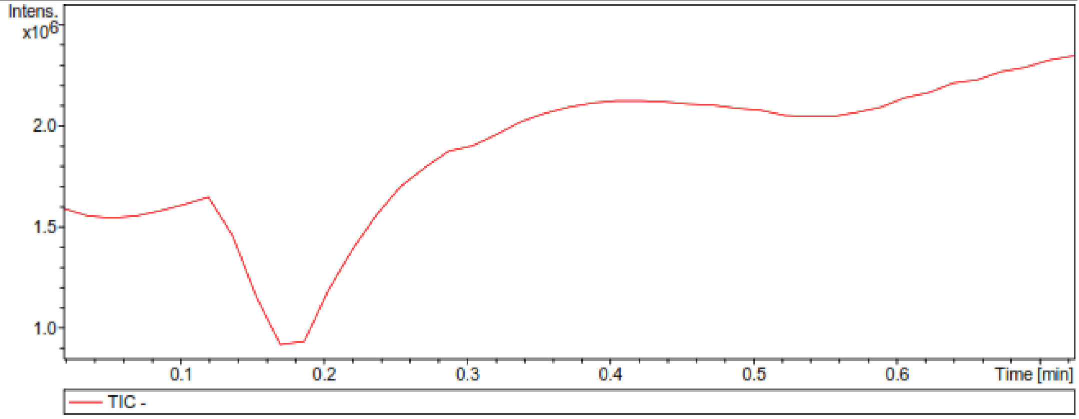

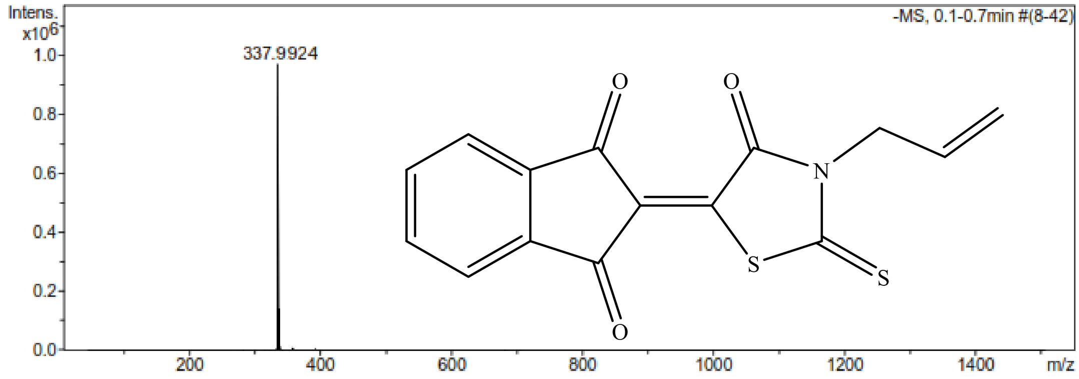

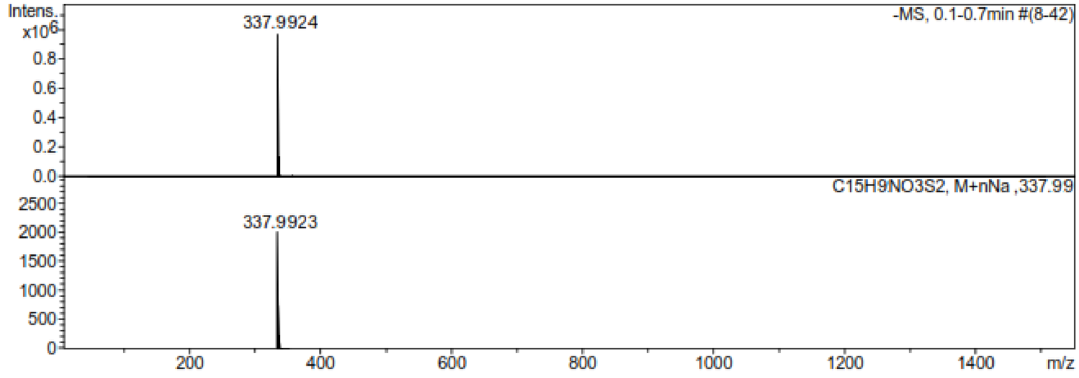

Bruker Compass DataAnalysis 4.0 printed: 2021-12-26 16:55:32 Page 1 of 1

HRMS of **5d**

Display Report

Analysis Info

Analysis Name	E:\Data2\wael\62.d	Acquisition Date	2021-12-06 16:17:04
Method	Tune_low_neg.m	Operator	AD
Sample Name	62	Instrument / Ser#	micrOTOF 125
Comment			

Acquisition Parameter

Source Type	ESI	Ion Polarity	Negative	Set Nebulizer	0.3 Bar
Focus	Not active			Set Dry Heater	180 °C
Scan Begin	50 m/z	Set Capillary	4000 V	Set Dry Gas	4.0 l/min
Scan End	1500 m/z	Set End Plate Offset	-500 V	Set Divert Valve	Waste

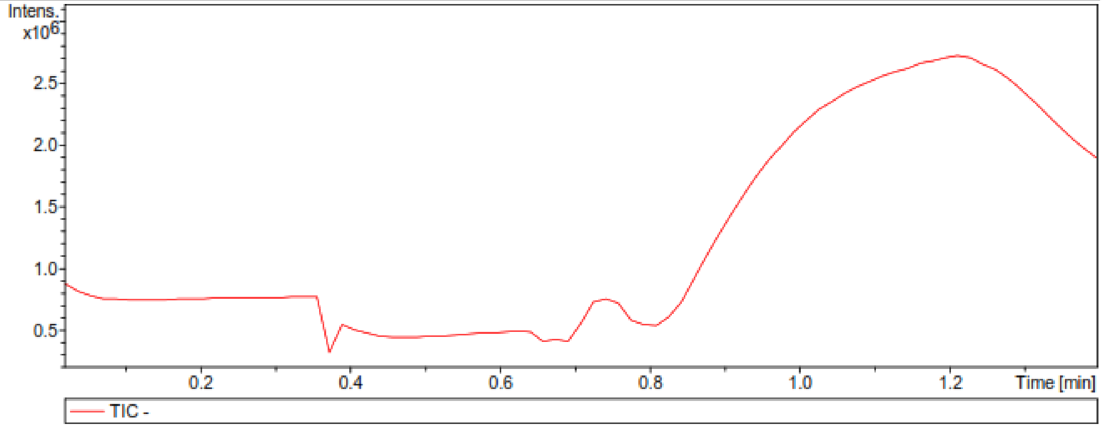

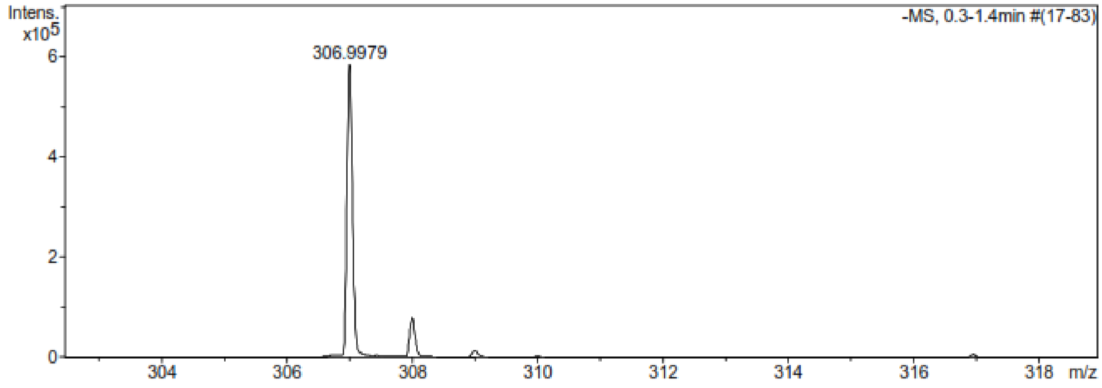

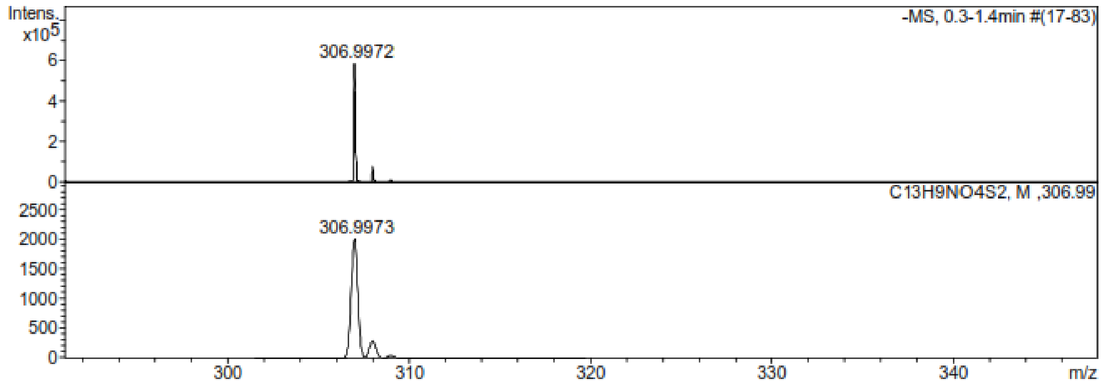

Bruker Compass DataAnalysis 4.0 printed: 2021-12-06 17:04:39 Page 1 of 1

HRMS of **6**

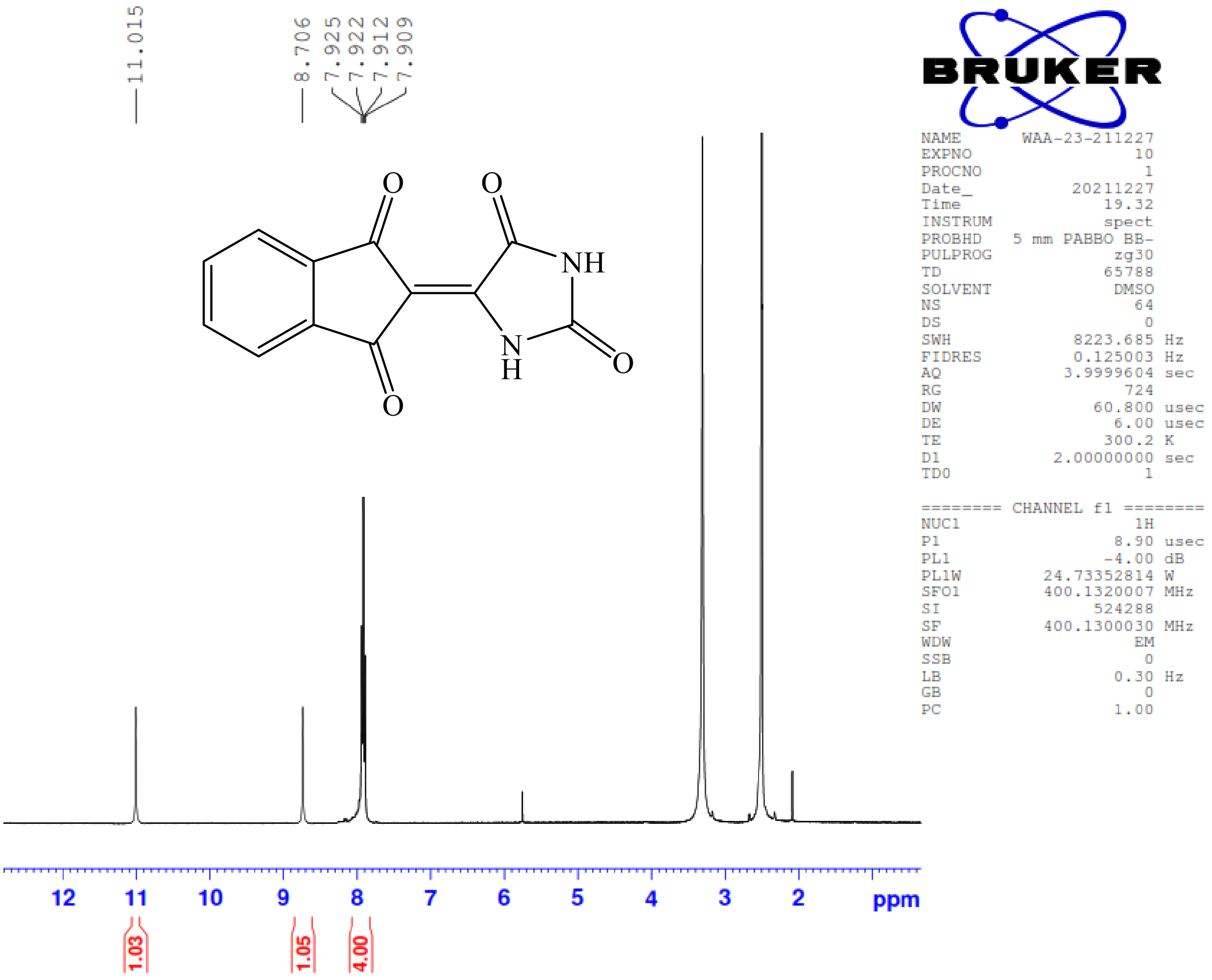

11.015
8.706
7.925
7.922
7.912
7.909
O
O
NH
N
H
O
O
BRUKER
NAME WAA-23-211227
EXPNO 10
PROCNO 1
Date_ 20211227
Time 19.32
INSTRUM spect
PROBHD 5 mm PABBO BB-
PULPROG zg30
TD 65788
SOLVENT DMSO
NS 64
DS 0
SWH 8223.685 Hz
FIDRES 0.125003 Hz
AQ 3.9999604 sec
RG 724
DW 60.800 usec
DE 6.00 usec
TE 300.2 K
D1 2.00000000 sec
TD0 1
======== CHANNEL f1 ========
NUC1 1H
P1 8.90 usec
PL1 -4.00 dB
PL1W 24.73352814 W
SFO1 400.1320007 MHz
SI 524288
SF 400.1300030 MHz
WDW EM
SSB 0
LB 0.30 Hz
GB 0
PC 1.00
12 11 10 9 8 7 6 5 4 3 2 ppm
1.03
1.05
4.00

^{1}H NMR of **8a**

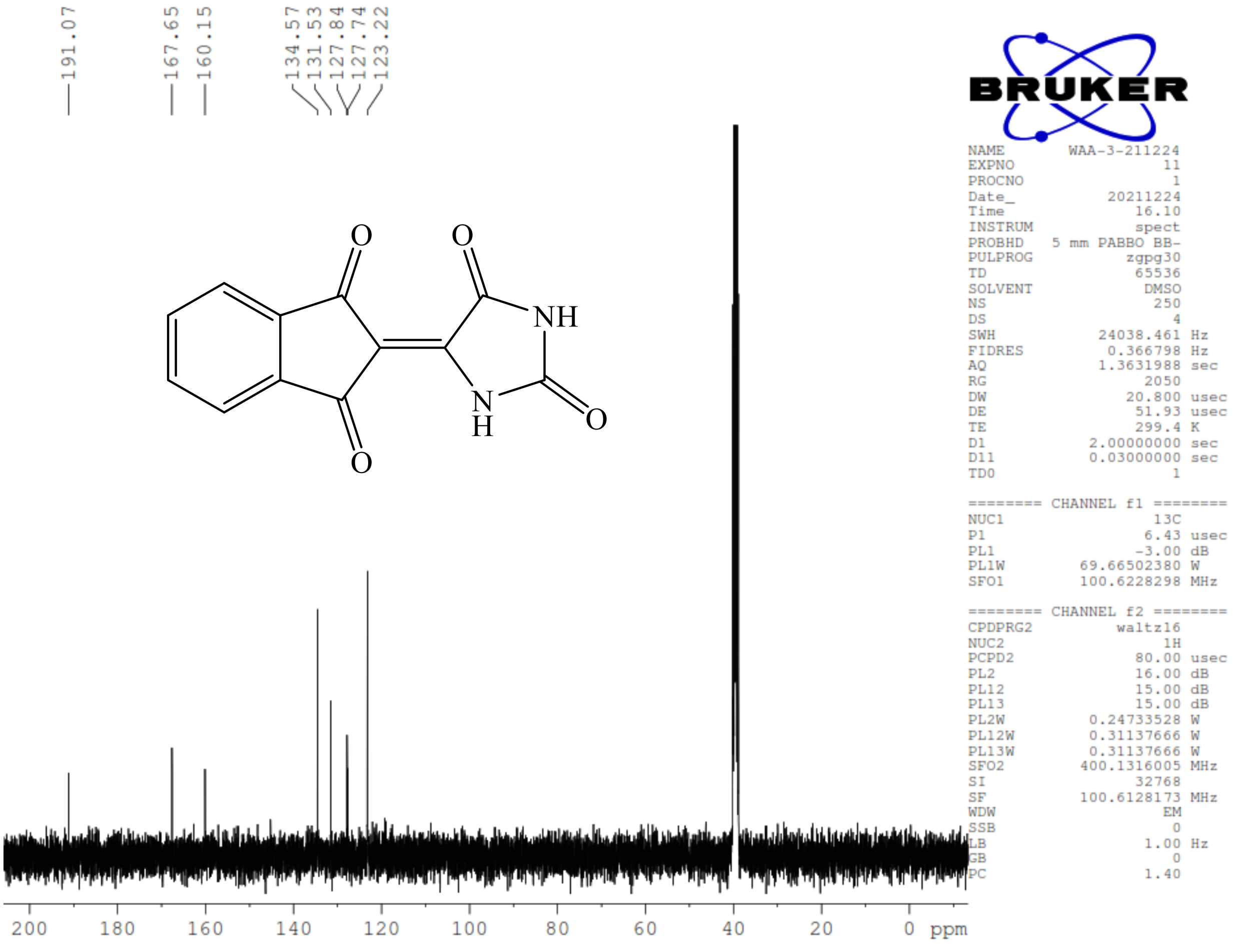
191.07
167.65
160.15
134.57
131.53
127.84
127.74
123.22
O
O
NH
N
H
O
O
200
180
160
140
120
100
80
60
40
20
0
ppm
BRUKER
NAME WAA-3-211224
EXPNO 11
PROCNO 1
Date_ 20211224
Time 16.10
INSTRUM spect
PROBHD 5 mm PABBO BB-
PULPROG zgpg30
TD 65536
SOLVENT DMSO
NS 250
DS 4
SWH 24038.461 Hz
FIDRES 0.366798 Hz
AQ 1.3631988 sec
RG 2050
DW 20.800 usec
DE 51.93 usec
TE 299.4 K
D1 2.00000000 sec
D11 0.03000000 sec
TD0 1
======== CHANNEL f1 ========
NUC1 13C
P1 6.43 usec
PL1 -3.00 dB
PL1W 69.66502380 W
SFO1 100.6228298 MHz
======== CHANNEL f2 ========
CPDPRG2 waltz16
NUC2 1H
PCPD2 80.00 usec
PL2 16.00 dB
PL12 15.00 dB
PL13 15.00 dB
PL2W 0.24733528 W
PL12W 0.31137666 W
PL13W 0.31137666 W
SFO2 400.1316005 MHz
SI 32768
SF 100.6128173 MHz
WDW EM
SSB 0
LB 1.00 Hz
GB 0
PC 1.40

^{13}C NMR of **8a**

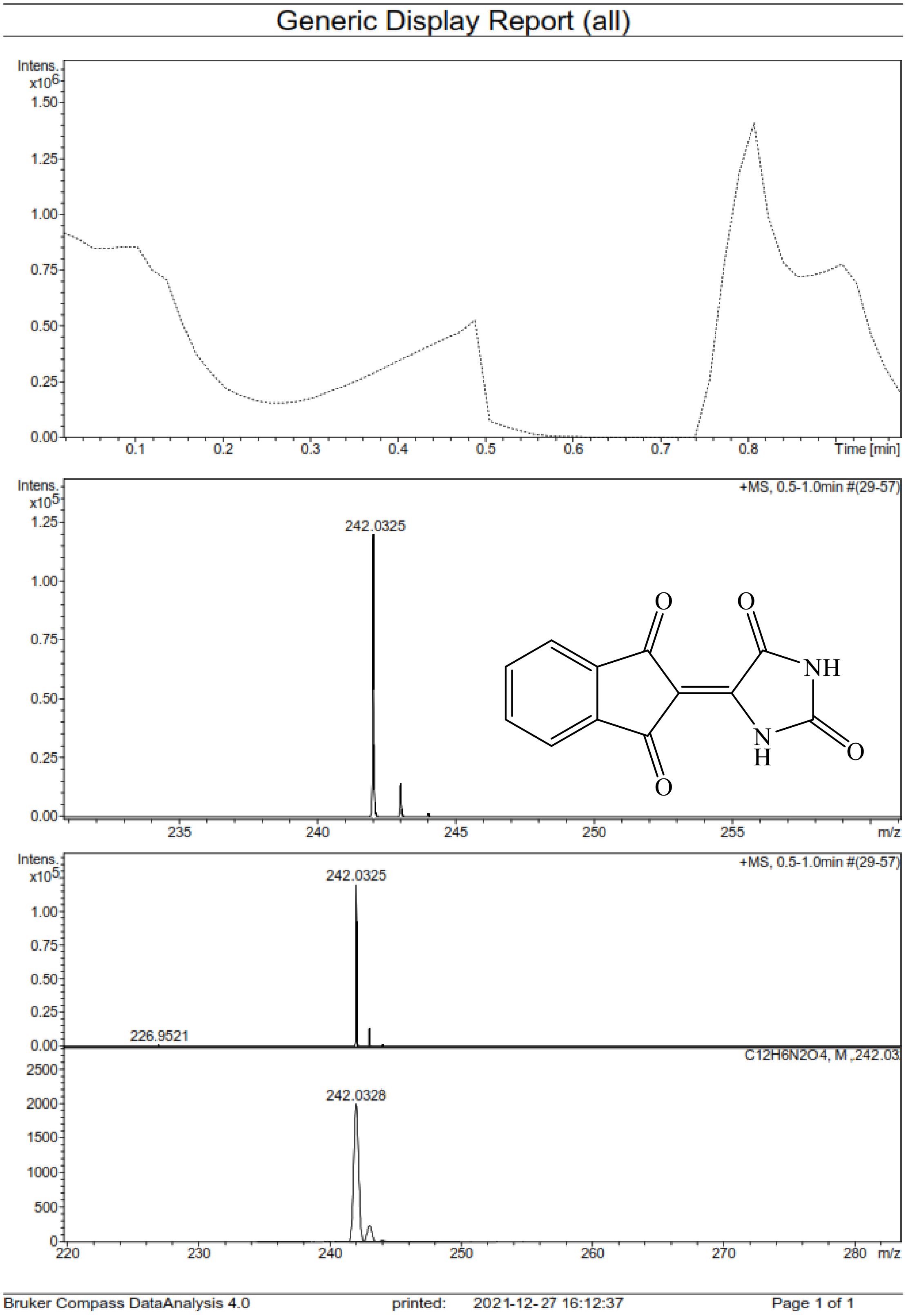

Generic Display Report (all)
Intens. x10^6
Time [min]
+MS, 0.5-1.0min #(29-57)
242.0325
226.9521
C12H6N2O4, M ,242.03
242.0328
m/z
Bruker Compass DataAnalysis 4.0
printed: 2021-12-27 16:12:37
Page 1 of 1

HRMS of **8a**

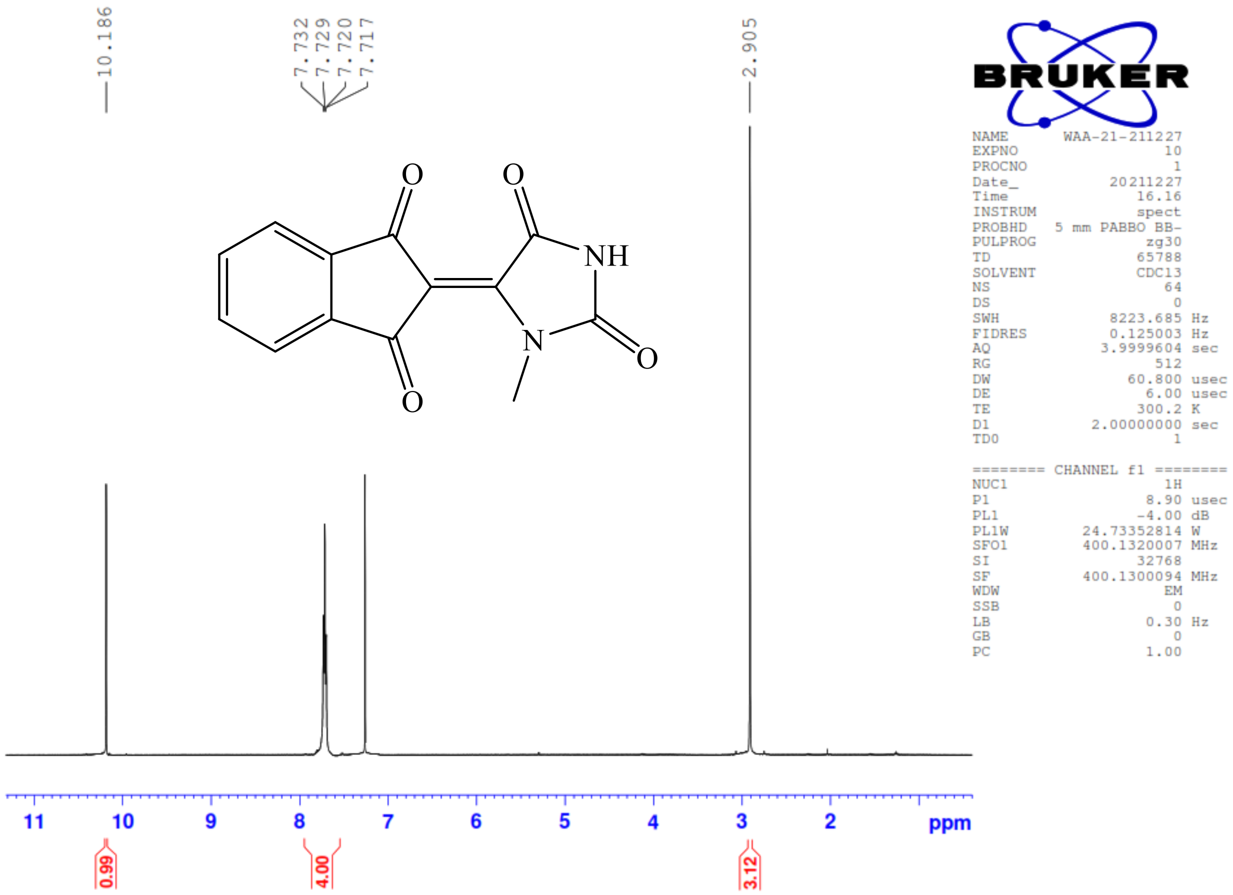
10.186
7.732
7.729
7.720
7.717
2.905
O
O
NH
N
O
O
BRUKER
NAME WAA-21-211227
EXPNO 10
PROCNO 1
Date_ 20211227
Time 16.16
INSTRUM spect
PROBHD 5 mm PABBO BB-
PULPROG zg30
TD 65788
SOLVENT CDCl3
NS 64
DS 0
SWH 8223.685 Hz
FIDRES 0.125003 Hz
AQ 3.9999604 sec
RG 512
DW 60.800 usec
DE 6.00 usec
TE 300.2 K
D1 2.00000000 sec
TD0 1
======== CHANNEL f1 ========
NUC1 1H
P1 8.90 usec
PL1 -4.00 dB
PL1W 24.73352814 W
SFO1 400.1320007 MHz
SI 32768
SF 400.1300094 MHz
WDW EM
SSB 0
LB 0.30 Hz
GB 0
PC 1.00
11
10
9
8
7
6
5
4
3
2
ppm
0.99
4.00
3.12

^{1}H NMR of **8b**

Display Report

Analysis Info

Analysis Name	E:\Data2\wael\59-.d	Acquisition Date	2021-12-26 16:02:12
Method	Tune_low_neg.m	Operator	AD
Sample Name	59-	Instrument / Ser#	micrOTOF 125
Comment			

Acquisition Parameter

Source Type	ESI	Ion Polarity	Negative	Set Nebulizer	0.3 Bar
Focus	Not active			Set Dry Heater	180 °C
Scan Begin	50 m/z	Set Capillary	4000 V	Set Dry Gas	4.0 l/min
Scan End	1500 m/z	Set End Plate Offset	-500 V	Set Divert Valve	Waste

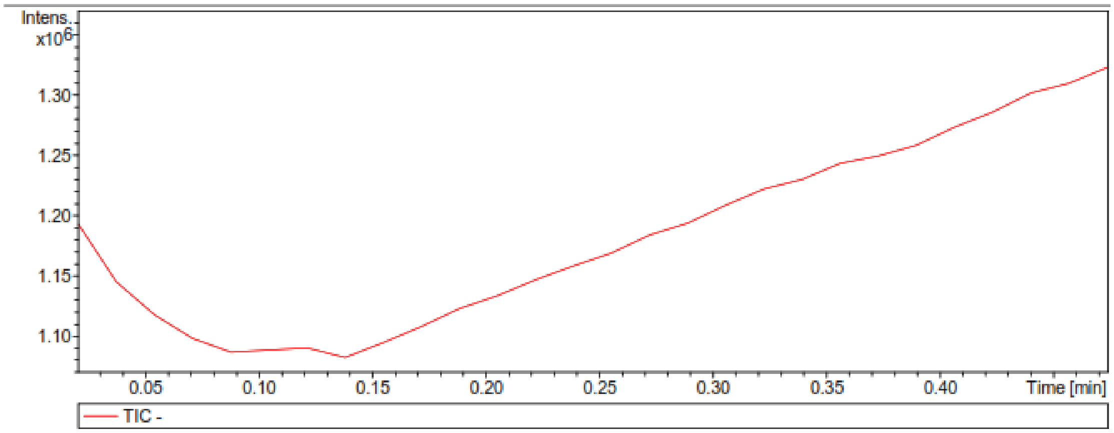

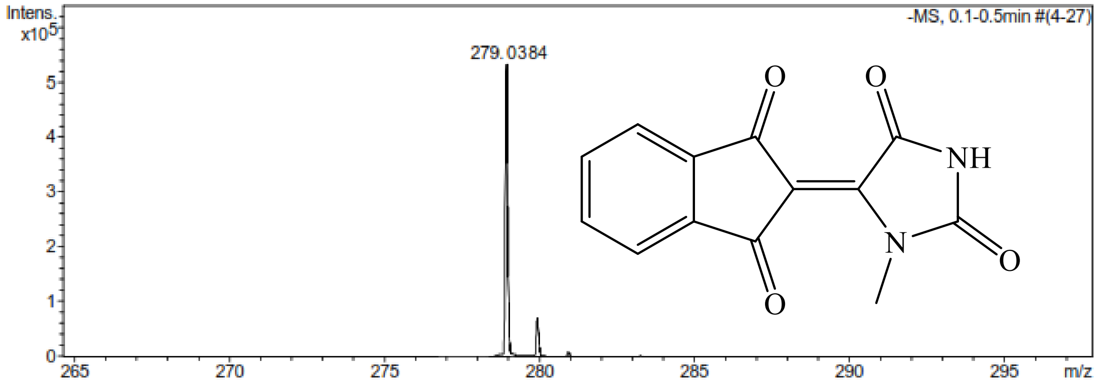

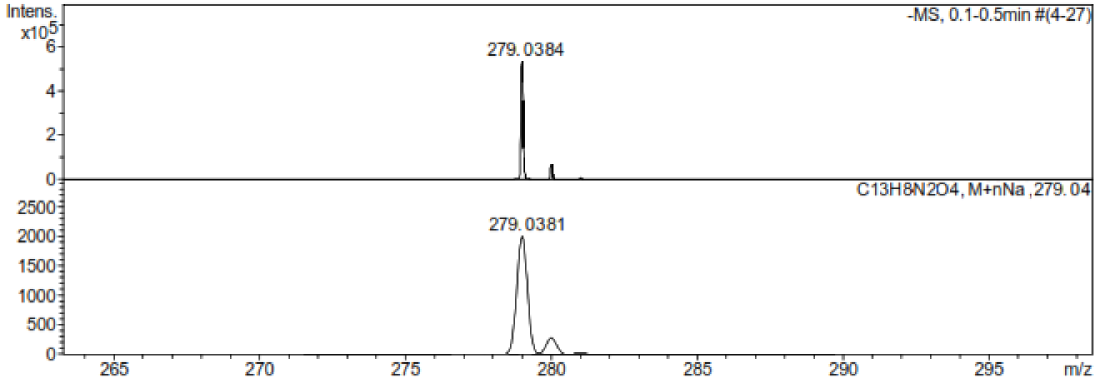

Bruker Compass DataAnalysis 4.0 printed: 2021-12-26 17:06:15 Page 1 of 1

HRMS of **8b**

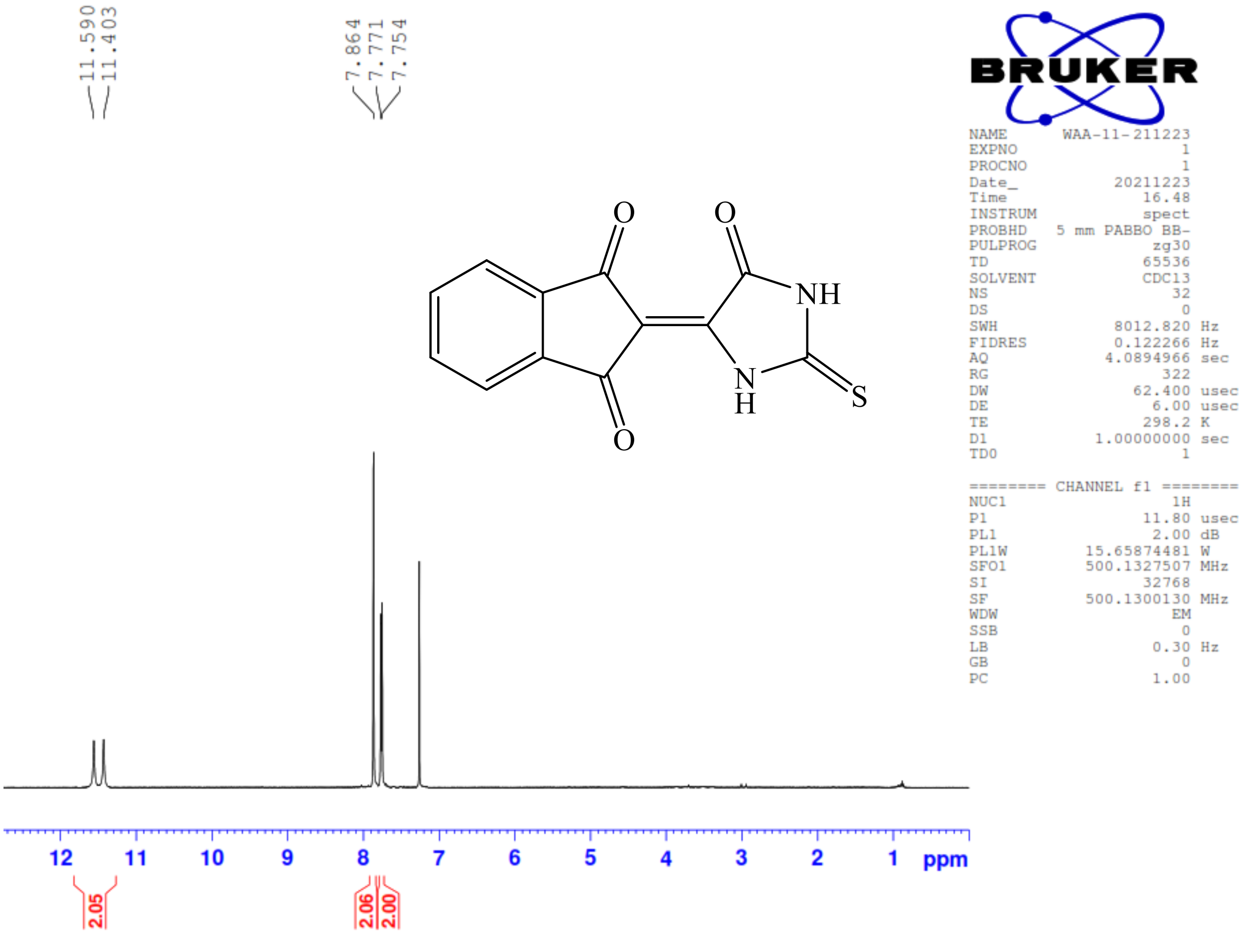
11.590
11.403
7.864
7.771
7.754
NH
N
H
S
O
O
O
BRUKER
NAME WAA-11-211223
EXPNO 1
PROCNO 1
Date_ 20211223
Time 16.48
INSTRUM spect
PROBHD 5 mm PABBO BB-
PULPROG zg30
TD 65536
SOLVENT CDCl3
NS 32
DS 0
SWH 8012.820 Hz
FIDRES 0.122266 Hz
AQ 4.0894966 sec
RG 322
DW 62.400 usec
DE 6.00 usec
TE 298.2 K
D1 1.00000000 sec
TD0 1
======== CHANNEL f1 ========
NUC1 1H
P1 11.80 usec
PL1 2.00 dB
PL1W 15.65874481 W
SFO1 500.1327507 MHz
SI 32768
SF 500.1300130 MHz
WDW EM
SSB 0
LB 0.30 Hz
GB 0
PC 1.00
12 11 10 9 8 7 6 5 4 3 2 1 ppm
2.05
2.06
2.00

^{1}H NMR of **8c**

Generic Display Report

Analysis Info

Analysis Name	E:\Data2\wael\-85.d	Acquisition Date	2021-12-27 14:06:48
Method	Tune_wide_neg.m	Operator	AD
Sample Name	-85	Instrument	micrOTOF
Comment			

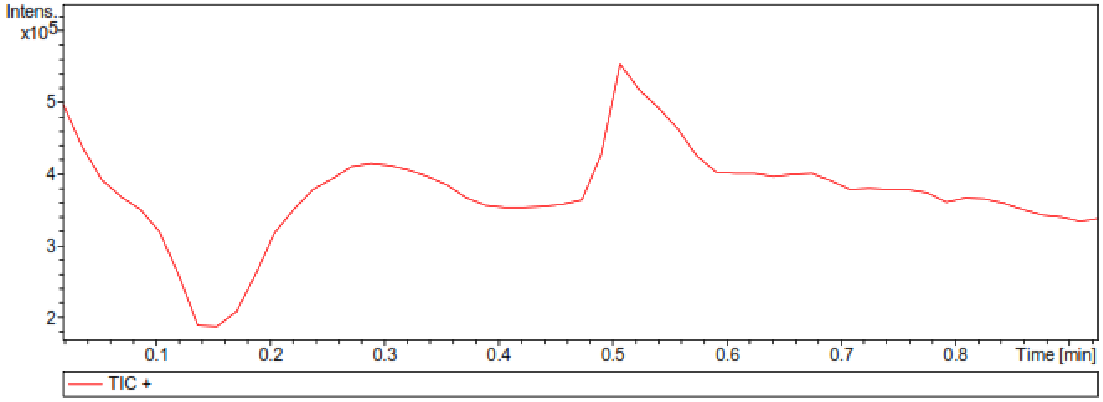

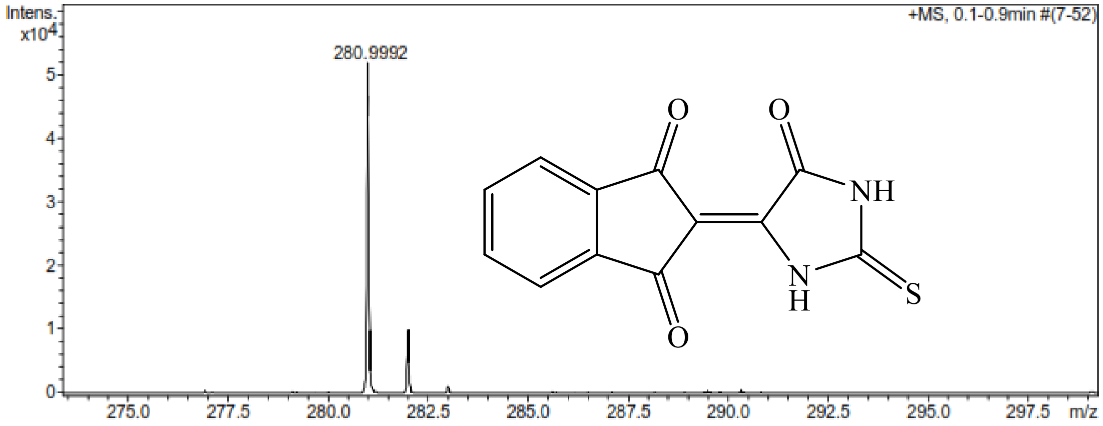

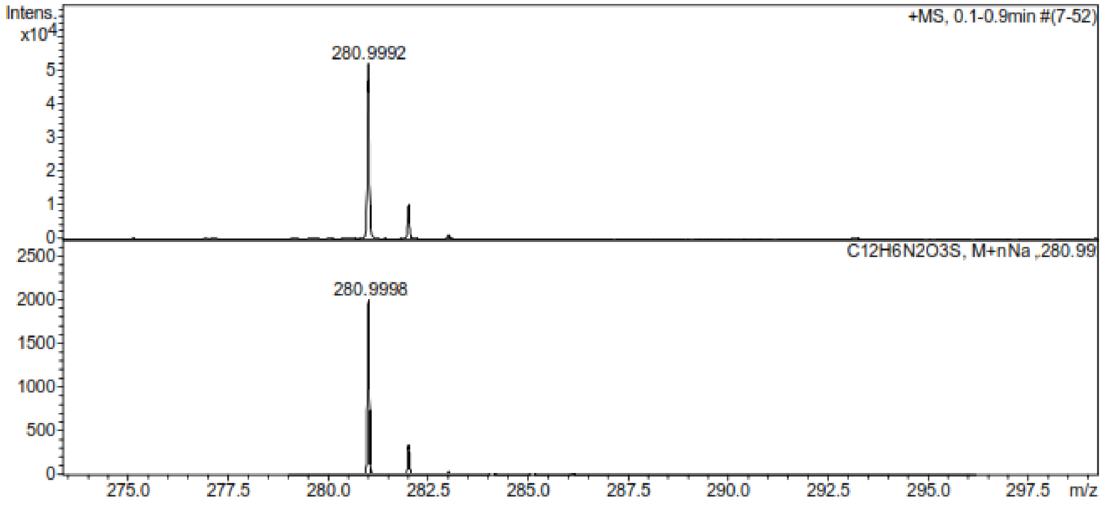

Bruker Compass DataAnalysis 4.0 printed: 2021-12-27 14:12:14 Page 1 of 1

HRMS of **8c**

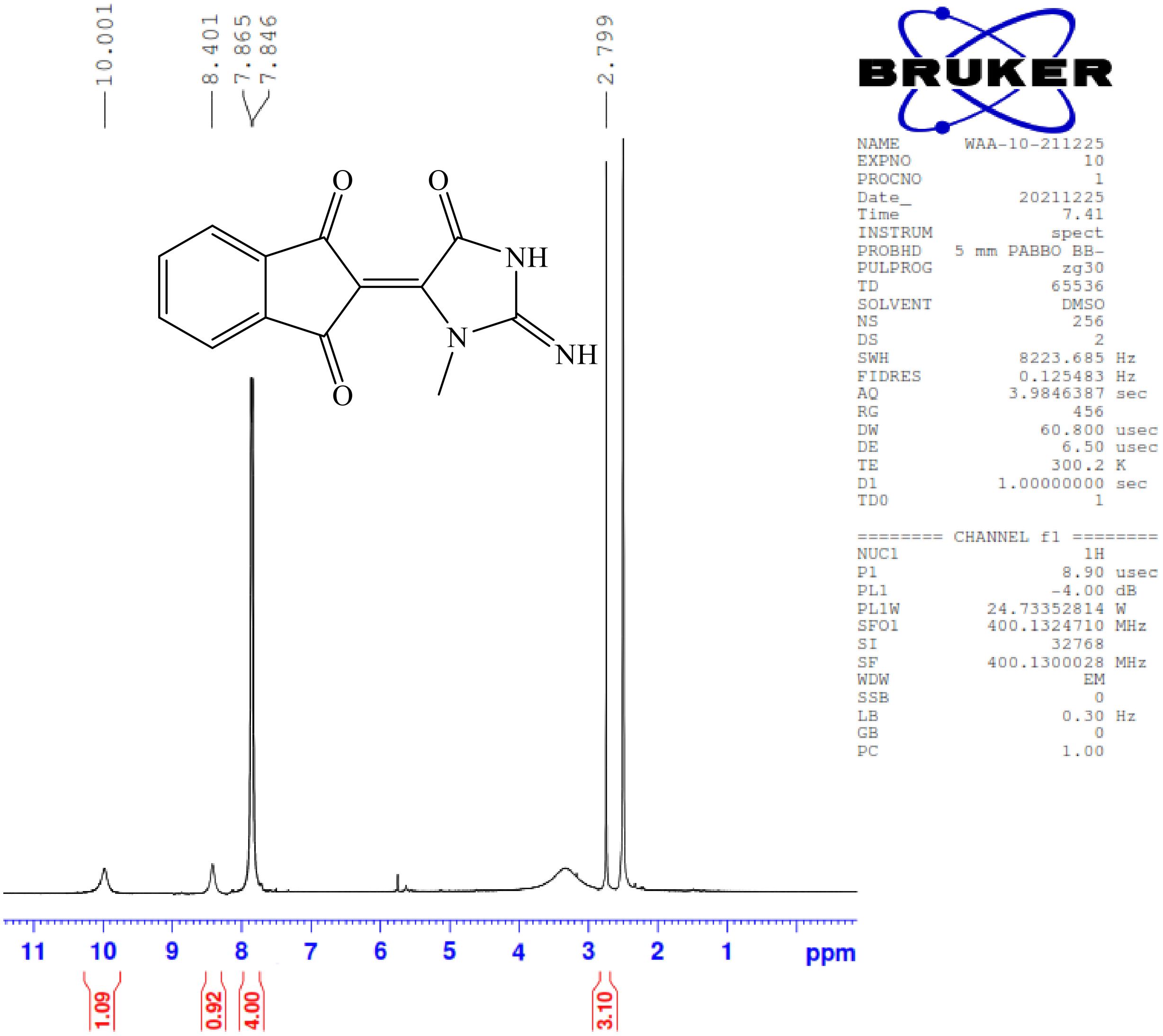
10.001
8.401
7.865
7.846
2.799
O
O
NH
N
NH
O
NAME WAA-10-211225
EXPNO 10
PROCNO 1
Date_ 20211225
Time 7.41
INSTRUM spect
PROBHD 5 mm PABBO BB-
PULPROG zg30
TD 65536
SOLVENT DMSO
NS 256
DS 2
SWH 8223.685 Hz
FIDRES 0.125483 Hz
AQ 3.9846387 sec
RG 456
DW 60.800 usec
DE 6.50 usec
TE 300.2 K
D1 1.00000000 sec
TD0 1
======== CHANNEL f1 ========
NUC1 1H
P1 8.90 usec
PL1 -4.00 dB
PL1W 24.73352814 W
SFO1 400.1324710 MHz
SI 32768
SF 400.1300028 MHz
WDW EM
SSB 0
LB 0.30 Hz
GB 0
PC 1.00
11
10
9
8
7
6
5
4
3
2
1
ppm
1.09
0.92
4.00
3.10

^{1}H NMR of **8d**

Display Report

Analysis Info

Analysis Name	E:\Data2\wael\91-.d	Acquisition Date	2021-12-26 16:02:12
Method	Tune_wide_neg.m	Operator	AD
Sample Name	91-	Instrument / Ser#	micrOTOF 125
Comment			

Acquisition Parameter

Source Type	ESI	Ion Polarity	Negative	Set Nebulizer	0.3 Bar
Focus	Not active			Set Dry Heater	180 °C
Scan Begin	50 m/z	Set Capillary	4000 V	Set Dry Gas	4.0 l/min
Scan End	1500 m/z	Set End Plate Offset	-500 V	Set Divert Valve	Waste

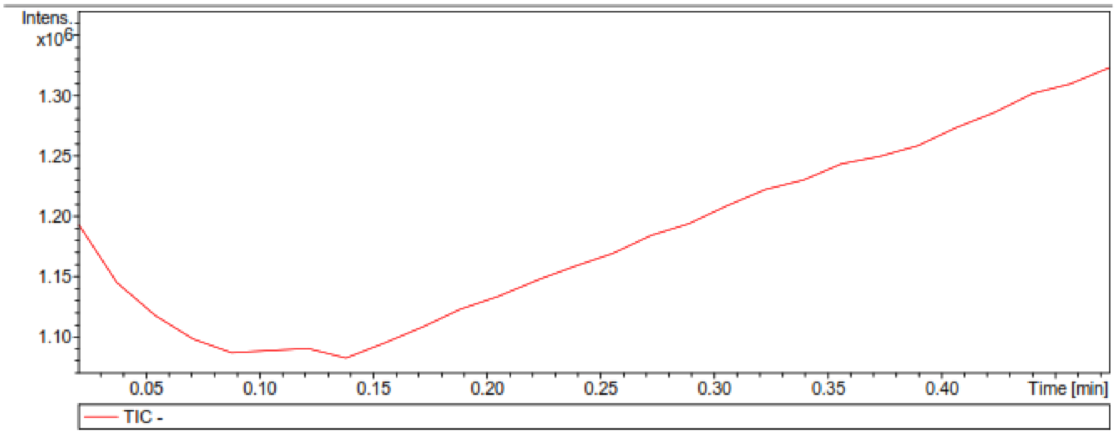

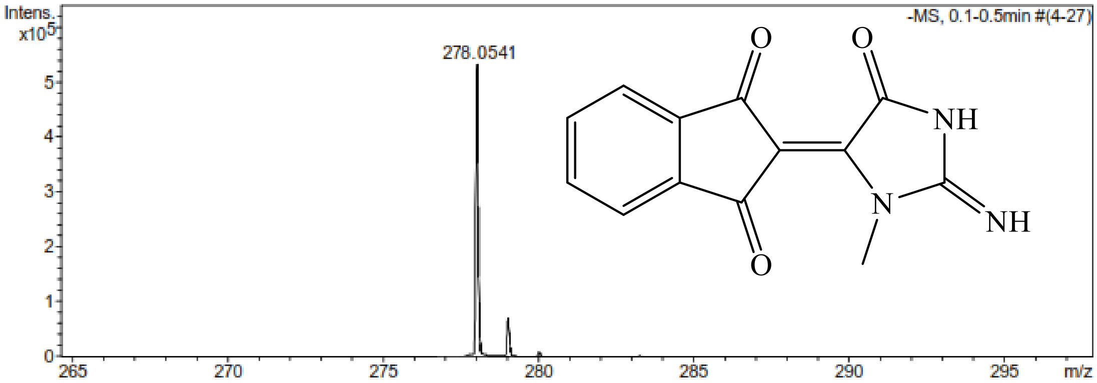

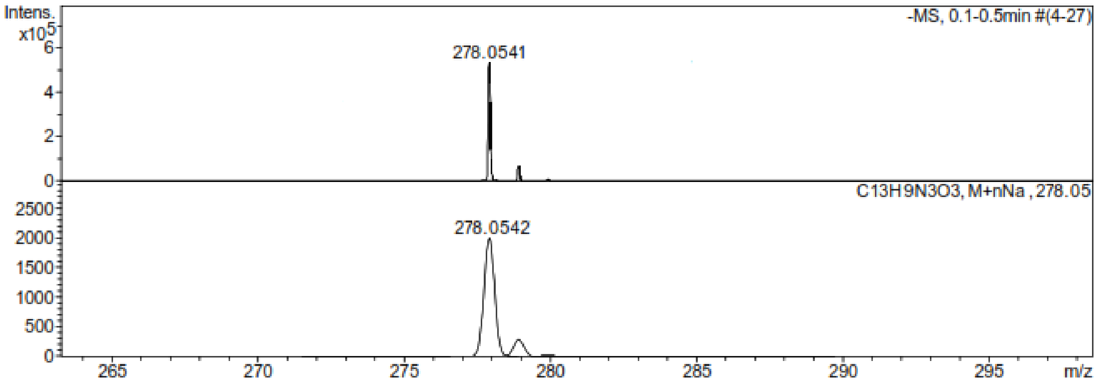

Bruker Compass DataAnalysis 4.0 printed: 2021-12-26 17:06:15 Page 1 of 1

HRMS of **8d**

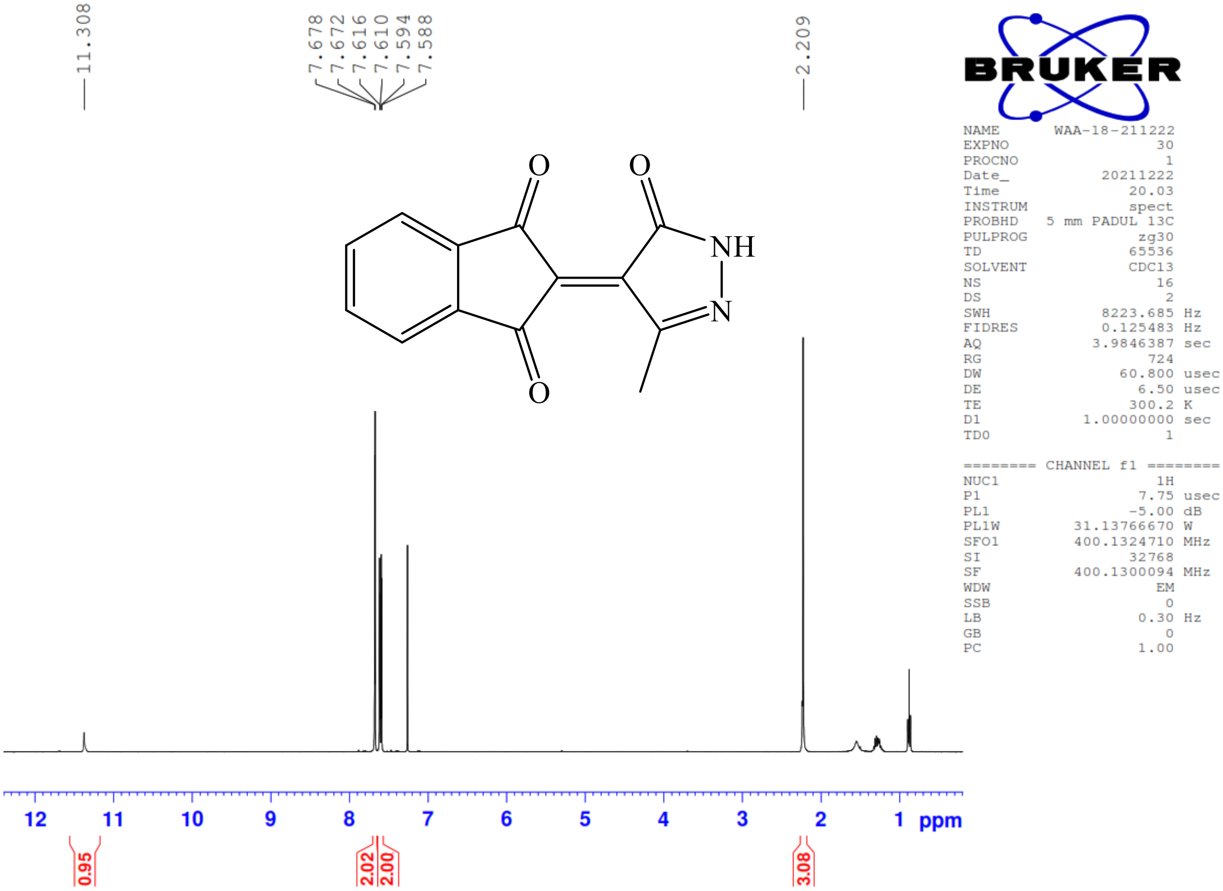
11.308
7.678
7.672
7.616
7.610
7.594
7.588
2.209
O
O
NH
N
O
BRUKER
NAME WAA-18-211222
EXPNO 30
PROCNO 1
Date_ 20211222
Time 20.03
INSTRUM spect
PROBHD 5 mm PADUL 13C
PULPROG zg30
TD 65536
SOLVENT CDCl3
NS 16
DS 2
SWH 8223.685 Hz
FIDRES 0.125483 Hz
AQ 3.9846387 sec
RG 724
DW 60.800 usec
DE 6.50 usec
TE 300.2 K
D1 1.00000000 sec
TD0 1
======== CHANNEL f1 ========
NUC1 1H
P1 7.75 usec
PL1 -5.00 dB
PL1W 31.13766670 W
SFO1 400.1324710 MHz
SI 32768
SF 400.1300094 MHz
WDW EM
SSB 0
LB 0.30 Hz
GB 0
PC 1.00
12 11 10 9 8 7 6 5 4 3 2 1 ppm
0.95
2.02
2.00
3.08

^{1}H NMR of **10a**

Generic Display Report

Analysis Info

Analysis Name	E:\Data2\arafa\79p.d	Acquisition Date	2021-12-27 20:00:12
Method	tune_low_dirk.m	Operator	AD
Sample Name	79p	Instrument	micrOTOF
Comment	TML-A-003		

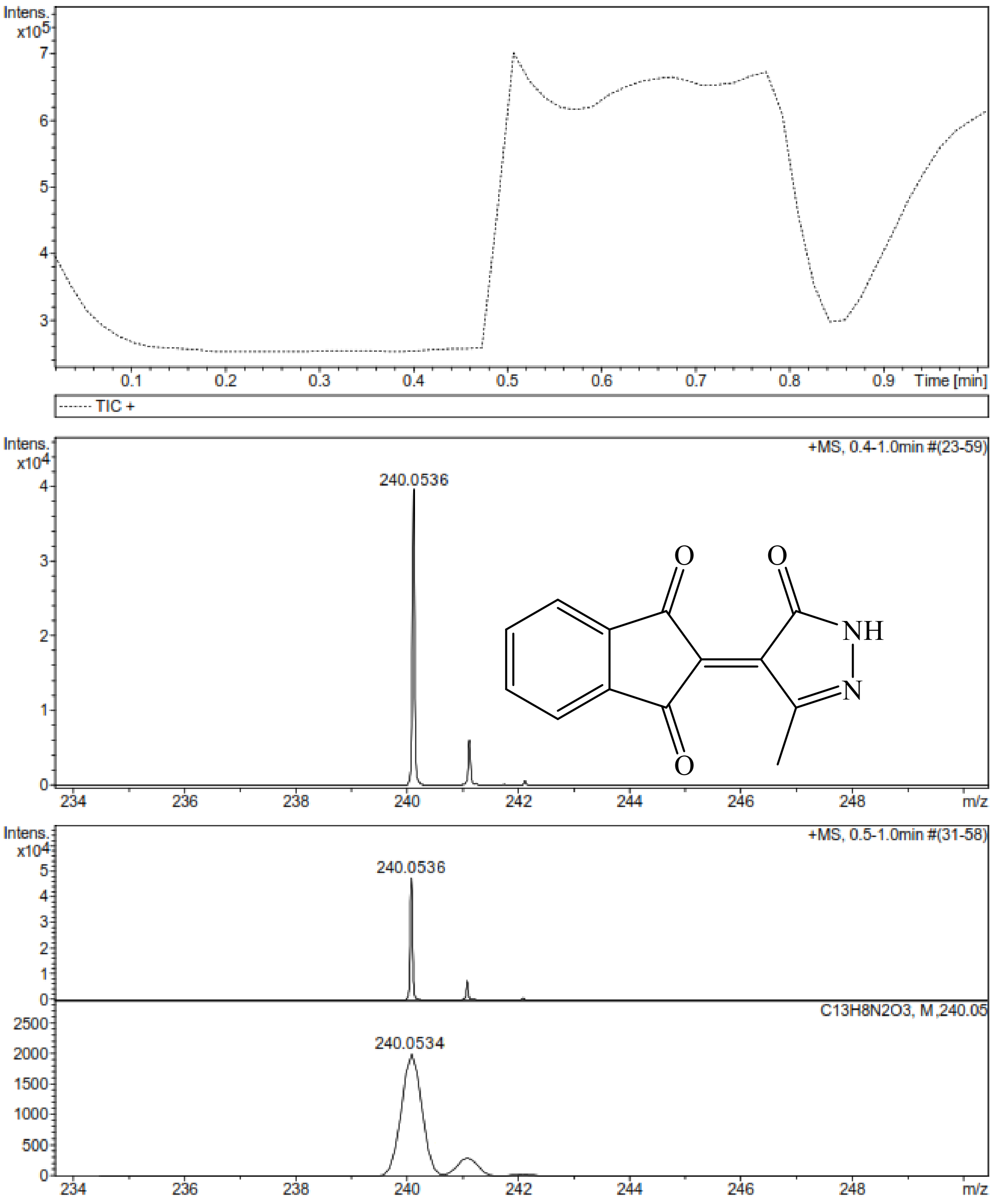

Bruker Compass DataAnalysis 4.0 printed: 2021-12-27 13:32:25 Page 1 of 1

HRMS of **10a**

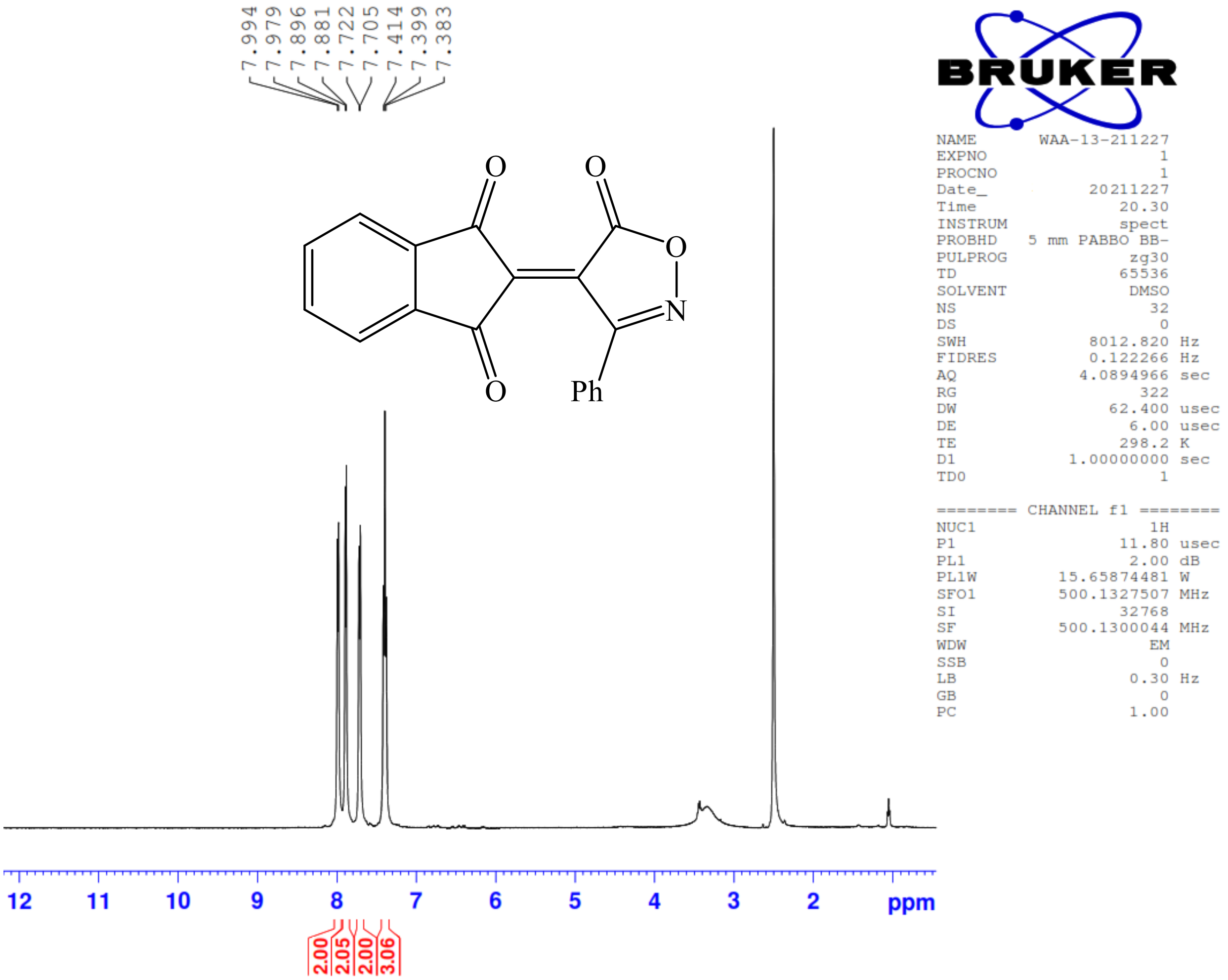
7.994
7.979
7.896
7.881
7.722
7.705
7.414
7.399
7.383
O
O
O
O
N
Ph
NAME WAA-13-211227
EXPNO 1
PROCNO 1
Date_ 20211227
Time 20.30
INSTRUM spect
PROBHD 5 mm PABBO BB-
PULPROG zg30
TD 65536
SOLVENT DMSO
NS 32
DS 0
SWH 8012.820 Hz
FIDRES 0.122266 Hz
AQ 4.0894966 sec
RG 322
DW 62.400 usec
DE 6.00 usec
TE 298.2 K
D1 1.00000000 sec
TD0 1
======== CHANNEL f1 ========
NUC1 1H
P1 11.80 usec
PL1 2.00 dB
PL1W 15.65874481 W
SFO1 500.1327507 MHz
SI 32768
SF 500.1300044 MHz
WDW EM
SSB 0
LB 0.30 Hz
GB 0
PC 1.00
12
11
10
9
8
7
6
5
4
3
2
ppm
2.00
2.05
2.00
3.06

^{1}H NMR of **10b**

Display Report

Analysis Info

Analysis Name	E:\Data2\wael\50.d	Acquisition Date	2021-12-26 16:09:45
Method	Tune_low_neg.m	Operator	AD
Sample Name	50	Instrument / Ser#	micrOTOF 125
Comment			

Acquisition Parameter

Source Type	ESI	Ion Polarity	Negative	Set Nebulizer	0.3 Bar
Focus	Not active			Set Dry Heater	180 °C
Scan Begin	50 m/z	Set Capillary	4000 V	Set Dry Gas	4.0 l/min
Scan End	1500 m/z	Set End Plate Offset	-500 V	Set Divert Valve	Waste

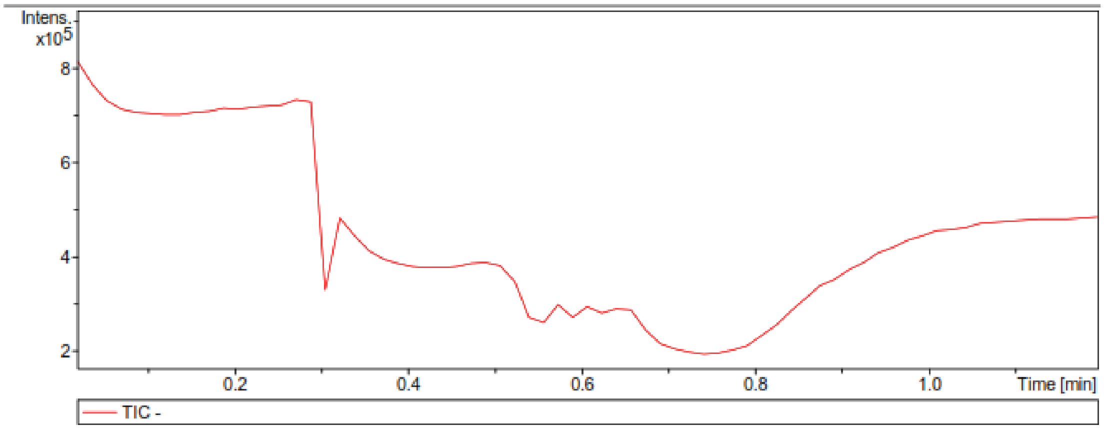

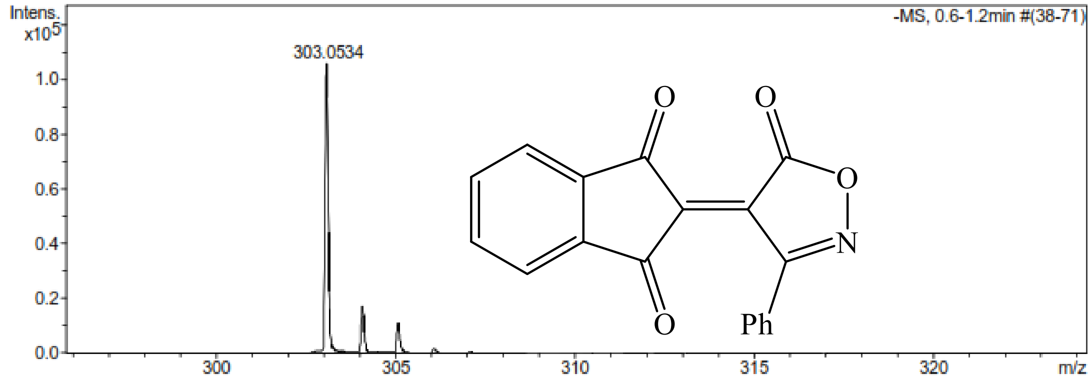

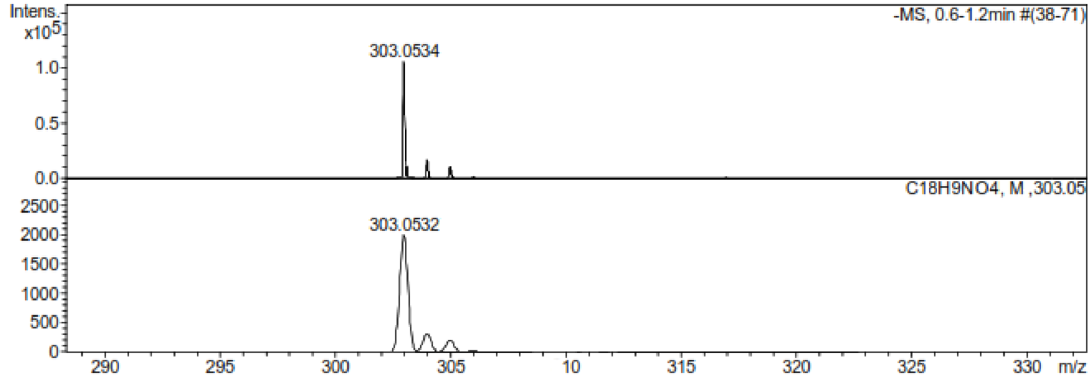

Bruker Compass DataAnalysis 4.0 printed: 2021-12-26 16:53:23 Page 1 of 1

HRMS of **10b**

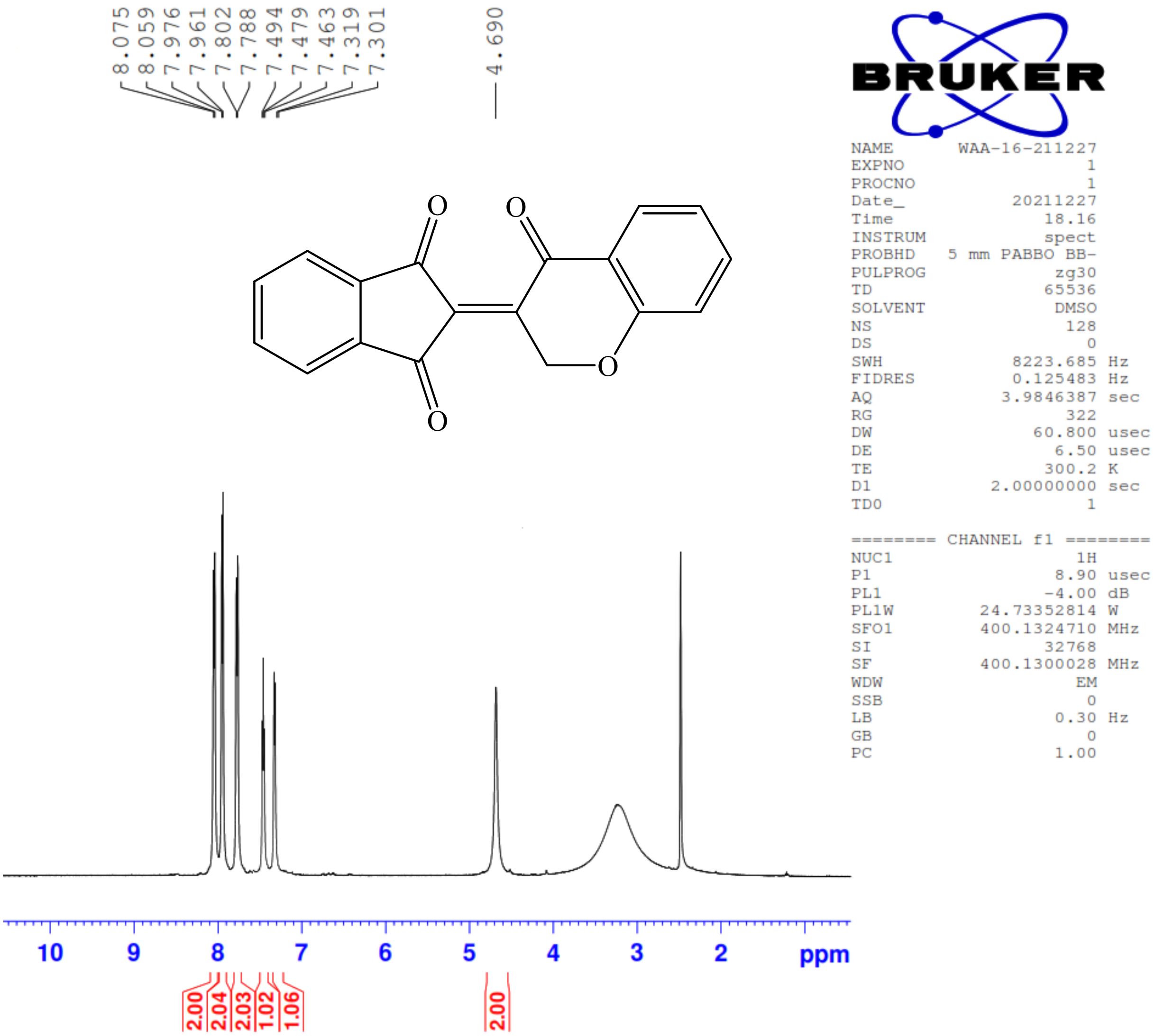
8.075
8.059
7.976
7.961
7.802
7.788
7.494
7.479
7.463
7.319
7.301
4.690
O
O
O
O
BRUKER
NAME WAA-16-211227
EXPNO 1
PROCNO 1
Date_ 20211227
Time 18.16
INSTRUM spect
PROBHD 5 mm PABBO BB-
PULPROG zg30
TD 65536
SOLVENT DMSO
NS 128
DS 0
SWH 8223.685 Hz
FIDRES 0.125483 Hz
AQ 3.9846387 sec
RG 322
DW 60.800 usec
DE 6.50 usec
TE 300.2 K
D1 2.00000000 sec
TD0 1
======== CHANNEL f1 ========
NUC1 1H
P1 8.90 usec
PL1 -4.00 dB
PL1W 24.73352814 W
SFO1 400.1324710 MHz
SI 32768
SF 400.1300028 MHz
WDW EM
SSB 0
LB 0.30 Hz
GB 0
PC 1.00
10
9
8
7
6
5
4
3
2
ppm
2.00
2.04
2.03
1.02
1.06
2.00

^{1}H NMR of **12a**

Generic Display Report

Analysis Info

Analysis Name	E:\Data2\arafa\75.d	Acquisition Date	2021-12-27 13:40:13
Method	tune_low_dirk.m	Operator	AD
Sample Name	75	Instrument	micrOTOF
Comment			

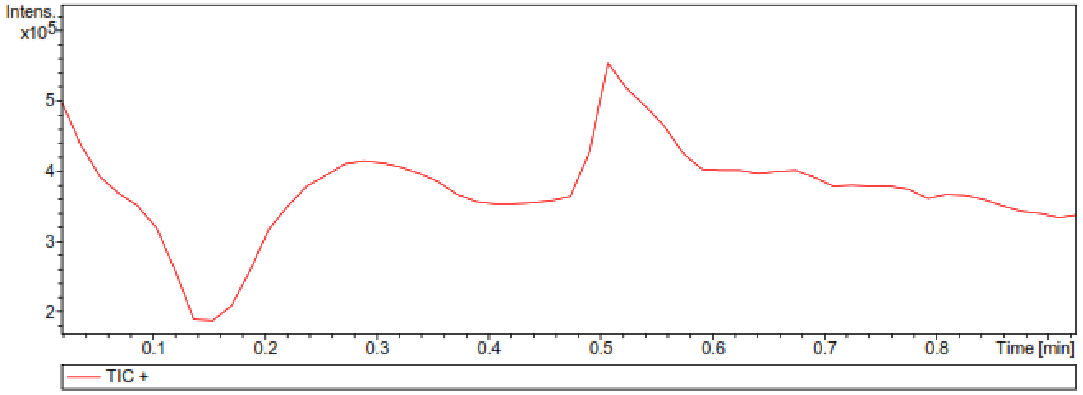

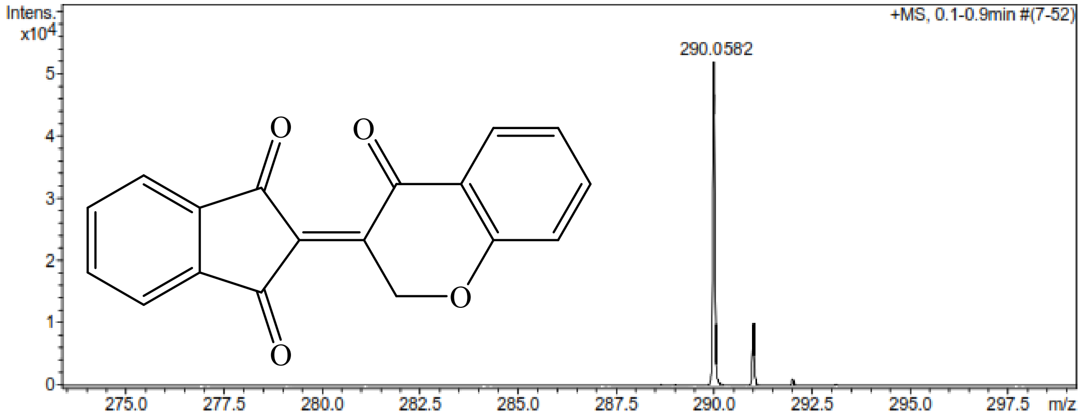

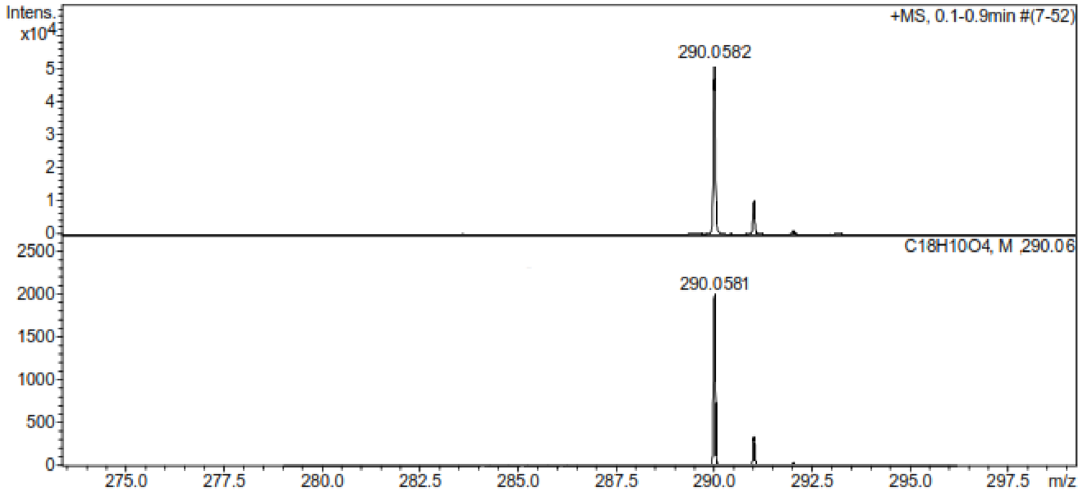

Bruker Compass DataAnalysis 4.0 printed: 2021-12-27 13:17:22 Page 1 of 1

HRMS of **12a**

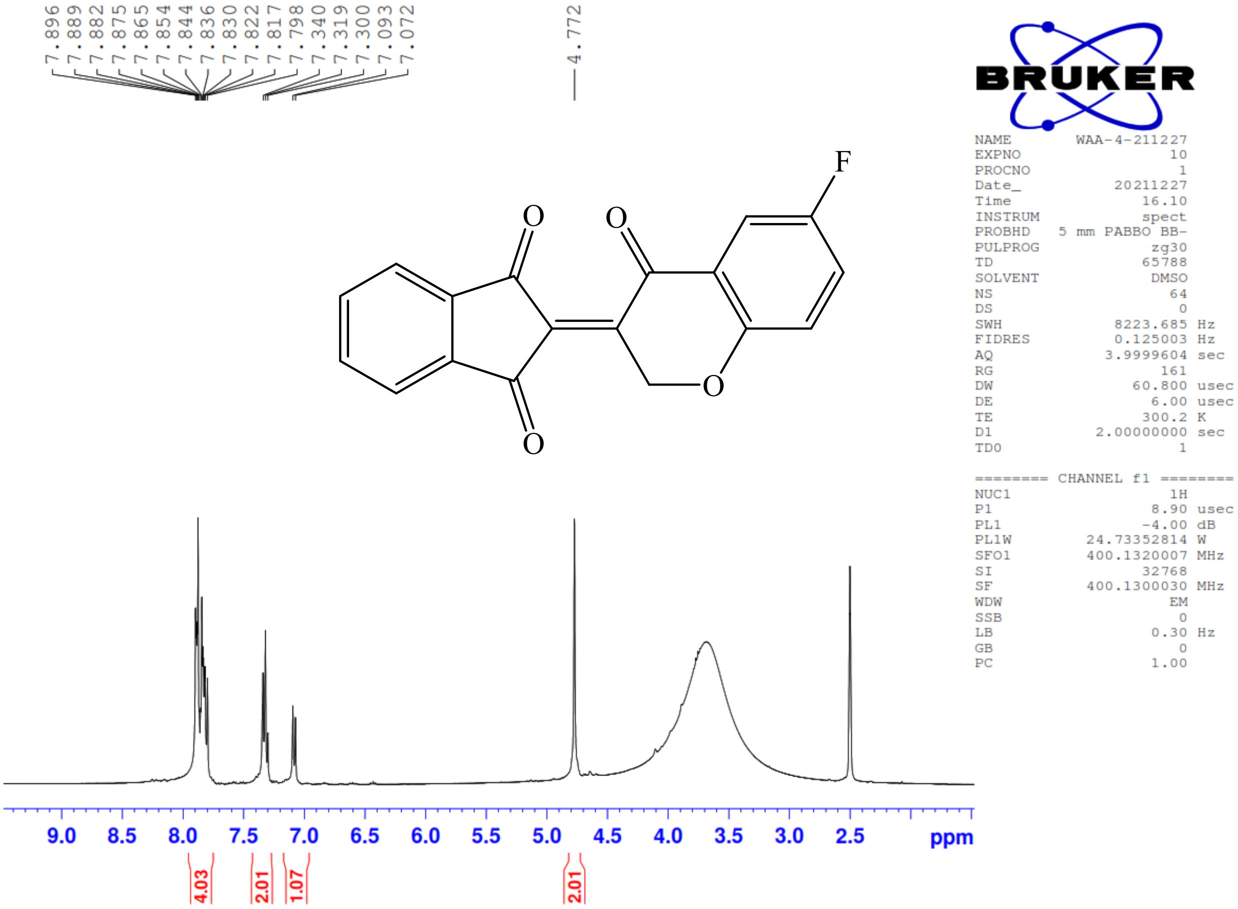
7.896
7.889
7.882
7.875
7.865
7.854
7.844
7.836
7.830
7.822
7.817
7.798
7.340
7.319
7.300
7.093
7.072
4.772
F
O
O
O
O
BRUKER
NAME WAA-4-211227
EXPNO 10
PROCNO 1
Date_ 20211227
Time 16.10
INSTRUM spect
PROBHD 5 mm PABBO BB-
PULPROG zg30
TD 65788
SOLVENT DMSO
NS 64
DS 0
SWH 8223.685 Hz
FIDRES 0.125003 Hz
AQ 3.9999604 sec
RG 161
DW 60.800 usec
DE 6.00 usec
TE 300.2 K
D1 2.00000000 sec
TD0 1
======== CHANNEL f1 ========
NUC1 1H
P1 8.90 usec
PL1 -4.00 dB
PL1W 24.73352814 W
SFO1 400.1320007 MHz
SI 32768
SF 400.1300030 MHz
WDW EM
SSB 0
LB 0.30 Hz
GB 0
PC 1.00
9.0 8.5 8.0 7.5 7.0 6.5 6.0 5.5 5.0 4.5 4.0 3.5 3.0 2.5 ppm
4.03
2.01
1.07
2.01

^{1}H NMR of **12b**

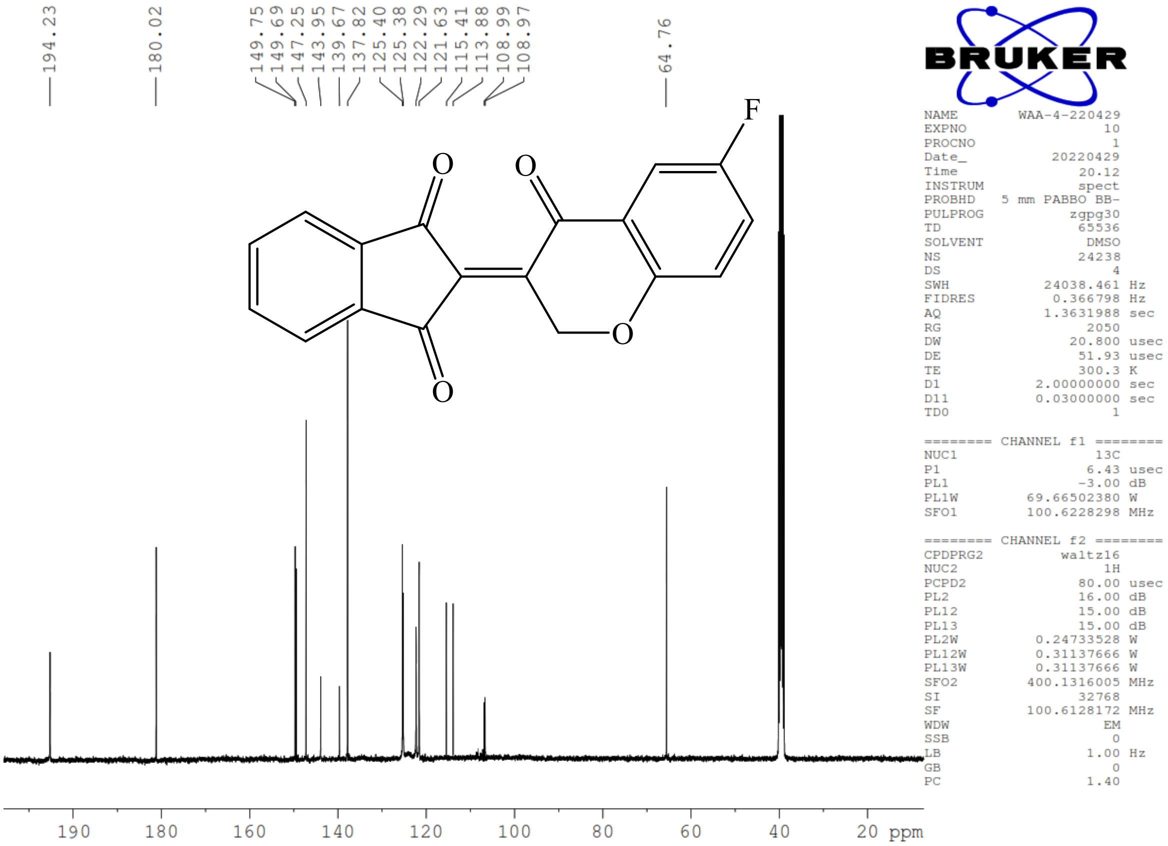

^{13}C NMR of **12b**

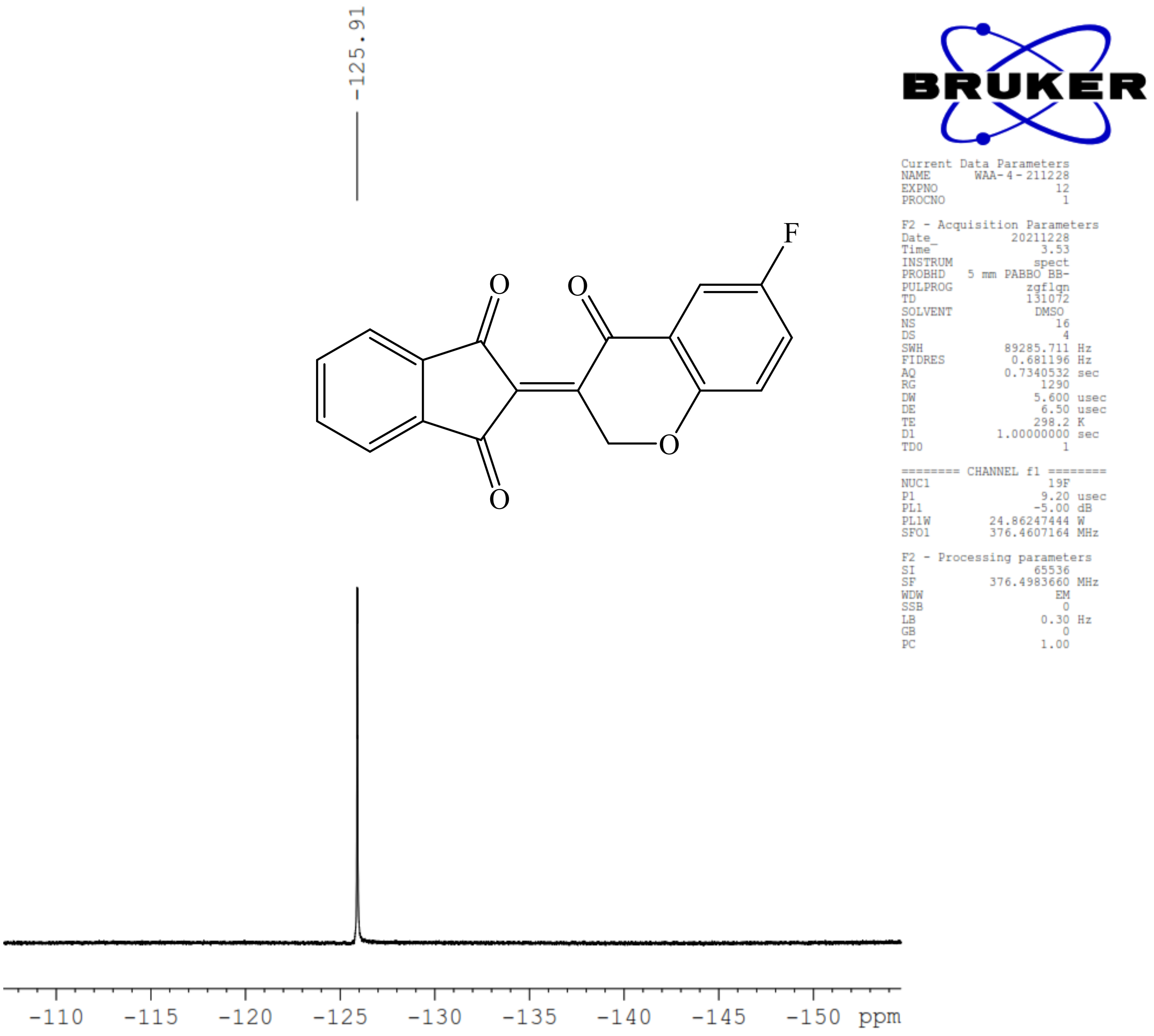

^{19}F NMR of **12b**

Display Report

Analysis Info

Analysis Name	E:\Data2\wael\80-.d	Acquisition Date	2021-12-26 16:17:04
Method	Tune_wide_neg.m	Operator	AD
Sample Name	80-	Instrument / Ser#	micrOTOF 125
Comment			

Acquisition Parameter

Source Type	ESI	Ion Polarity	Negative	Set Nebulizer	0.3 Bar
Focus	Not active			Set Dry Heater	180 °C
Scan Begin	50 m/z	Set Capillary	4000 V	Set Dry Gas	4.0 l/min
Scan End	1500 m/z	Set End Plate Offset	-500 V	Set Divert Valve	Waste

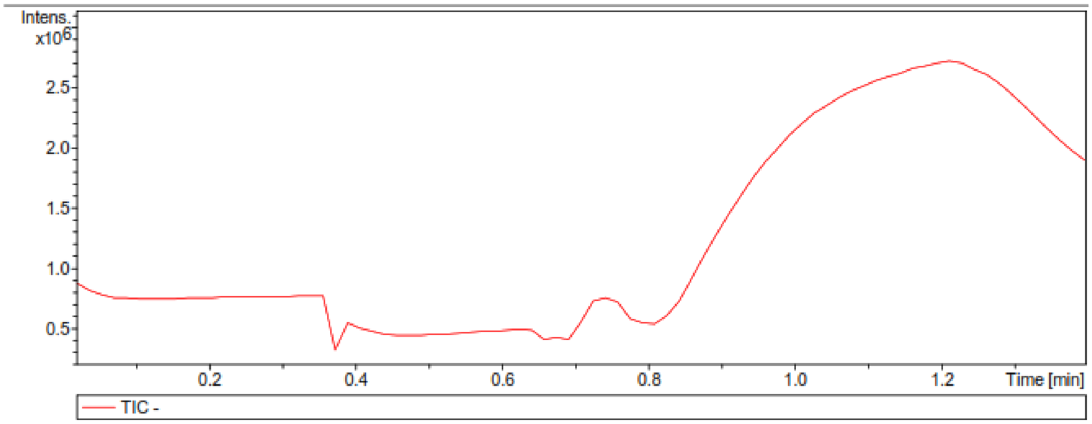

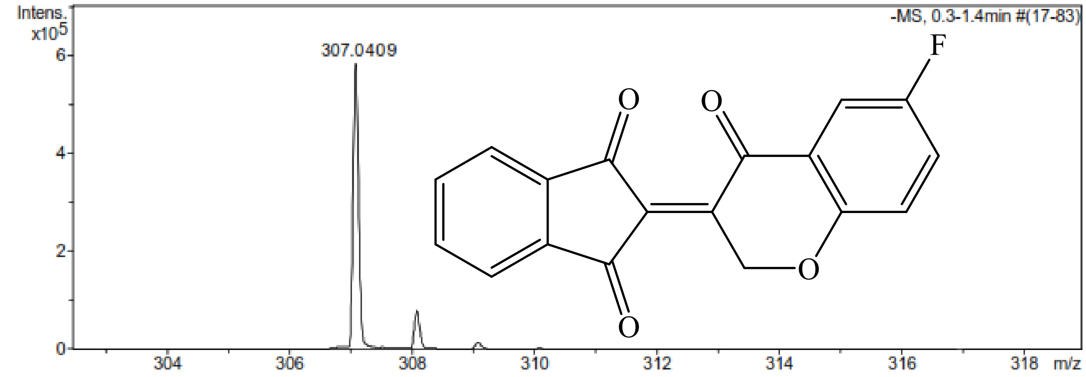

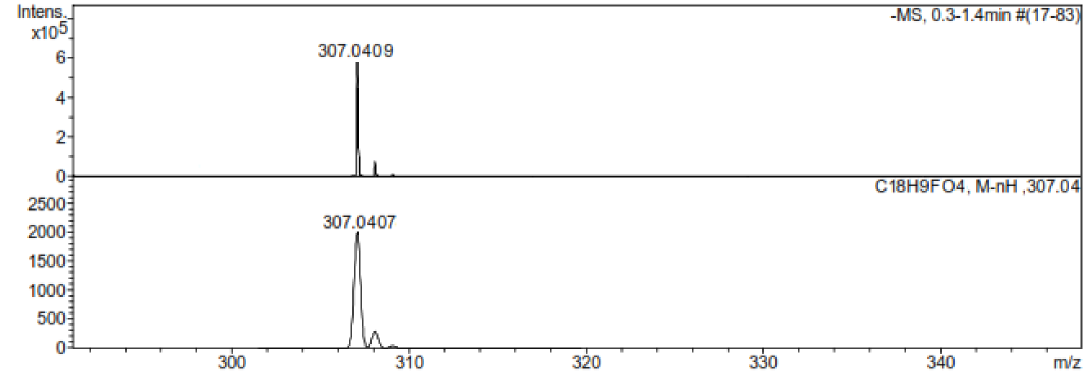

Bruker Compass DataAnalysis 4.0 printed: 2021-12-26 17:04:39 Page 1 of 1

HRMS of **12b**
